# Structure Driven Design of Novel Human Ether-A-Go-Go-Related-Gene Channel (hERG1) Activators

**DOI:** 10.1371/journal.pone.0105553

**Published:** 2014-09-05

**Authors:** Jiqing Guo, Serdar Durdagi, Mohamed Changalov, Laura L. Perissinotti, Jason M. Hargreaves, Thomas G. Back, Sergei Y. Noskov, Henry J. Duff

**Affiliations:** 1 Libin Cardiovascular Institute of Alberta, University of Calgary, Calgary, Alberta, Canada; 2 Centre for Molecular Simulation, Biochemistry Research Cluster, Department of Biological Sciences, University of Calgary, Calgary, Alberta, Canada; 3 Department of Biophysics, School of Medicine, Bahcesehir University, Istanbul, Turkey; 4 Department of Chemistry, University of Calgary, Calgary, Alberta, Canada; Dalhousie University, Canada

## Abstract

One of the main culprits in modern drug discovery is apparent cardiotoxicity of many lead-candidates via inadvertent pharmacologic blockade of K^+^, Ca^2+^ and Na^+^ currents. Many drugs inadvertently block hERG1 leading to an acquired form of the Long QT syndrome and potentially lethal polymorphic ventricular tachycardia. An emerging strategy is to rely on interventions with a drug that may proactively activate hERG1 channels reducing cardiovascular risks. Small molecules-activators have a great potential for co-therapies where the risk of hERG-related QT prolongation is significant and rehabilitation of the drug is impractical. Although a number of hERG1 activators have been identified in the last decade, their binding sites, functional moieties responsible for channel activation and thus mechanism of action, have yet to be established. Here, we present a proof-of-principle study that combines de-novo drug design, molecular modeling, chemical synthesis with whole cell electrophysiology and Action Potential (AP) recordings in fetal mouse ventricular myocytes to establish basic chemical principles required for efficient activator of hERG1 channel. In order to minimize the likelihood that these molecules would also block the hERG1 channel they were computationally engineered to minimize interactions with known intra-cavitary drug binding sites. The combination of experimental and theoretical studies led to identification of functional elements (functional groups, flexibility) underlying efficiency of hERG1 activators targeting binding pocket located in the S4–S5 linker, as well as identified potential side-effects in this promising line of drugs, which was associated with multi-channel targeting of the developed drugs.

## Introduction

Novel therapeutic interventions are required to control heart rhythm disturbances. One promising strategies is to increase the magnitude of potassium currents which underlie normal cardiac repolarization. Pharmacologic binding of small molecule “activators” to the hERG1 (*KCNH2* or Kv11.1) potassium channel is such an example. These activators might be useful in suppressing drug-induced, disease-induced or mutation- induced Long QT Syndromes. Remediating components of the cardio-toxicity observed in retro-viral, anti-cancer, anti-fungal, antibiotic and antipsychotic drugs by multi-pharmacology interventions containing specific channel activators may be essential for recovery of cardiac function [1], [2]. In addition, it was originally proposed that the endogenous hERG1 tail current, resulting from recovery from C-type inactivation, could reinforce phase-3 repolarization and thus may protect from spurious depolarizing forces associated with depolarization-mediated arrhythmias [3]. Thus enhancing the hERG-related tail current could be intrinsically anti-arrhythmic [4]. NS1643 is one of the best-characterized and potent activators of hERG1 [5]–[8]. The molecular mechanism(s) by which activators mediates its pharmacologic effects remains controversial [7]–[12]. Low concentrations of NS1643 (10 µM) increase the magnitude of the tail current whereas higher concentrations (20–30 µM) pharmacologically block the channel [13]. In addition, progressive increase in concentration above 10 µM produced near-linear increases in the leftward shift in the V_1/2_ of activation. In contrast, the effect of NS1643 to shift the voltage-dependence of C-type inactivation of the hERG1 channel developed at 3 µM; with no further increment at higher concentrations. While location of the unique binding site for hERG1 openers is debatable, previous structural and functional studies indicate the possibility of multiple binding sites for activator in the hERG1 channel [7], [12], [13]. The additional evidence for multiple binding sites relates to biphasic concentration-response relationship in response to NS1643.

Recent docking studies combined with electrophysiological studies led to identification of three potential binding sites: one near the selectivity filter; one at the S4 and S4–S5 linker and another in the inner cavity of the hERG1 pore domain [7], which is an obvious culprit for agonist design. Numerous experimental studies indicate that binding to the inner pore of the channel results in the pharmacologic block of hERG1 [14], [15], while binding to the site at the S4–S5 linker appears to contribute substantially to channel activation [7]. The mutations at the E544, within the S4–S5 linker region, increased the NS1643-induced shift in the V_1/2_ of activation and exaggerated slowing of deactivation [7]. Therefore, we have at least one established activator site and a swarm of structural models enabling rational design of specific channel activators with NS1643 as a template. For the first time, it is possible to assess whether molecules designed to bind selectively to the proposed activator-specific site would have unique pharmacologic effects. The hypothesis tested in this study is that designer drugs that interact in the neighborhood of the activation gate would change V_1/2_ of activation and deactivation without substantial pharmacologic block of hERG1. Accordingly this study focuses on design of molecules that interact with hERG1 in the neighborhood of E544 within the S4–S5 linker. We propose that the neighborhood of E544 is an attractive potential binding site for the following reasons:

1. Previous studies indicate that the S4–S5 linker is fundamentally involved in the activation process [16].
2. The E544L mutation substantially increased pharmacologic response to NS1643. Specifically the NS1643 -induced shift in the V_1/2_ of activation was −18±3 mV in wild type (WT) versus −24±3 in E544L [7]–[12].

The NS1643- induced slowing of deactivation was 1.9 fold for WT but 3.5 fold for E544L. In addition the NS1643-induced increase in tail current amplitude was 13% for WT and 398% for E544L. Therefore we reasoned that a drug designed to interface with the neighborhood of E544 might selectively affect activation and deactivation of hERG1. We focus on mechanism(s) of action of NS1643, by designing modifications of its structure to selectively target domains of the hERG1 channel. A combined simulation/experiment strategy was used to design a molecule that selectively binds to a site in the neighborhood of E544 within the S4–S5 linker. Here we find that a drug designed to interface with the neighborhood of E544 selectively affects activation and deactivation of hERG1. Our combined simulation/experiment strategy to design a molecule that selectively binds to a site in the neighborhood of E544 within the S4–S5 linker suggests that this is a potential “druggable” site for activators that can be used to manipulate channel gating.

## Experimental and Computational Methods

### Ligand-Based and Receptor-Based Models on the Designing of Novel Compounds

**Figure 1:**
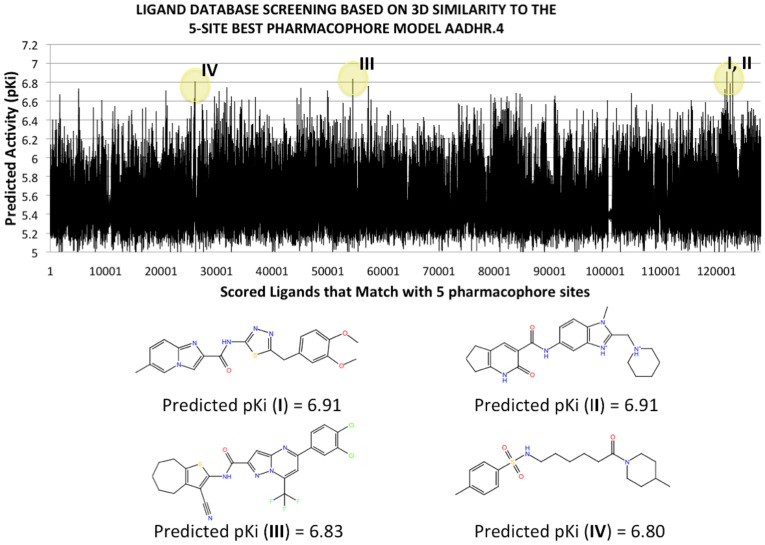
Combined computational approach to opener's design. (Top) Example of 500000 ligands screening from Asinex Small Molecule library. Their activities are assessed with developed 5-site pharmacophore model AADHR.4. (Compounds with low predicted pKi values, i.e., pKi<5.0 are not shown). (Bottom) Selected top-scored compounds are shown together with predicted pKi values.

Several available small molecule databases (i.e., PUBCHEM, ZINC, Asinex) have been screened using our previously developed atomistic receptor based hERG1 model. (Figure 1, and Figures S1–S5 in File S1). The summary of all computational results for ligand screening and optimization is provided in Tables S1 to S4 in File S2. Together with available structure databases a combined ligand- and receptor-based model for the *in silico* design of novel compounds was also used. The fragment database of Schrodinger (∼4000 small-compound database) was used for construction of novel structures to be used for molecular docking. Together with different binding combinations of the fragments with 10 enumeration sites for the chosen template e.g. NS1643, the total number of derivatives was ∼40000. (Figure S1 in File S1) The relevance of the NS-derivative and therefore consideration for the future design were evaluated by assessing intra-cavitary binding (QSAR model developed previously) and statistical model for activity developed from available literature data on hERG1 activators. The rationale for derivatives design and details on 5-site QSAR model AADHR.4 can be found in Tables S3 and S4 in File S2 and Figures S1–S6 in File S1.

### Molecular Docking

**Figure 2:**
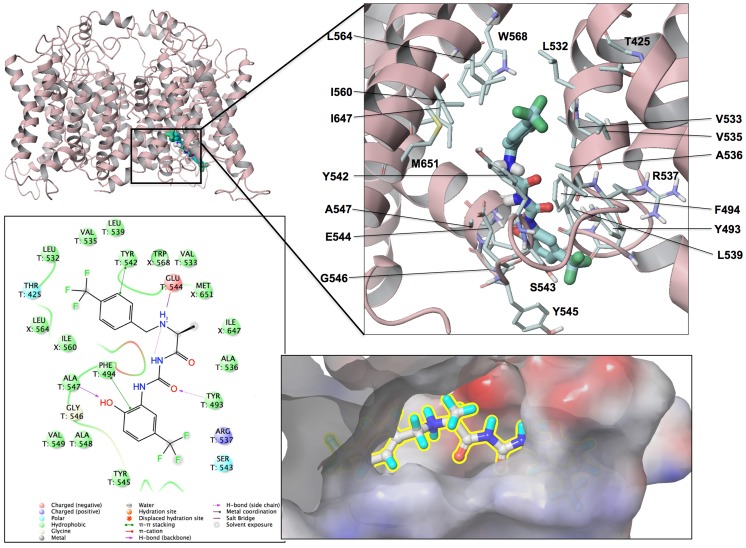
Mapping of the bound conformations for MC-II-157c at the S4–S5 domain of the receptor. 2D ligand interactions diagram (left-bottom panel) and surface representation of docked pose (right-bottom panel) are also shown at the figure.

The docking studies were performed using open and closed states of the target structure [17], [18]. Glide-XP (Grid-based Ligand Docking with Energetics, extra precision) [19] and Induced Fit Docking (IFD) together with Generalized Optimized ligand Docking (GOLD) [20] were used. SiteMap utility from Schrödinger software package [21] was used to obtain information on the location and character of the potential binding sites. The site maps generated were then used to define the different grids for each binding site found, and were used to do the docking with Glide/XP and GOLD. The docking results for all derivatives considered in this study is summarized in Table S1 in File S2. Example of binding pose and site definition can be found in Figure 2. The details of the used docking algorithms, quantum-mechanical (QM) computations used to evaluate ionization energies and stable conformations along with protocols are provided in Methods S1. Table S2 in File S2 summarizes all of the results of QM computations for selections of stable ionization states and conformers for the synthesized compounds. Table S5 in File S2 provides comparison to available experimental data on fragment-homologs. The QM-optimized geometries for synthesized compounds are shown in Figures S7 to S9 in File S1 for substitutions in the positions R1 to R3, respectively. The corresponding energy iso-contours, dipole moments and electron density distributions are shown in Figures S9 to S12 in File S1.

### Synthesis of NS1643 Analogs

**Figure 3:**
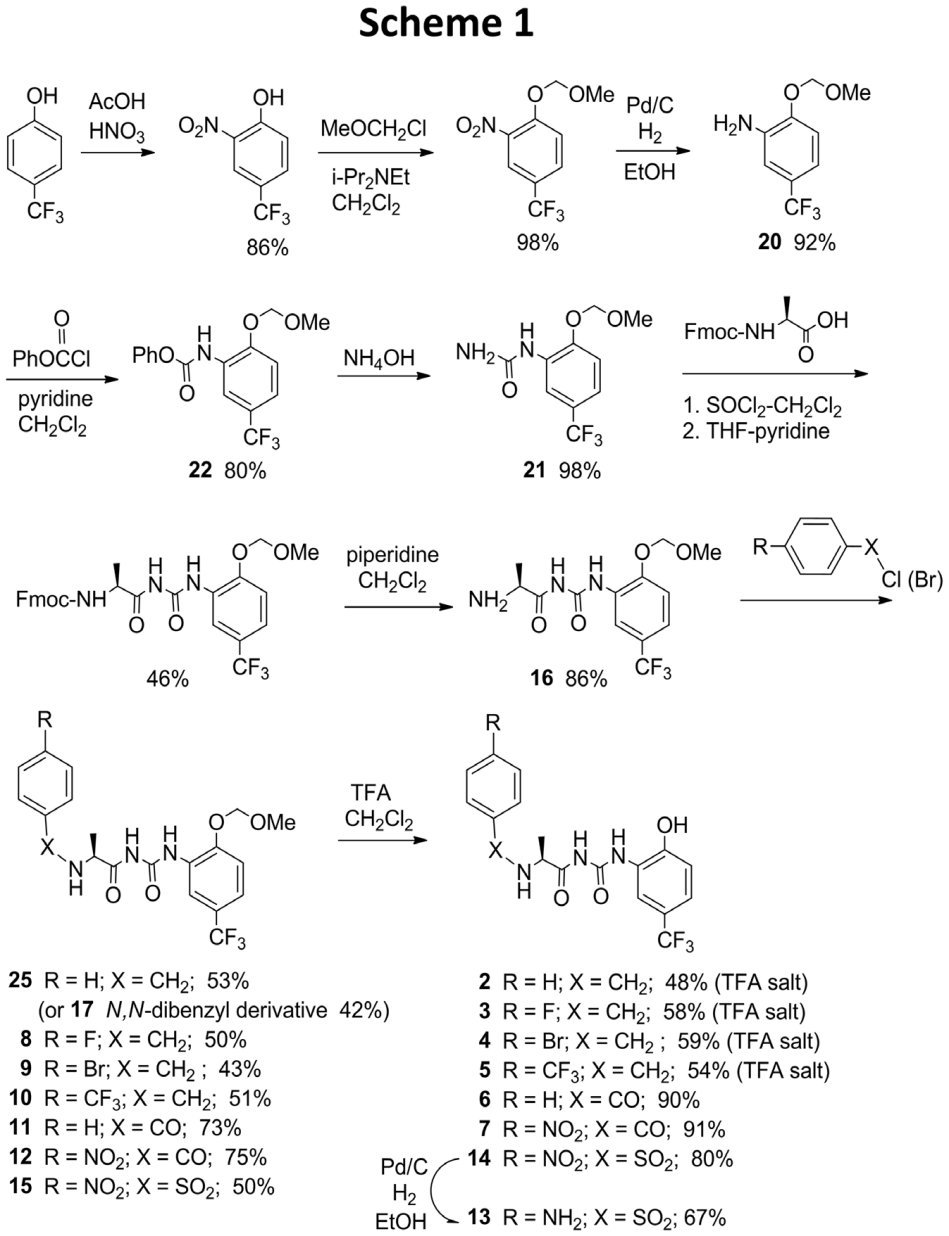
Synthesis of NS1643 Analogs and Intermediates (Scheme 1).

**Figure 4:**
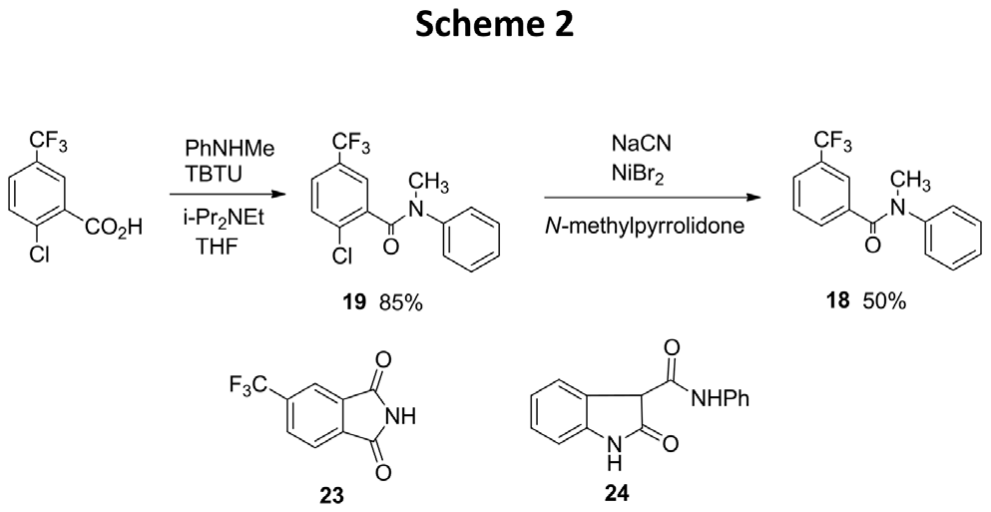
Synthesis of NS1643 Analogs and Intermediates (Scheme 2).

The synthesis of NS1643 analogs is shown in Figures 3 and 4, respectively. All tested compounds for which elemental analyses were not provided were of >95% purity, as determined by HPLC analysis, except for **2**, **10**, **14**, **17**, **18**, **23** and **24**, for which NMR spectra are provided in the Supporting Information. HPLC analyses were performed under the following conditions: Novapak C_18_ reversed-phase column, 3.9×150 mm; solvent: acetonitrile-water, 70∶30, 0.8 mL/min; UV detector: 254 nm. ^1^H NMR spectra were obtained at 300, 400 or 600 MHz. ^13^C NMR spectra were obtained at 75, 101 or 151 MHz. ^19^F NMR spectra were obtained at 376 MHz with hexafluorobenzene (−164 ppm) as the external standard, relative to trichlorofluoromethane (0.00 ppm). The syntheses of compounds **3**–**15** and **17** were performed by minor variations of the methods employed for the preparation of compounds **25** and **2**. The full disclosure on the preparation and characterization data for **3**–**15** and **17** are provided in Synthesis S1.

### Electrophysiology

Transfected HEK cells on glass cover slips were placed in a chamber mounted on a modified stage of an inverted microscope. The chamber was superfused at a rate of 2 mL/min with a normal external solution. The extracellular solution contained (in mM) NaCl 140, KCl 5.4, CaCl_2_ 1, MgCl_2_ 1, HEPES 5, glucose 5.5, pH 7.4, with NaOH. Micropipettes were pulled from borosilicate glass capillary tubes on a programmable horizontal puller (Sutter Instruments, Novato, CA). Standard patch-clamp methods were used to measure the whole cell currents of hERG1 mutants expressed in HEK 293 cells using the AXOPATCH 200B amplifier (Axon Instruments) [22]. Unless otherwise indicated, tail currents were recorded when the voltage was returned to −50 mV from +50 mV. Further details on electrophysiological methods are collected in Methods S1.

Action potentials were recorded from neonatal (day 1) mouse ventricular myocytes as previously reported [23]. Although I_Kr_ has little physiologic importance to the action potential of the adult rodent heart, we previously reported that I_Kr_ is the dominant repolarizing current in day 1 neonatal myocytes, with little or no I_Ks_ [23]. Thus, these neonatal cells are well suited to assess the physiologic relevance of blockade of I_Kr_ to the action potential characteristics. In contrast, to address the possibility that compounds inadvertently interact with other channels, action potentials were recorded from adult cardiac myocytes. Myocytes on glass coverslips were placed in a chamber mounted on a modified stage of an inverted microscope.

### Statistical Analysis

Statsview (Abacus Concepts, Berkeley, CA) was used to analyze the data. The data are presented as the mean ± SE. An unpaired Student's t-test was used to compare the data. A two-tailed p-value of 0.05 was designated as being significant.

Because of limited space, detailed information of methods for chemical synthesis, electrophysiology, generation of 3D QSAR models, selection criteria for generated ligand databases and available ligand libraries, approaches to molecular docking, and a short summary of QM computations can be found in Methods S1.

## Results

### Development of Non-Blocking hERG1 Activators

**Figure 5:**
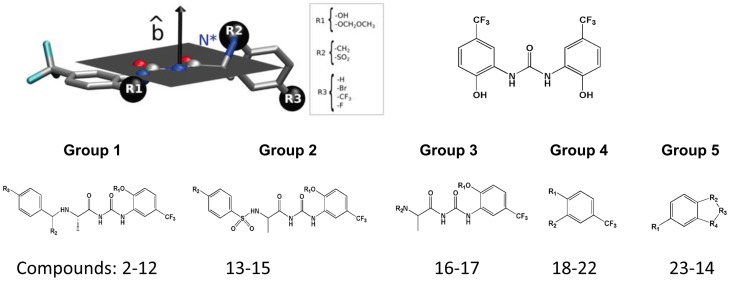
Schematic representation of the studied compounds topology showing the different R1, R2 and R3. The groups were identified to be critical determinants of high-affinity/high-specificity binding of activator to site located in S4–S5 linker of the hERG1 channel. Atom N* depicted in blue represents tentative protonation site. The black arrow represents the versor (∧b) perpendicular to the plane defined by atoms N, C, O, N, C and O of the polyamide moiety, common structure element present in all molecules structure. Top panel shows NS-1643 and chemical group identification. Bottom panel illustrates compound groups synthesized.

The development of non-blocking ligands for hERG1 with improved ability to target tentative opener's site is viable route to structure-inspired opener's design. In the previous study we suggested the possibility of multiple binding sites for NS1643 [7], [13]. Binding of NS1643 to the central cavity site appears to mediate pharmacologic block of hERG1 [13]. In the present study we focus primarily on a binding site in the neighborhood of E544, which appears to be involved in slowing of deactivation and shifts in the voltage-dependence of activation. To generate the initial statistical model for QSAR analysis and rational drug design of channel openers we investigated key interactions between ligands from the small-molecular database (i.e., Asinex, ZINC) and the receptor using previously identified binding pockets in hERG1 [7]. A two-steps drug docking performed with blind docking (i.e., a whole receptor is used as the active site) on a coarse grid covering the entire region of S4–S5 linker of hERG1 in open state [24]. The regions with high-density of bound states identified in docking were analyzed and further refined with high-precision and fine-grid docking simulations. A combined ligand- and receptor-based model for *in-silico* design of novel compounds is considered to develop pharmacophore model for a range of NS1643 derivatives. The Schrodinger's molecular modeling packages fragment database was used to generate of modifications of selected template starting compounds (i.e., NS1643, MC-I-159b, MC-II-43c). (Figure S1 to S6 in File S1). All compounds were then screened in silico for their predicted ability to block hERG1 using a previously build pharmacophore model by our group [25] and a receptor-based model (Glide/XP docking scores to the central cavity of hERG) developed [17], [18]. Compounds that have low-affinity were selected for synthesis and are collected in Table 1. Electrophysiological measurement results of these novel compounds are listed at Table 2. Table 3 shows comparison of the best poses found with Glide/XP and GOLD docking programs for selected drugs binding to the binding pockets at different regions of hERG1 (i.e., S4–S5 linker site close to E544, outer mouth of selectivity filter site, EC domains, and pore domain). The subset of candidates was chosen for chemical synthesis based on the docking studies and a targeted modifications of the functional moieties responsible for channel blockade and binding to established activator site, respectively. Several substitutions in the positions labeled R1 to R3 in Figure 5 were found to produce desired effect *in silico* screening. The ideal (desired effect) target for synthesis from *in silico* modeling entails lowered intra-cavitary affinity compared to original NS1643 and enhanced ability to target previously identified binding pocket around E544. The use of fragment-based approach in the design of the improved activators allows for blinded drug-development thus offering foot-printing of the activator binding pocket in hERG1. The details of the synthesis of the various molecules are provided in File S1/Synthesis S1 and in Figures 3 and 4. The list of substitution groups selected on the basis of in-silico screening and collection of synthesis intermediates is shown in Figure 5 together with definition of compound groups.

**Table 1:** NS1643 and its synthesized derivatives are used in the construction of PHASE Pharmacophore Model.

Comp. No	Name	2D structure	Dock. Score (kcal/mol)	Glide pKi	Phase pKi	# of Confs.
**1**	NS1643		−9.01	6.56	6.17	30
**Group 1**						
**2**	MC-II-43c	R_1_: -H, R_2_: -H, R_3_: -H	−8.56	6.24	6.27	164
**3**	MC-II-163c	R_1_: -H, R_2_: -H, R_3_: -F	−8.00	5.83	6.25	176
**4**	MC-II-159c	R_1_: -H, R_2_: -H, R_3_: -Br	−10.64	7.75	7.79	166
**5**	MC-II-157c	R_1_: -H, R_2_: -H, R_3_: -CF_3_	−11.41	8.31	7.92	144
**6**	MC-II-61c	R_1_: -H, R_2_: = O, R_3_: -H	−10.14	7.39	7.13	48
**7**	MC-II-63c	R_1_: -H, R_2_: = O, R_3_: -NO_2_	−9.68	7.05	7.12	48
**8**	MC-II-161b	R_1_: -CH2OCH3, R_2_: -H, R_3_: -F	−9.02	6.57	7.17	142
**9**	MC-II-155b	R_1_:-CH_2_OCH3, R_2_: -H, R_3_: -Br	−9.38	6.83	7.24	69
**10**	MC-II-153b	R_1_:-CH_2_OCH_3_, R_2_: -H, R_3_: -CF_3_	−10.19	7.42	7.28	129
**11**	MC-II-57c	R_1_: -CH_2_OCH_3_, R_2_: = O, R_3_: -H	−9.17	6.68	6.57	173
**12**	MC-II-59c	R_1_: -CH_2_OCH_3_, R_2_: = O, R_3_: -NO_2_	−8.69	6.33	6.35	39
**Group 2**						
**13**	MC-I-159b	R_1_: -H, R_2_: -NH_2_	−9.73	7.09	7.38	165
**14**	MC-I-169b	R_1_: -H, R_2_: -NO_2_	−9.59	6.99	7.29	125
**15**	MC-I-155b	R_1_: -CH_2_OCH_3_, R_2_: -NO_2_	−7.64	5.57	6.22	86
**Group 3**						
**16**	MC-I-153b	R_1_: -CH_2_OCH_3_, R_2_: -H	−9.12	6.64	6.44	12
**17**	MC-II-67b	R_1_: -CH_2_OCH_3_, R_2_: -(CH_2_C_6_H_5_)_2_	−8.74	6.37	6.51	177
**Group 4**						
**18**	MC-I-165b	R_1_: -H, R_2_: -CO-(NCH_3_)C_6_H_5_	−7.15	5.21	-	10
**19**	MC-I-163b	R_1_: -Cl, R_2_: -CO-(NCH_3_)C_6_H_5_	−6.51	4.74	-	7
**20**	MC-I-89b	R_1_: -OCH_2_OCH_3_, R_2_: -NH_2_	−7.41	5.40	5.21	4
**21**	MC-I-93b	R_1_: -OCH_2_OCH_3_, R_2_: -NH-CO-NH_2_	−7.73	5.63	5.66	7
**22**	MC-I-167b	R_1_: -OCH_2_OCH_3_, R_2_: -NH-CO_2_-C_6_H_5_	−6.95	5.06	5.20	15
**Group 5**						
**23**	MC-I-161b	R_1_: -CF_3_, R_2_: CO, R_3_: -NH, R_4_: CO	−7.20	5.25	5.42	3
**24**	MC-II-17c	R_1_: -H, R_2_: NH, R_3_: CO, R_4_: CH-CO-NH-C_6_H_5_	−8.03	5.85	5.65	4

Table includes calculated (from Glide/XP) and predicted pKi values of compounds as well as number of conformers of each compound used in the construction of models. 2D structures for compound groups are shown in Figure 5.

**Table 2:** Summary of electrophysiologic effects for the novel molecules studied.

Comp No.	Name	Conc. µM	Amplitude I/I_con_	Activation V_1/2_ (mV)	Inactivation ΔV_0.3_	Deactivation Δλ/λ_con_
**1**	NS1643	10	1±0.04	−21±2.3	+6±0.6	1±0.4
**2**	MC-II-151c	20	0.4±0.02	−14.5±5.5	−21±0.0	0.6±0.4
**3**	MC-II-163c	20	0.6±0.14	−17.3±0.3	−7.5±4.5	1.7±0.3
		2	0.6±0.14	−17.3±0.3	−7.5±4.5	1.7±0.3
**4**	MC-II-159c	20	0.6±0.07	−33±14	−4.5±1.5	3.1±1.6
		2	0.76±0.14	−5±2	−0.5±0.5	0.25±0.02
**5**	MC-II-157c	50	0.83±0.1	−27.5±3.5	26±1	4.5±0.1
		20	0.86±0.1	−15.3±4.1	+9.3±2.0	3.5±0.2
		10	0.88±0.1	−14±3.6	14.0±2.7	3.3±1.3
		3	1.1±0.04	−6.5±0.5	1±1	0.8±0.1
		1	0.95±0.02	−2±0.0	1.3±0.9	0.2±0.1
**6**	MC-II-61c	20	0.7±0.02	−3.7±0.7	−2.5±1.5	0.8±0.1
**7**	MC-II-63c	20	0.6±0.1	−7±1.1	−3.5±4.5	0.6±0.3
**8**	MC-II-161b	20	0.1±0.0	−12.5±2.5	−4±8	−0.1±0.04
**9**	MC-II-155b	20	0.5±0.1	−9.1±2.1	−3.5±1.5	−0.1±0.1
**10**	MC-II-153b	20	0.4±0.02	−13.7±3.2	−12.3±3.4	0.5±0.03
**11**	MC-II-57c	20	0.6±0.04	−12.7±2.7	−1.5±1.5	−0.1±0.2
**12**	MC-II-59c	20	0.6±0.19	−6.3±2.7	−7.3±1.9	−0.1±0.1
**13**	MC-I-159b	50	1±0.03	−2.5±3.0	+10±0.6	0.5±0.1
		20	1.0	−5±3	+8±0.4	0.3±0.1
		2	1.0	−2±3	n/a	
**14**	MC-I-169b	50	0.39±0.04	−7.7±3.0	2.5±3.5	0.6±0.3
		2	0.93±0.01	−3±0.02	n/a	0.4±
**15**	MC-I-155b	30	0.5±0.04	0±0	−10±0	−0.01±0.07
		10	0.7±0.12	−3±3	−11±2	−0.1±0.1
**16**	MC-I-153b	50	0.3±0.02	−19±3	−27±2	0.003±0.14
		10	0.6±0.06	−7±2	n/a	−0.07±0.1
**17**	MC-II-67b	50	0.95±0.03	−2±0	+4±0	0.25±0.18
		10	±0.01	−5±0.6	−1±0.6	0.07±0.08
**18**	MC-I-165b	50	0.8±0.07	3.3±0.3	−11.7±7.3	−0.2±0.05
**19**	MC-I-163b	50	0.7±0.06	1±3	−2±1	−0.2±0.08
		5	0.9±0.1	0	n/a	
**20**	MC-I-89b	100	0.9±0.04	−3.3±1.3	−1±0	−0.06±0.2
		5	0.02	−2.5±0.5	0±1	0.03±0.01
**21**	MC-I-93b	50	0.9±0.04	−1.3±2.4	0.7±3.7	0.02±0.1
**22**	MC-I-167b	50	0.9±0.04	−1.7±2.3	0±0	−0.2±0.09
**23**	MC-I-161b	50	1.0±0.01	−2.3±1.3	−2±1.2	0.2±0.1
**24**	MC-II-17c	50	0.8±0.03	−2.3±0.9	8±0	0.2±0.2

**Table 3:** Comparison of the best poses found with Glide/XP and GOLD for selected drugs binding to open hERG1 channel.

Site\Drug	NS1643	MC-II-159c	MC-II-157c	MC-II-153b	MC-I-169b
**IC1** (S4–S5 linker)	XPScore (Glide) kcal/mol				
	−7.25	−7.61	−8.61	−6.76	−6.62
	ChemScore (GOLD) kcal/mol				
	−7.13 (64)	−8.90 (83)	−7.24 (53)	−7.12 (100)	−7.69 (100)
**SF** (outer mouth)	XPScore (Glide) kcal/mol				
	−5.8	−5.13	−6.54	−6.48	−4.28
	ChemScore (GOLD) kcal/mol				
	−6.32(38)	−6.52(69)	−7.68(61)	−6.80(35)	−6.06(57)
**EC2**	XPScore (Glide) kcal/mol				
S3–S4	−4.72	−5.34	−6.47	−6.39	−4.25
	ChemScore (GOLD) kcal/mol				
S2–S1	−7.14(98)	−6.75(74)	−7.43(87)	−8.46(83)	−8.11(72)
					
**Pore Domain (PD)**	XPScore (Glide) kcal/mol				
S5–S6	**−7.46**	**−4.92**	**−4.70**	**−5.83**	**−6.31**

In parentheses is the population of the cluster from which the best pose comes from (always most populated one). In the case of Glide/XP, the output is only giving the selected best poses using a selection criteria explained in the methods. For definitions of the binding pockets see Durdagi et al. [7].

### Electrophysiological Evaluation of Designed Molecules

**Figure 6:**
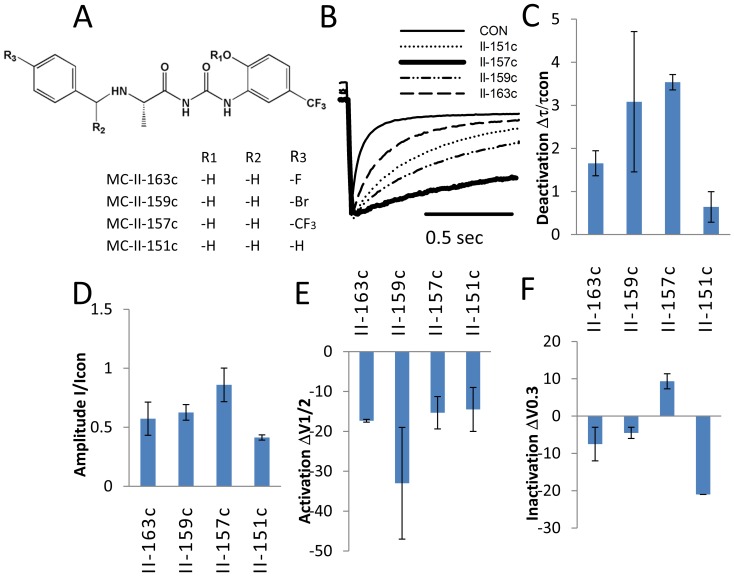
Substitutions in benzene ring (position R3) are determinants of prolongation of deactivation. Electrophysiologic responses to four molecules, MC-II-163c, MC-II-159C, MC-II-157b, and MC-II-43c are compared. Panel A shows the structures. All four molecules are similar except for the substituents on benzene ring #2. MC-II-43c is unsubstituted, whereas MC-II-163c, MC-II-159C, MC-II-157b are *para* substituted with fluoro, bromo and tri-fluoro groups respectively. Panel B shows raw examples of the slowing in the deactivation rate. Panel C shows the deactivation time constants relative to the base lines. Panel D shows the magnitude of the tail current relative to baseline. Panel E shows the shift in the voltage-dependence of activation and Panel F shows the shift in the voltage-dependent of inactivation. Panels C-F show no significant differences between the molecules except for their effects on deactivation. All 4 molecules shifted voltage dependence of activation to hyperpolarized potentials.

**Figure 7:**
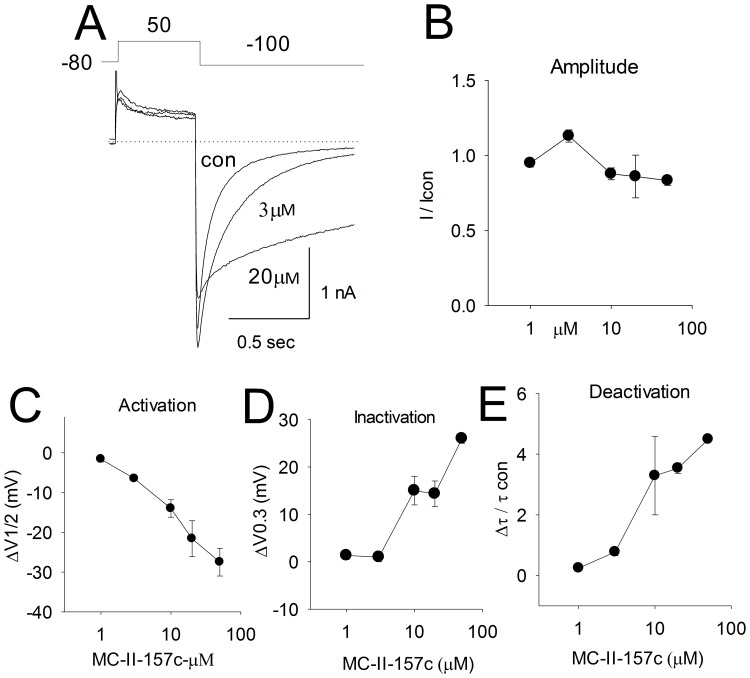
The concentration-response relationship of MC-II-157c. Panel A shows the raw currents elicited by the protocol shown in the inset. Panels B-E show the concentration-response relationships for mean tail current amplitude (Panel B), mean Δ shift in V_1/2_ of activation (Panel C), mean Δ shift in V_1/2_ of inactivation in Panel D and mean prolongation of the deactivation in Panel E.

**Figure 8:**
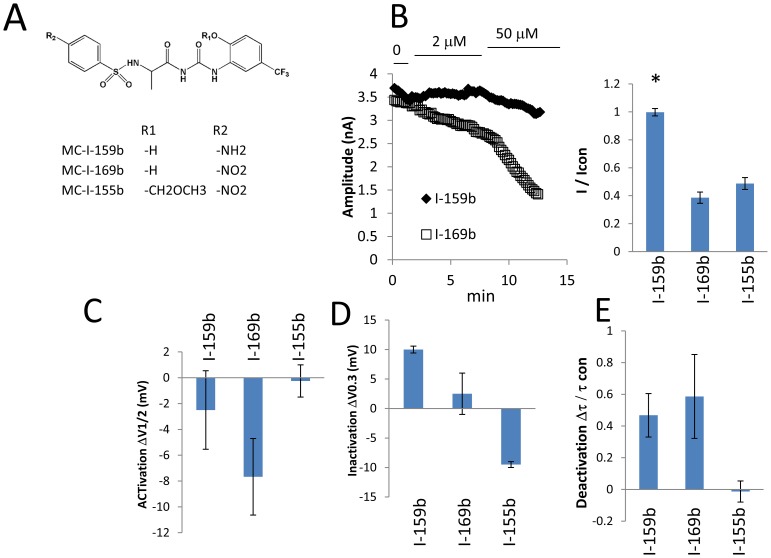
Concentration dependence of compounds MC-I-159b, MC-I-169b and MC-I-155b. Panel A: Chemical structures of compounds are shown. Panel B: Time dependent changes of tail current magnitudes in response to different drug concentrations. Panel C: The magnitude of the tail current relative to baseline. Panel D: The shifts in the voltage-dependence of activation and in the voltage-dependent of inactivation. The deactivation time constants relative to base lines is represented at bottom-right Panel E.

The pharmacologic-responses to the molecules designed and synthesized for this study are shown in Table 2 and Figures 6 to 8. Of all the compounds developed, the *bromo*- substitution (i.e., MC-II-159c) and the *trifluoro*- substitution (i.e., MC-II-157c) produced the greatest decrease in deactivation rate (Figures 6–8). Importantly, both MC-II-159c and MC-II-157c slowed the rate of deactivation substantially more than NS1643. The extent of prolongation of deactivation was greatest for MC-II-159c. At 2 µM, MC-II-159c minimally blocked the channel (I/Icon = 0.76) significantly prolonged deactivation but minimally left shifted the V_1/2_ of activation by −5±2 mV. However at 20 µM it blocked the hERG1 tail current (I/I_con_ = 0.6). For MC-II-157c there is little evidence for concentration-dependent pharmacologic block at the µM range of concentrations. Importantly, at 3 µM, MC-II-157c significantly increased the tail-current amplitude to an extent similar to that seen with NS1643. At 10 µM the normalized current (I/Icon) was 0.88; at 20 µM the I/Icon was 0.86 and at 50 µM the I/Icon was 0.83. Indeed these values are comparable to that seen during prolonged placebo treatment (I/Icon = 0.89). Prolonged whole-cell dialysis of cells can result in run-down of the hERG1 current. Moreover, we never observed increase in current during drug washout. Interestingly MC-II-157c produced monotonic concentration-dependent shifts in the V_1/2_ of activation and slowing of deactivation. The magnitude of the effects on activation and deactivation are approximately 6-10 fold greater than that previously reported with NS1643 signifying relevance of the proposed binding site at the S4–S5 linker and viability of the rational design for openers.

### Pharmacologic Responses to MC-II-157c and MC-II-159c Are Significantly Less in E544L

**Figure 9:**
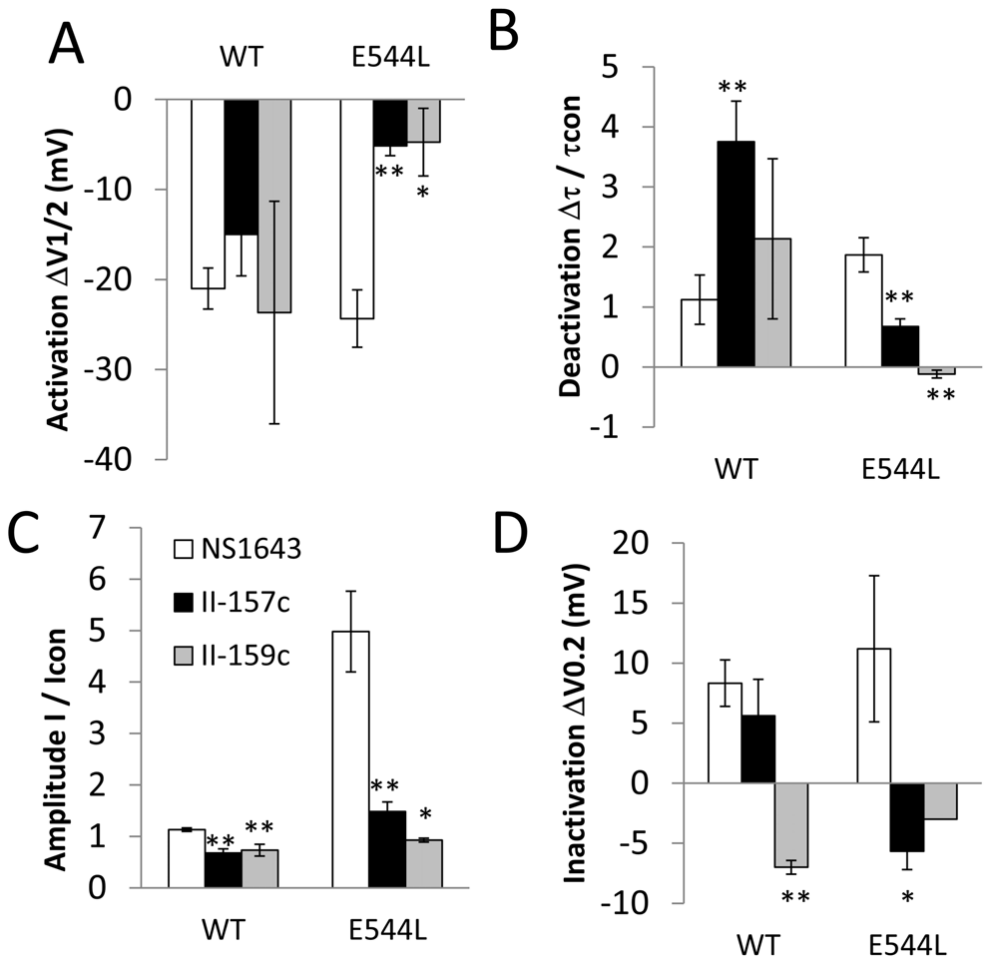
Pharmacologic response (Δ) to NS1643 (open white bars at 10 µM), MC-II-157c (black bars at 10 µM) and MC-II-159C (grey bars at 10 µM) are compared in wild type (WT) versus E544L. Panel A: In WT, NS1643 MC-II-157c and MC-II-159c, all shift voltage-dependence of activation and Panel B: slow deactivation. (top) In E544L, pharmacologic response to NS1643 is exaggerated whereas Δ response to MC-II-157c and Δ MC-II-159c were markedly diminished. (bottom-left) Panel C: In terms of amplitude of the tail current, in E544L response to NS1643 is exaggerated whereas response to MC-II-157c and MC-II-159C is markedly diminished. Pharmacologic response in terms of inactivation is complex. Panel D: In WT, MC-II-157c shifts voltage-dependence of inactivation to depolarized potentials whereas MC-II-159C shifts voltage dependence to hyperpolarized potentials. Pharmacologic response to NS1643 is exaggerated in E544L whereas for MC-II-157c and MC-II-159C responses are diminished. (bottom-right) * evaluates the statistical significance of the Δ response to NS1643 compared to Δ response to MC-II-157c or Δ response MC-II-159c. * designates p<0.05; ** designates p<0.01. n values were: For Activation panel in WT n = 10,8 and 3; for E544L n = 9,6 and 3. For deactivation in WT n = 8,8,3 respectively and for E544L n = 4,6,3. For tail current amplitude, in WT n = 9,8,3 and in E544L n = 8,6,2. For inactivation, n = 9,8,3 and for E544L n = 5,6,2.

Figure 9 compares the pharmacologic responses to NS1643, MC-II-157c and MC-II-159c in hERG1 WT versus E544L. In WT hERG1, MC-II-157c and MC-II-159c like NS1643 slows deactivation and shifts voltage-dependence of activation to hyperpolarized potentials. In E544L, NS1643 produces exaggerated pharmacologic responses with significantly greater magnitudes of shifts in voltage-dependence of activation and slowing of deactivation. However in E544L, MC-II-157c and MC-II-159c produces significantly and substantially less pharmacologic responses. These data provide evidence that MC-II-157c and MC-II-159c are effectively targeting the neighborhood surrounding E544, and that interaction with this neighborhood mediates shifts in voltage-dependence of activation and deactivation. Moreover, these data provide evidence that the neighborhood surrounding E544 appears to be a true binding site. The measurements of voltage-dependence of inactivation for E544L are considered estimates only. For E544L the rate of deactivation is very rapid, with its tau being similar to that of inactivation. Moreover, for E544L, the voltage–dependence of deactivation and inactivation overlap substantially. These elements may confound the accuracy of measurement of inactivation.

### Contribution of the Peptide-Like Side-Chain Linker between the Benzene Rings to Channel Blockade

Several recent studies emphasized importance of drug rigidity for efficient binding to channel with a reduced blockade [26], [27]. We investigated the effect of the peptide-like chain linker between benzene rings. The length of the linker can be used to control flexibility of the designed molecules. This varying length and flexibility of the linker was implemented in the structures of MC-I-153b to MC-II-67b listed in Table 1. The compound MC-I-153b has a single benzene ring with an intact peptide-linker. Interestingly this compound still left shifted the V_1/2_ of activation at 50 µM but produces pharmacologic block at that concentration (I/I_con_ = 0.3). At lower concentration such as 10 µM, the compound still shifts V_1/2_ of activation to hyperpolarized potentials but also had modest capacity to block hERG1 currents (I/I_con_ = 0.6). Compounds **18**–**24** (Table 1) manifest little or no pharmacologic activity even at concentrations of 50 µM. Therefore, it can be concluded that the binding site in the receptor imposes tight dimensional requirements for the size of the peptide linker and additional flexibility in the linker may lead to pronounce intra-cavitary blockade. Similar conclusions were reached in in-silico designs of minimally structured hERG1 blockers, as well as re-designed analogs of high-affinity blocker dofetilide [26], [27].

### Review of Key Features of the Designed Molecules

The pharmacologic responses to the new activators designed herein were compared to the parent compound, NS1643. For the NS1643 molecule, the ratio of the concentration producing channel blockade (30 µM) to the concentration increasing current density (10 µM) is approximately 3∶1. In a previous study, the tail current was increased upon application of 10 µM of the activator [7]. The pharmacologic blockade becomes apparent at 30 µM of NS1643. Such a small therapeutic ratio may not provide a huge safety factor for that compound. Two of the newly synthesized molecules (MC-II-157c and MC-II-159c) had novel and interesting pharmacologic effects. The drugs both slowed deactivation of hERG1 and left-shifted voltage-dependence of activation. Thus for the hERG1 channel MC-II-157c appears to have a reasonable therapeutic ratio with minimal blockade signifying possibility of the rational design of channel's openers. In context, many supposed agonists (increasing tail current density) such as NS1643 also block the channel but do so at higher concentrations^22^. Interestingly the effects on deactivation of MC-II-159c and MC-II-157c were still manifest at lower drug concentrations ∼2 µM. Unlike NS1643, MC-II-159c slows deactivation and shifts voltage-dependence of activation to hyperpolarized potentials but does not shift voltage-dependence of inactivation to depolarized potentials.

The potential dangers of hERG1 channel agonists may relate to off-target binding to other cardiac channels [28], [29]. To address whether these drugs have a specific effect on the hERG1 channel or whether they may target other cardiac channels, we recorded action potentials and examined the effects MC-II-157c and MC-II-159c had on fetal mouse ventricular myocytes. Both MC-II-157c and MC-II-159c affected the upstroke of the action potential, the overshoot potential and the excitability threshold but only at the highest studied concentrations (i.e., 20 µM) as illustrated in Figure 9. At the low concentrations there was no impact of these molecules on the action potential. However at 20 µM, there were clear changes in excitability and the overshoot potential decreased significantly. This manifested a blockade of the sodium current (I_Na_). Therefore MC-II-157c is a hERG1 activation gate modifier with off-site interactions. It does not block hERG1 at 20 µM, but blocks a cardiac sodium channel. It would be difficult to predict this off-target problem if action potentials had not been recorded in this study. Even so, MC-II-157c still does shift voltage dependence of activation at 3 µM, increases tail current amplitude and slows deactivation. Thus the ratio of the concentration modify activation appears to be roughly 6-fold lower than the concentration blocking the sodium current. Such a small ratio does not provide a huge safety factor for specificity of effect, but still considerably better than original safety factor (3-fold) for NS1643. Even so, modification of its design is absolutely necessary to obviate interaction with the sodium channel, as an off-target site.

## Discussion

### hERG1 Activators as New Antiarrhythmic Drugs

There have been few new antiarrhythmic drugs developed in the past 20 years, even fewer if drugs with novel mechanisms are considered. Moreover, the strategy of blocking ion channels has been tested extensively and has universally failed (CAST) [30]. One potentially new approach is the development of hERG activators that increase hERG currents to oppose congenital and acquired Long QT Syndromes (LQTS). Acquired Long QT Syndromes include those induced by pharmacologic block of hERG1 and those induced by heart failure. There are gaps in our knowledge. How can we design drugs that activate the hERG1 channel with minimized propensity to pharmacologic blockade? In addition, some studies report that drugs that shift the voltage-dependence of C-type inactivation to depolarized potentials could increase the time-dependent current at the expense of increasing the tail current. Increasing the time-dependent current could potentially truncate the action potential and create a pharmacologically-induced Short QT Syndrome. Such a prodysrhythmic potential has been reported by Patel et al for PD-118057 [31]. A second question is: -how do we design drugs that modify the hERG1 function without this prodysrhythmic potential? These are complex issues. This study just begins to address some of these issues. In this study we design drugs that shift V_1/2_ of activation and slow deactivation with less propensity to pharmacologically block hERG1 and with less rightward shift in the voltage-dependence of C-type inactivation. However, an unforeseen limitation of our design is that these molecules also block I_Na_ in cardiac myocytes. Further refinements must consider multi-target models, specifically including SCN5a, to obviate off-target activity.

### Chemical Structure of Designed Molecules and Channel Blockade/Activation

It is instrumental to analyze key chemical properties of molecules designed in our work and to relate them to the observed effects on the hERG1 function. The electronic structure computations (Methods S1) suggest that introduction of a sulfonamide moiety has a dramatic effect on the distribution between charged and neutral forms of the drug by stabilizing the neutral form of the drug. This leads to a weaker intra-cavitary blocking ability and therefore represent a desired effect for development of non-blocking molecules. To test whether or not this feature manifests itself *in vitro*, we performed electrophysiological measurements on two synthesized molecules (MC-I-159b and MC-I-169b) from Table 1 containing a sulfonamide moiety between the R3 benzene ring and the peptide- like linker (Figure 8). The compound MC-I-159b, like NS1643 shifted the voltage-dependence of inactivation to depolarized potentials. Importantly at this same concentration (50 µM), the compound did not produce pharmacological block of hERG1 tail current in keeping with predictions from QM computations. At lower concentration (i.e., 20 µM), the drug still significantly shifted voltage-dependence of inactivation to depolarized potentials but at 1 µM it had no pharmacologic activity. The congener MC-I-169b produced significantly more pharmacologic block at 50 µM than seen with MC-I-159b (Figure 8). These data indicate that the exact substituents on the R3 benzene ring (see Figure 5) and the character of the linker to the peptide-like chain are important modulators of electrophysiological activity and support the prediction from QM computations regarding ionization state. An introduction of a different functional group (ester-) suggested by fragment-based design and molecular docking abolish any physiological activity of the designed compounds (#2 to #18 in Table 1). This finding is in keeping with the prediction from QM computations that an ether group changes the compound considerably and, probably, leads to a different mode of binding.

### Molecular Organization of the Binding Pocket for Openers

Current study provides substantial insights on critical interactions responsible for specific interactions with activators. While key-amino acid residues at the binding site for MC-II-157c are Y493, E544, and A547, stabilizing interactions for MC-II-159c involve Y542, E544, A547, and T548 side-chains (Figure 2). The decomposition analysis of binding energies emphasizes importance of π-π stacking interactions between F494 and Y542 side-chains and MC-II-157c. MC-II-159c is stabilized by π -π stacking interactions with Y542. The docked conformations of two high affinity ligands are very similar. (Figures S13 and S14 in File S1). Superimposition of top docking poses of MC-II-157c and MC-II-159c has been given in Figure S14 in File S1. E544 is found to forms multiple polar contacts and stabilizing hydrogen-bonding with high affinity ligands, while not involved in stabilizing low-affinity substrates in the identified binding site (S4-S5 helices). To illustrate interactions responsible for high-affinity binding, we are showing 2D and 3D ligand interactions diagram for one of the low-affinity compounds (MC-I-167b shown in Figure S14 in File S1 and electrophysiological evaluation of its binding effects are shown in Figure S15 in File S1). There are no π-π stacking interactions with the ligand and E544 forms only one hydrogen bond. (Figure S14 in File S1, top-left panel). To provide further evidence that the pharmacologic response to MC-II-157c and MC-II-159c relates to a true interaction with hERG1 in the neighborhood of E544 we assessed whether their pharmacologic responses were disrupted in the E544L mutation. Indeed we found that the pharmacologic responses to MC-II-157c and MC-II-159c were significantly and substantially reduced in E544L hERG. This contrasts with the response to the parent compound, NS1643, which manifests an exaggerated response in E544L. Accordingly, these data provide evidence that the neighborhood surrounding E544 appears to be a true binding site and that binding of MC-II-157c and MC-II-159c to this site mediates slowing of deactivation and the shifts in the voltage-dependence of activation.

### Proposed Mechanism of Action

In this study, we were able to dissociate the pharmacologic effects on activation/deactivation from effects on inactivation by rationally designing opener molecules. The fact that *in silico* modeling can design a congener of NS1643 that selectively affects activation/deactivation but not inactivation suggests that the putative drug pocket in the neighborhood of E544 maybe a genuine binding site for activators. It seems reasonable to assume that another binding site mediates the pharmacologic effects on inactivation. Earlier we had reported another putative binding pocket in the neighborhood of the selectivity filter [7]. It may be reasonable to assume that binding in the neighborhood of the selectivity filter might mediate rightward shift in the voltage-dependence of inactivation. These data are in keeping with our previous study, which had suggested multiple binding sites for NS1643 in the hERG1 potassium channel. Binding of activators in the neighborhood of E544 appears to alter movement of the S4 or the S4–S5 linker mediating changes in activation/deactivation process. This supposition is in keeping with the study of Tristani-Firouzi *et al.* [16] which provide evidence that interactions between the S4–S5 linker and the S6 helix are critically involved in activation/deactivation.

### Classification of Drugs as Activators *versus* Blockers

**Figure 10:**
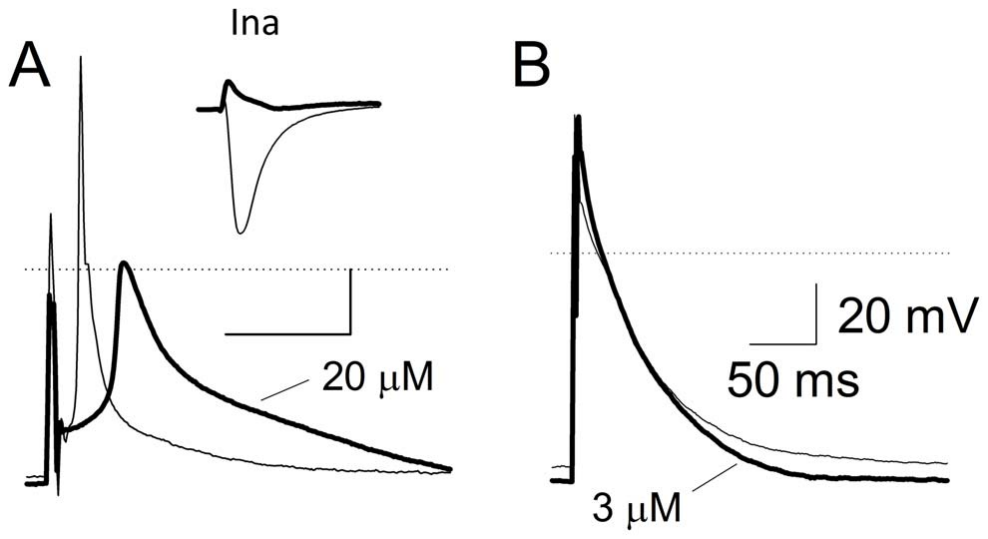
Drug effect on action potential in cardiomyocytes. Panel A. The effect of 20 µM and 3 µM Panel B. MC-II-157c on action potentials neonatal cardiomyocytes. The inset in Panel A shows the effect of 20 µM MC-II-157c on I_Na_ of same kind of cells. In patch clamp studies, the I_Na_ was induced by depolarizations to −30 mV from holding potentials of −100 mV. The light traces are baseline and bold traces were recorded with MC-II-157c.

Any classification of a drug as an activator versus a blocker probably needs to take into consideration the therapeutic ratio e.g. the ratio of the concentration producing hERG1 blockade versus the concentration producing a potentially therapeutic effect [4]. Most activators have a low therapeutic ratio. For example, NS1643 increases the tail current magnitude at 10 µM but produces substantial block at 30 µM. Therefore the therapeutic ratio of that molecule is very approximately 3∶1. For the sake of safety one might hope for a therapeutic ratio of >10∶1 in order to have some confidence that pharmacologic blockade would not occur leading to a myocardial disease and electrolyte disturbance. MC-II-157c produced a modest slowing of deactivation, shifting V_1/2_ of activation to hyperpolarized potentials and a modest increase in tail current amplitude at 3 µM and did not show a concentration-dependent block of hERG1 even at 50 µM. To assess the potential impact of MC-II-157c on the heart, cardiac action potentials were recorded. At 3 µM MC-II-157c shortened action potential duration and its Phase III shape. However, at 20 µM, MC-II-157c substantially blocks I_Na_ an unforeseen off-target effect (Figure 10). Even so, the therapeutic ratio of MC-II-157c as defined as the ratio of concentrations affecting I_hERG_/I_Na_ is approximately 6∶1. Many of the developed hERG1 activators exhibit concentration-dependent channel blockade at higher concentrations [32]. While developed activators show minimal ability to block hERG1 currents at low µM concentrations, it is worthwhile to understand its molecular mechanism. For two potent activators (MC-II-159c and MC-II-157c) we observed minimal blockade at high drug concentrations. High-precision docking for these two compounds was used to better understand molecular determinants of observed blockade (Table 3). The intra-cavitary binding pocket for developed compounds is predominantly formed by T623, S624, S649, Y652 and F656. The hydroxyl groups (i.e., S624 and S649 for MC-II-159c) are predicted to be important for the formation of stable H-bonds with the drug [33] [26]. However, the aromatic moiety F656 is important construct π-π stacking interactions for drug stabilization in the cavity. (Figure S16 in File S1) Mutations in the S6 region, such as F656X, often inhibit the blocking ability of some of the activators (such as RPR260243 and ICA-105574) [4], [6]. Importantly, as molecular analysis of high-affinity binding for activator suggest, very same interactions with a binding site located at the interface S4–S5 are responsible for channel's activation. A strategy that may work the best to increase binding to an “activators” site involves optimization of interactions with charges moieties in the channel (E544) exposed to aqueous solutions.

### Off-Target Interactions by the Existing and Novel Activators

We prospectively designed molecules to avoid substantial blockade of the central cavity of hERG1. Importantly, none of the molecules designed herein substantially blocked the hERG1 current at <10 µM. This suggests that computational models combining QSAR/docking with atomistic models of hERG1 reliably design drugs that avoid high affinity blockade of the hERG1 current. Even so, blockade of this current was observed at concentrations in the range of ∼50 µM. To address whether lead molecule MC-II-157c produces other unpredictable off-target effects we recorded fetal mouse ventricular action potentials before and after treatment with MC-II-157c and MC-II-159c (Figure 10). At high concentrations (20 µM), MC-II-157c decreased the upstroke *dV/dt* of the action potential, the overshoot potential and excitability threshold. The inset of Figure 10 shows that these effects results from blockade of the cardiac sodium current (I_Na_). Even so, MC-II-157c, at low concentration (3 µM), significantly shortened action potential duration (Figure 10, right panel). Thus the ratio of the concentrations that modify deactivation of the hERG1 current is 6-fold lower than the concentration required to block the cardiac sodium current. Further modifications of MC-II-157c are required to ablate its interaction with the cardiac sodium channel. Therefore, our work raises awareness of this unforeseen issue when designing hERG1 activators. Further studies may need to take into consideration alternative ion channels as targets. The crystal structure for the bacterial homo-tetrameric voltage-gated Na^+^ channel from *Arcobacter butzleri* (NavAb) has been reported however, the structure for SCN5a has yet to be described and is expected to be very different from that of NavAb [34]. It is important to mention that issue of multiple receptors for existing drugs, while often ignored, is well known and represent significant challenge in targeted drug development [35].

## Conclusions

The study had two prospectively defined goals: 1) To use *De Novo* drug design-assisted synthesized NS1643 analogues based on 3D QSAR design and molecular docking or available NS-like drugs from available drug databases to the neighborhood of E544 to identify molecules that shifted voltage- dependence of activation to hyperpolarized potentials and slowed deactivation without shifting the voltage dependence of inactivation. 2) To avoid pharmacological block of the hERG1 current at low micromolar concentrations. These goals were successfully achieved in this study. The therapeutic ratio between amplification of current density and hERG1 blockade was improved from 3∶1 to almost 10∶1. Therefore, our study showed that it is possible to selectively target activation/inactivation of hERG1 channel with small molecule. One of the designed molecules, MC-II-157c, substantially shifted the voltage-dependence of activation and slowed deactivation greater than that observed with NS1643. However, at concentrations of (>20 µM) it blocked I_Na_. The side effect, reported for the first time in our study, may be common to other activators. It would be difficult to predict or even to observe this off-target problem if action potentials had not been recorded in this study in fetal mouse ventricular myocytes. Inadvertent blockade of off-target ion channels not considered in the design represents a challenge for *in silico* design of drugs. Multi-ion channel targets will need to be considered in future designs.

## Supporting Information

File S1 **Figures S1 to S16 present pharmacophore model evaluation, docking results and additional experimental data on drug effect on hERG1 currents.**
(PDF)

File S2 **Tables S1 to S5 disclosing development of pharmacophore model, QM computations and details of ZINC database search.**
(PDF)

Methods S1 **Detailed description of computational and experimental methods used in the paper.**
(PDF)

Synthesis S1 **Chemical synthesis schemes for each compounds together with quality control data (NMR, HPLC data).**
(PDF)
