# Supplementary material for: Structure Driven Design of Novel Human Ether-A-Go-Go-Related-Gene Channel (hERG1) Activators
Source: PLoS One. 2014 Sep 5;9(9):e105553. doi: 10.1371/journal.pone.0105553 (PMC4156305; doi:10.1371/journal.pone.0105553)
Supplement: File S1 — Figures S1 to S16 present pharmacophore model evaluation, docking results and additional experimental data on drug effect on hERG1 currents. (PDF) [file pone.0105553.s001.pdf]

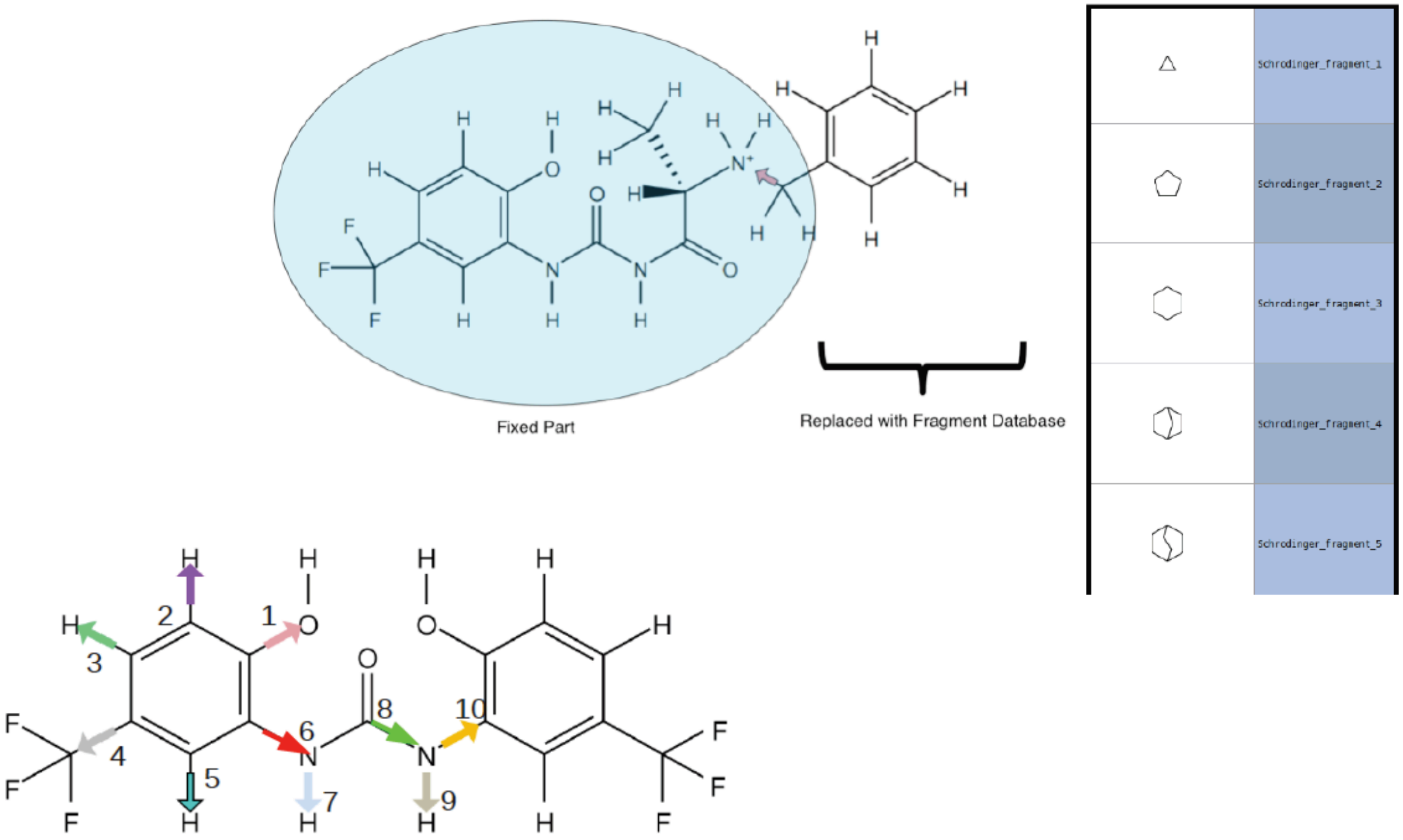

Figure S1. Construction of NS1643 derivatives. (top) Around 4000 small-compound database is used for the production of new NS derivatives. (bottom) 10 enumeration sites are used to generate new derivatives. Together with different binding combinations of the fragments, total number of derivatives reached to ~40000.

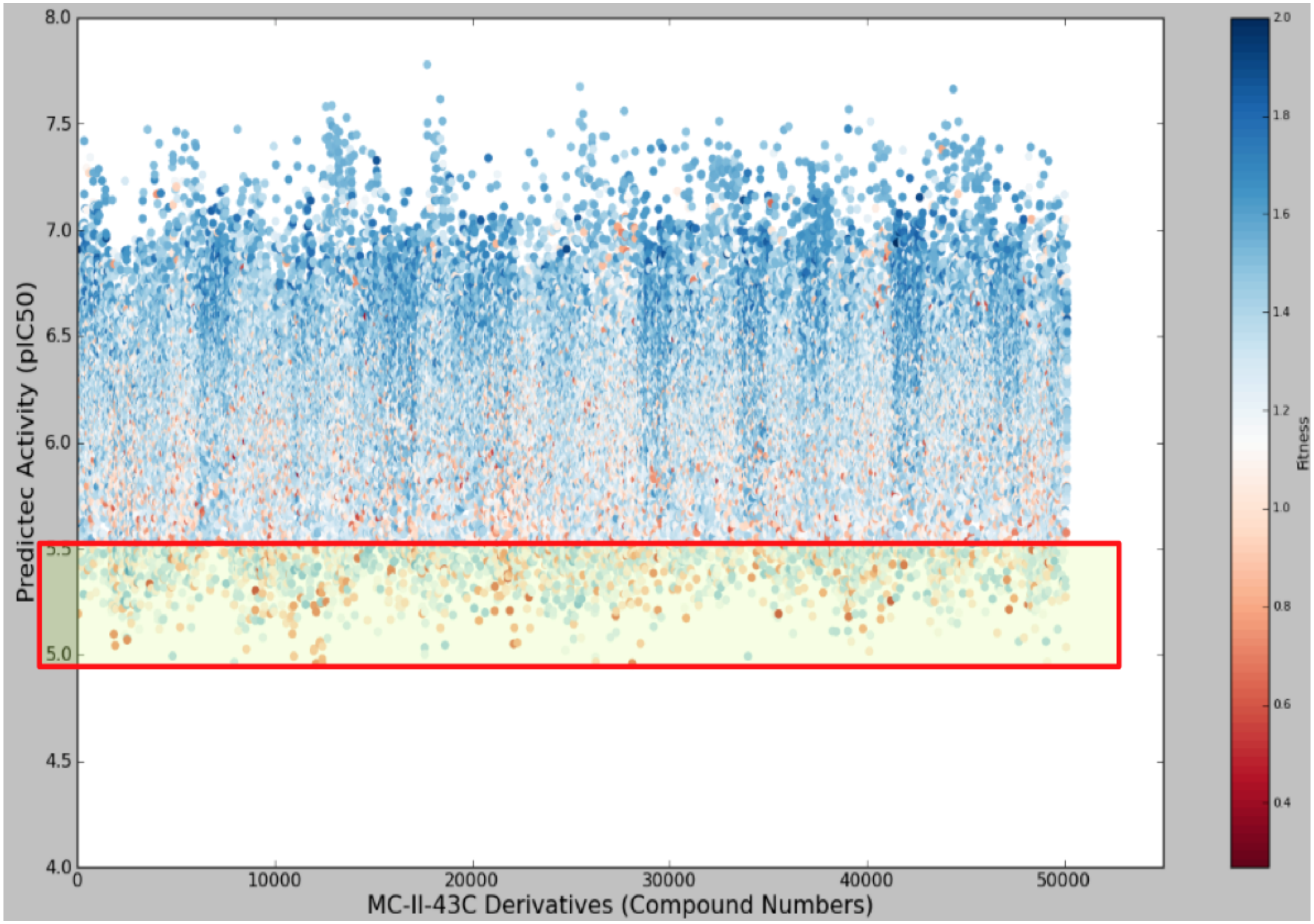

Figure S2. Derived ligands are screened at our previously reported hERG pharmacophore model for predicting their hERG blocking profiles. Compounds that have low predicted pIC_{50} values as well as low Fitness scores (low hERG blocking activities) were selected (highlighted region) for next step (Docking).

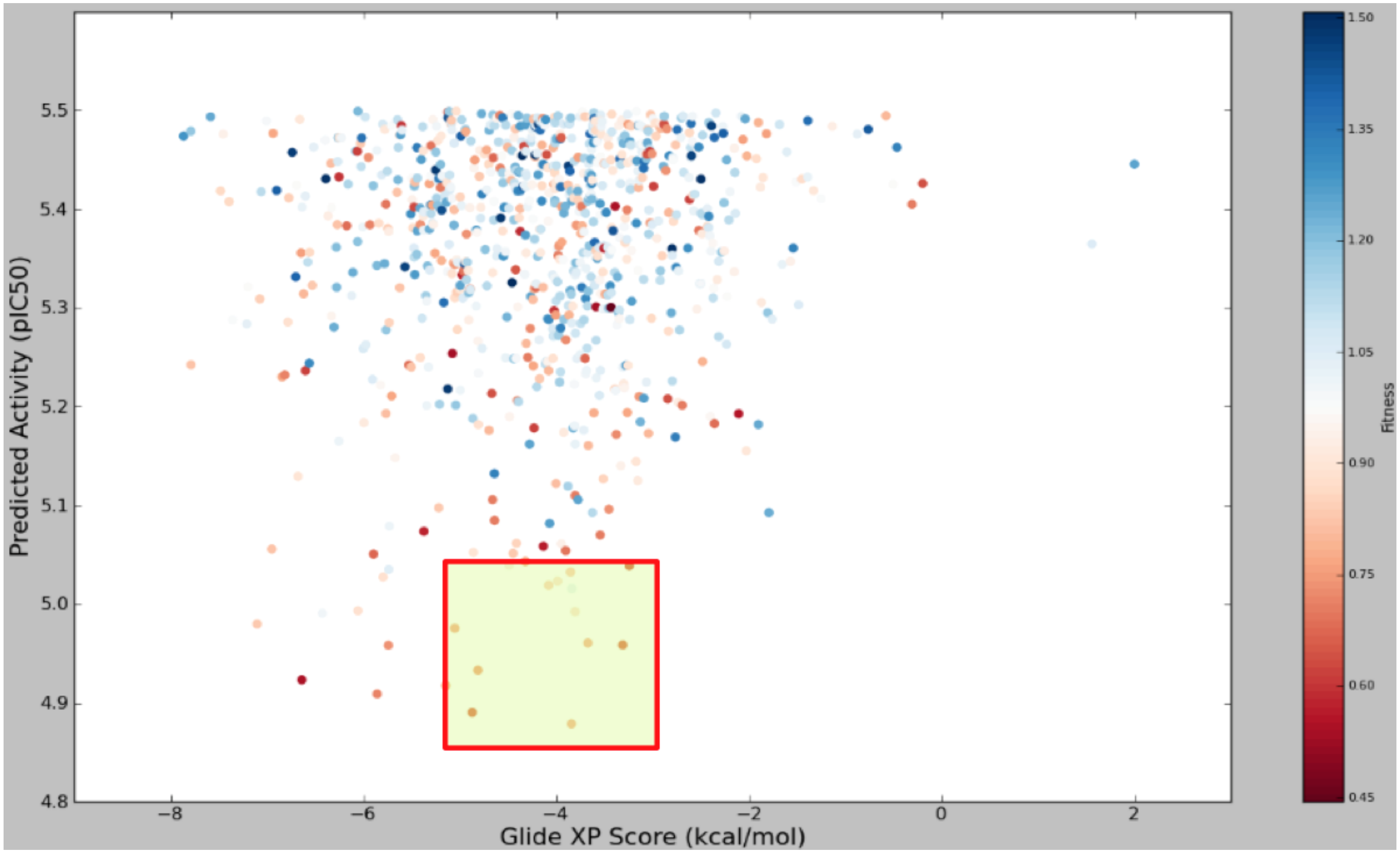

Figure S3. Combination of Ligand- and Receptor-based scoring. Selected compounds from pharmacophore modeling (see Figure S2) are docked at the central cavity of hERG1, the structures that carry low-docking score together with low pIC_{50} and low Fitness score profile are selected for ligand docking at the S4S5-linker binding site.

Derivative No.	2D- Structures	Central Cavity Docking Score (kcal/mol)	Predicted pIC50	Fitness Score	E544 Region Docking (XP) Score (kcal/mol)
MC-II-43C-SD1	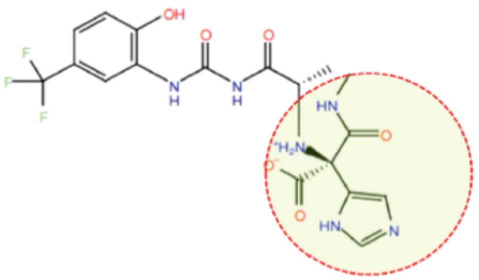	-3.67	4.96	0.813	-6.86

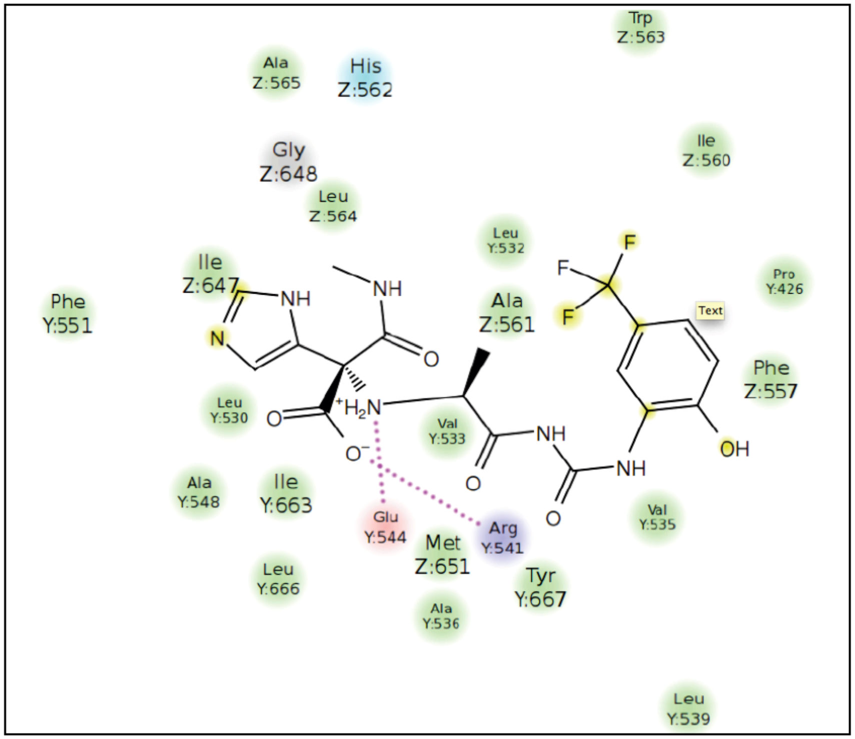

Figure S4. (top) Selected Ligand from Combination of Ligand- and Receptor-based Screening. Figure shows it's predicted pIC_{50} value and Fitness score at hERG pore domain by pharmacophore model; and it's central cavity as well as S4S5 linker site (i.e., E544 site) docking by Glide/XP. (bottom) Ligand interaction diagram of selected ligand at the S4S5 site.

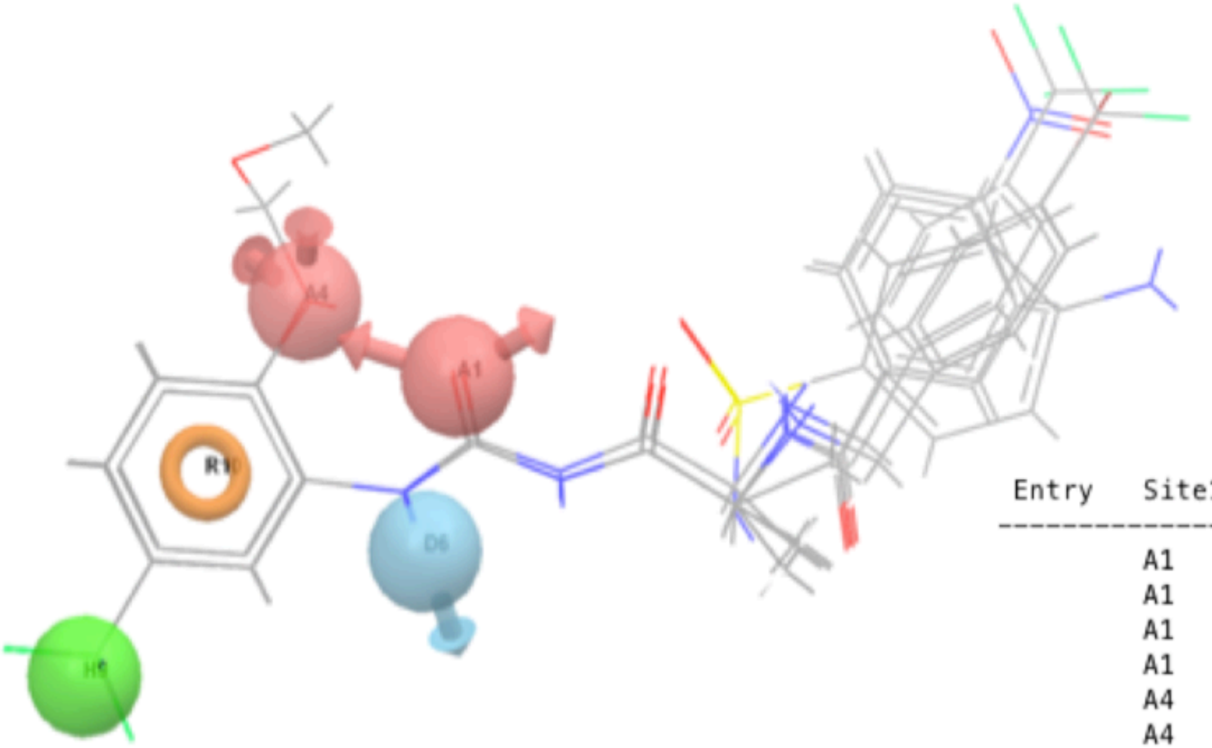

Entry	Site1	Site2	Distance
	A1	A4	3.084
	A1	D6	3.144
	A1	H9	6.501
	A1	R10	3.893
	A4	D6	3.667
	A4	H9	6.065
	A4	R10	2.780
	D6	H9	5.404
	D6	R10	3.387
	H9	R10	3.286

Figure S5. Superimposition of pharmacophore sites at active compounds. Distances between each site have been shown at right panel of the figure as inset.

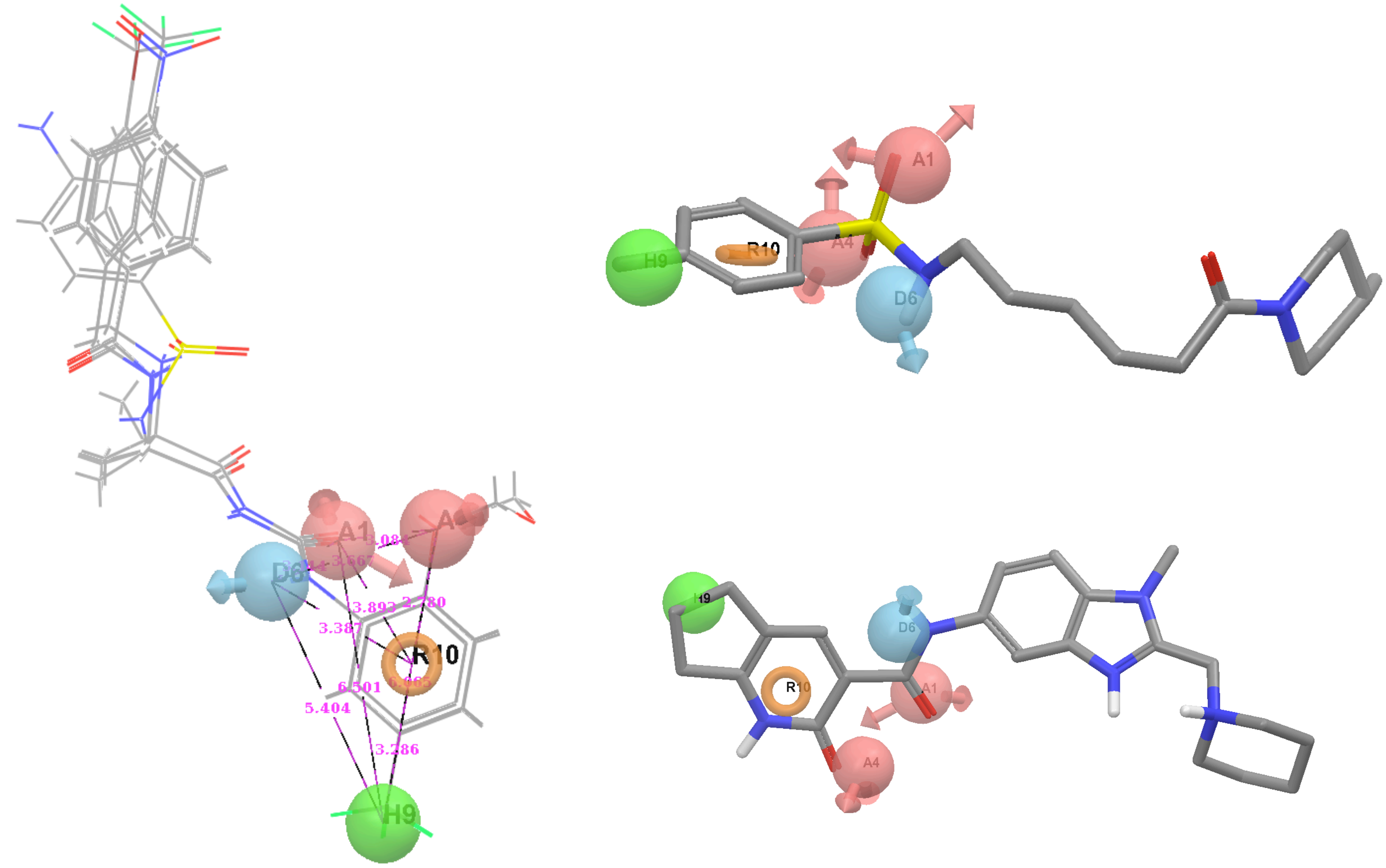

Figure S6. Alignment of 5-pharmacophore sites with active ligands (left) and selected ligands from hit-screening (right).

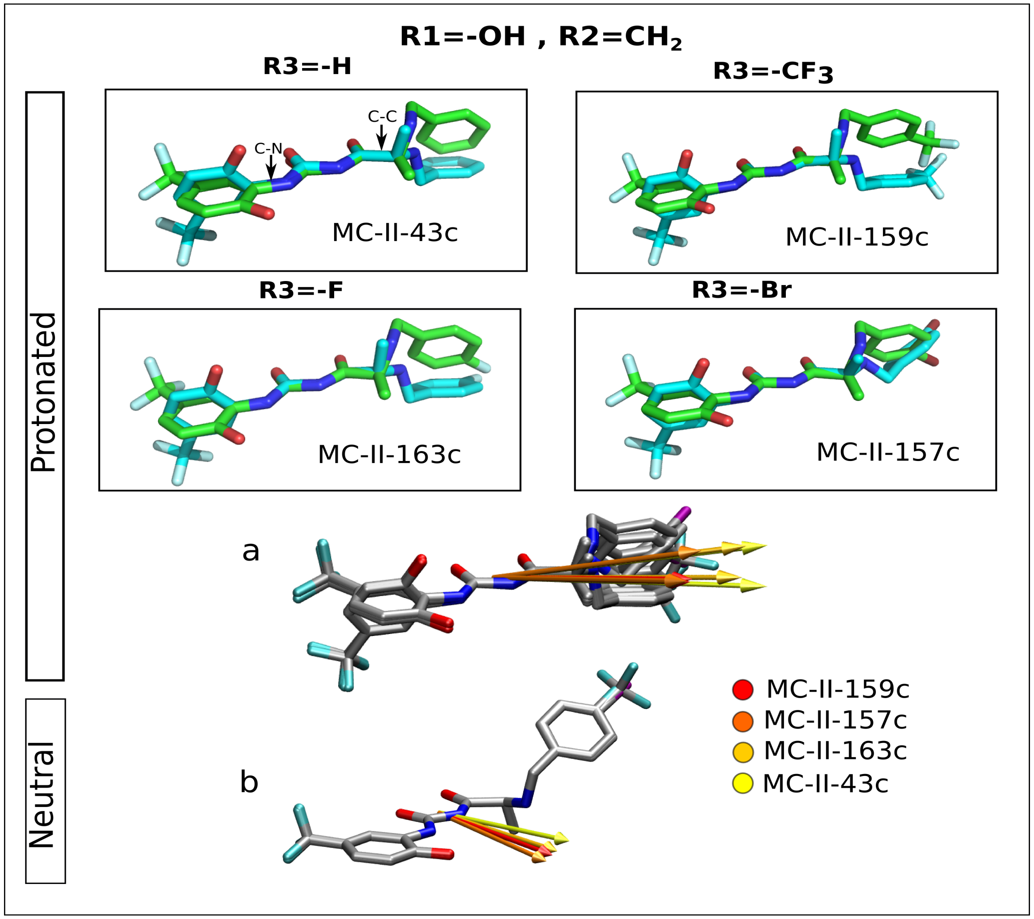

Figure S7. Optimized structures and dipole moment for drugs bearing R1=OH, R2=CH2 and R3=H, Br, CF3 and F; in both protonation states. The two conformers found for the protonated state, 1 and 2 are superimposed and shown in cyan and green respectively. Dipole moment together with the corresponding optimized drug structure are shown in different colors for all superimposed drugs for protonated (a) and neutral (b) states.

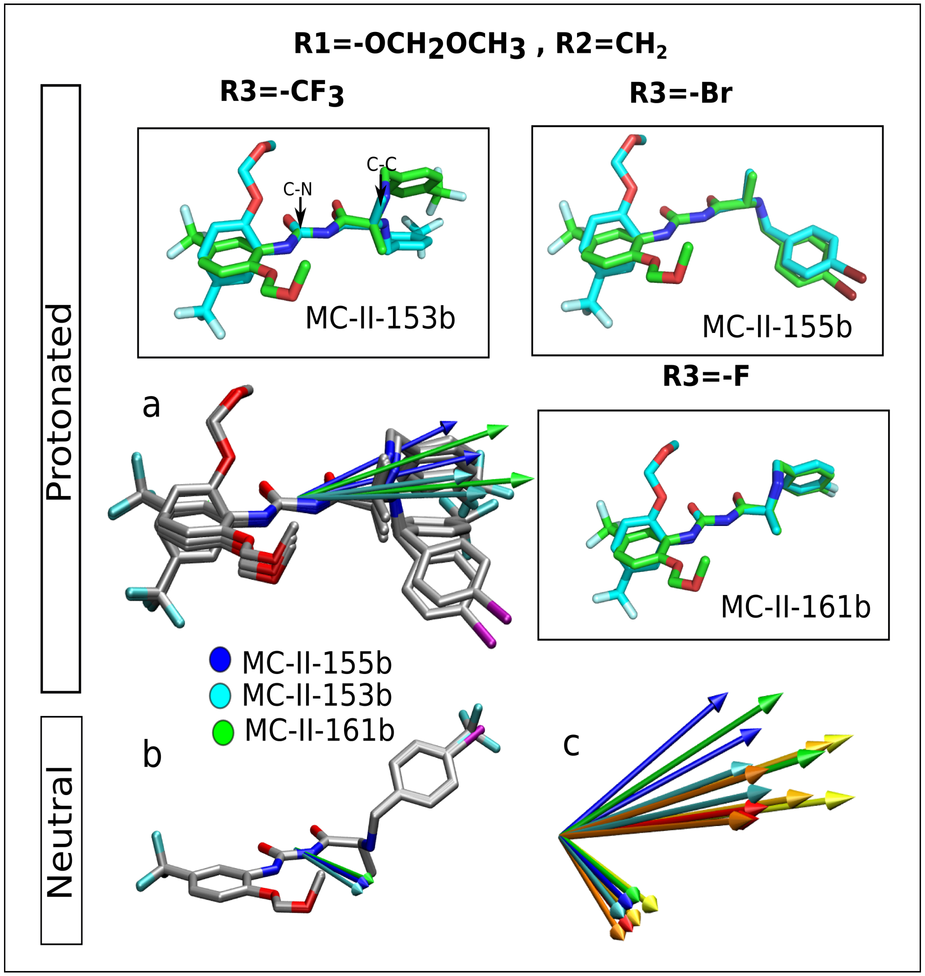

Figure S8. Optimized structures and dipole moment for drugs bearing R1=OCH2OCH3, R2=CH2 and R3=Br, CF3 and F; in both protonation states. The two conformers found for the protonated state, 1 and 2 are superimposed and shown in cyan and green respectively. Dipole moment together with the corresponding optimized drug structure are shown in different colors for all superimposed drugs for protonated (a) and neutral (b) states. Comparison of dipole moments for all conformations for the hydroxyl and ether series of compounds (c).

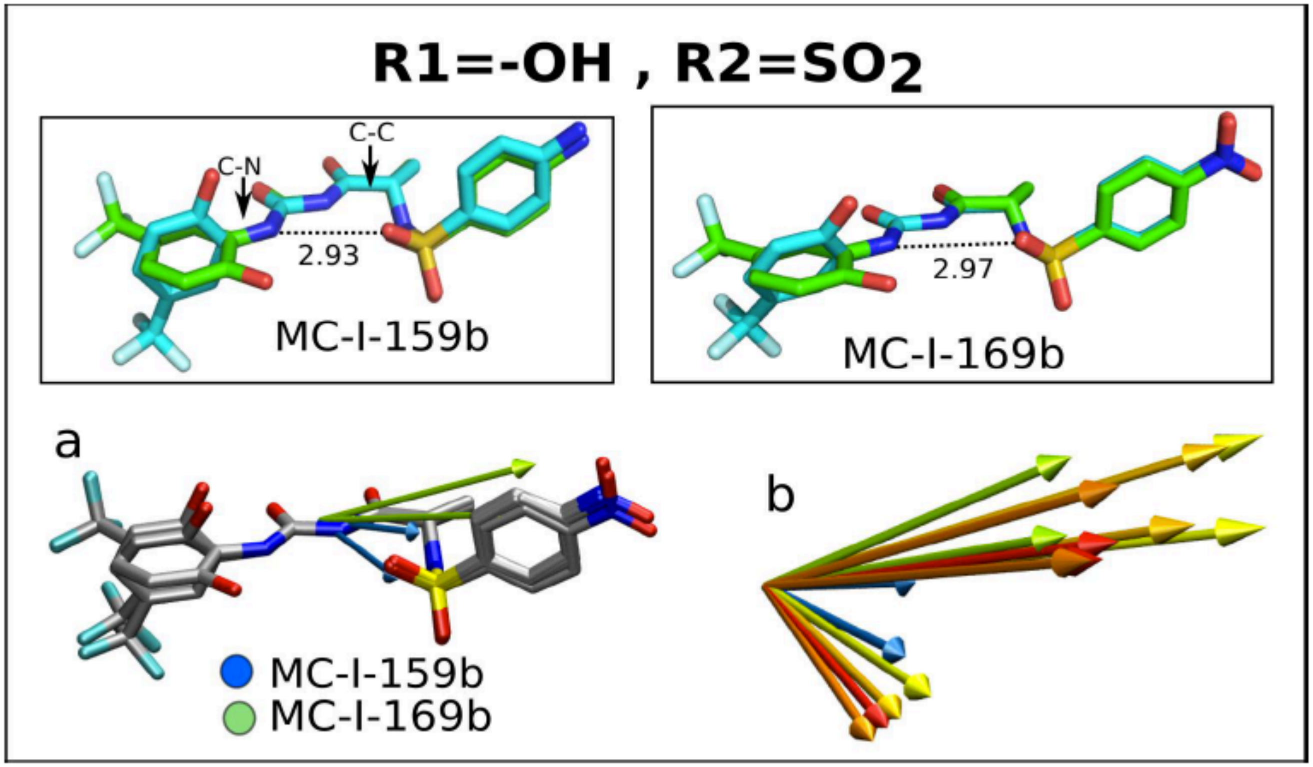

Figure S9. Optimized structures and dipole moment for drugs bearing R1=OH, R2=SO2 and R3=NH2 and NO2. The two conformers found, 1 and 2 are superimposed and shown in cyan and green respectively. Dipole moment together with the corresponding optimized structure are shown in different colors (a). Comparison of dipole moments for all conformations for the hydroxyl and sulfonamide series of compounds (b).

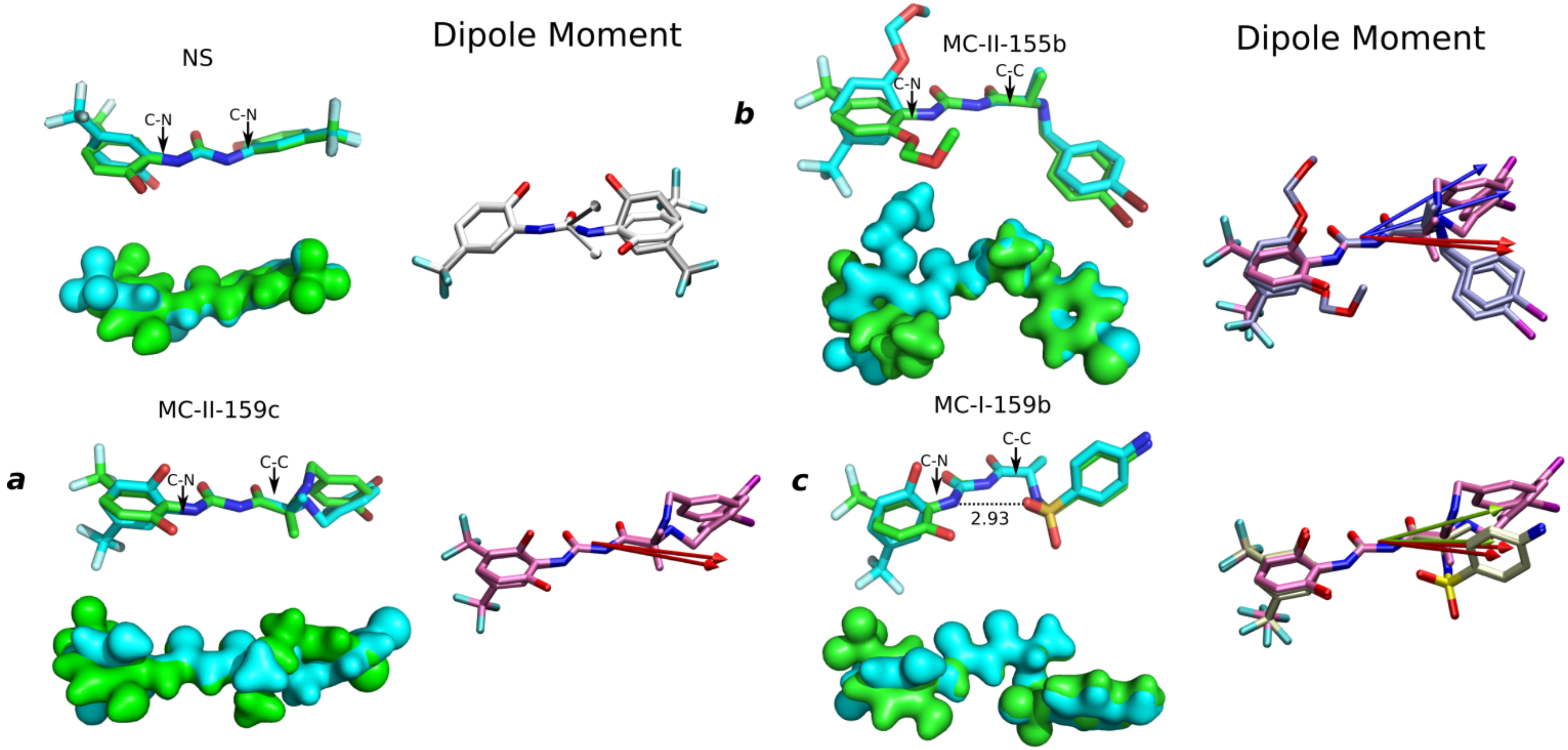

Figure S10. Structure, electron density (Isosurface contour is 0.05) and dipole moment for NS and one drug selected from each group (**a**, **b**, **c**). Both thermodynamically feasible conformers found for the drugs are superimposed and shown in cyan and green respectively. For drugs MC-I-155b and MC-I-159c only the protonated state is shown. In the cases of MC-II-155b and MC-I-159b dipole moment and structures are superimposed and compare to MC-II-159c.

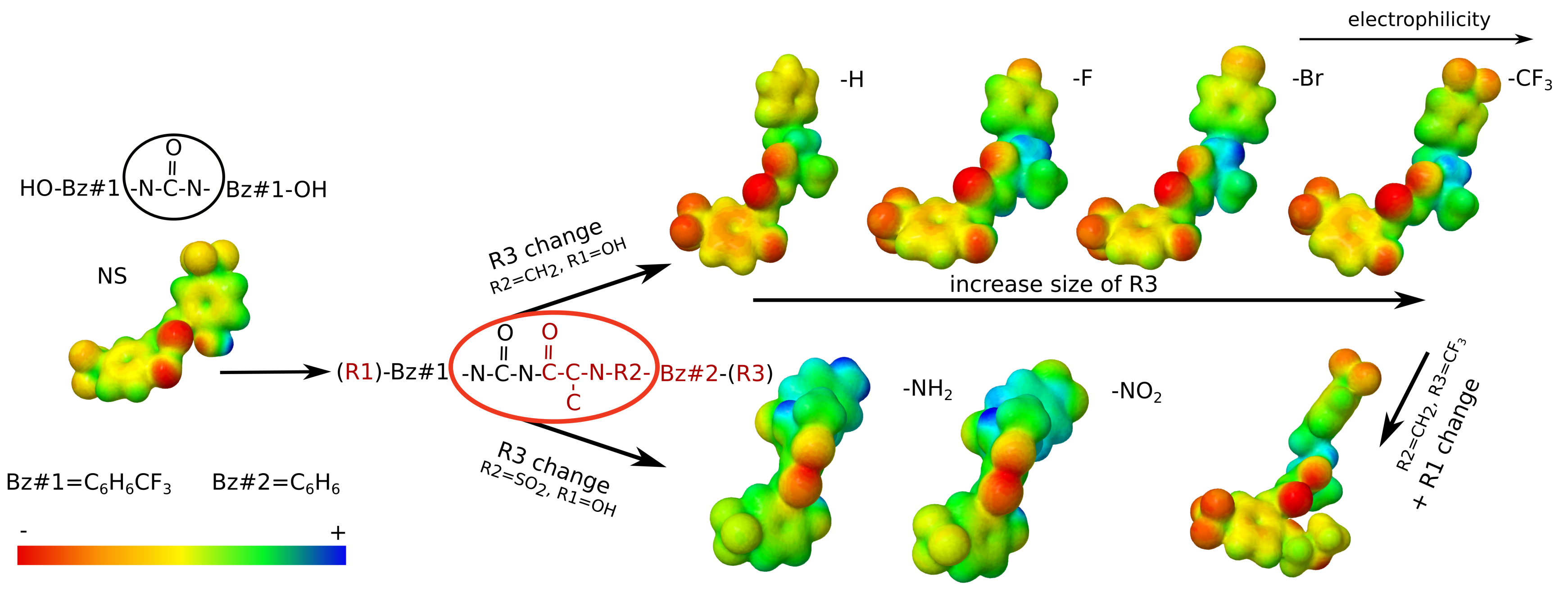

Figure S11. Comparison of electrostatic potential mapped to electron density (Isosurface contour 0.01) for NS1643 and its derivatives. The building blocks added to have the NS1643-derivatives are colored in red in accordance to the nomenclature defined in the text. The peptide-like linker is shown inside the red oval while the original moiety in the black one. Only thermodynamically favored conformers are shown and in the case of group **a** and **b** drugs, protonated drugs where considered.

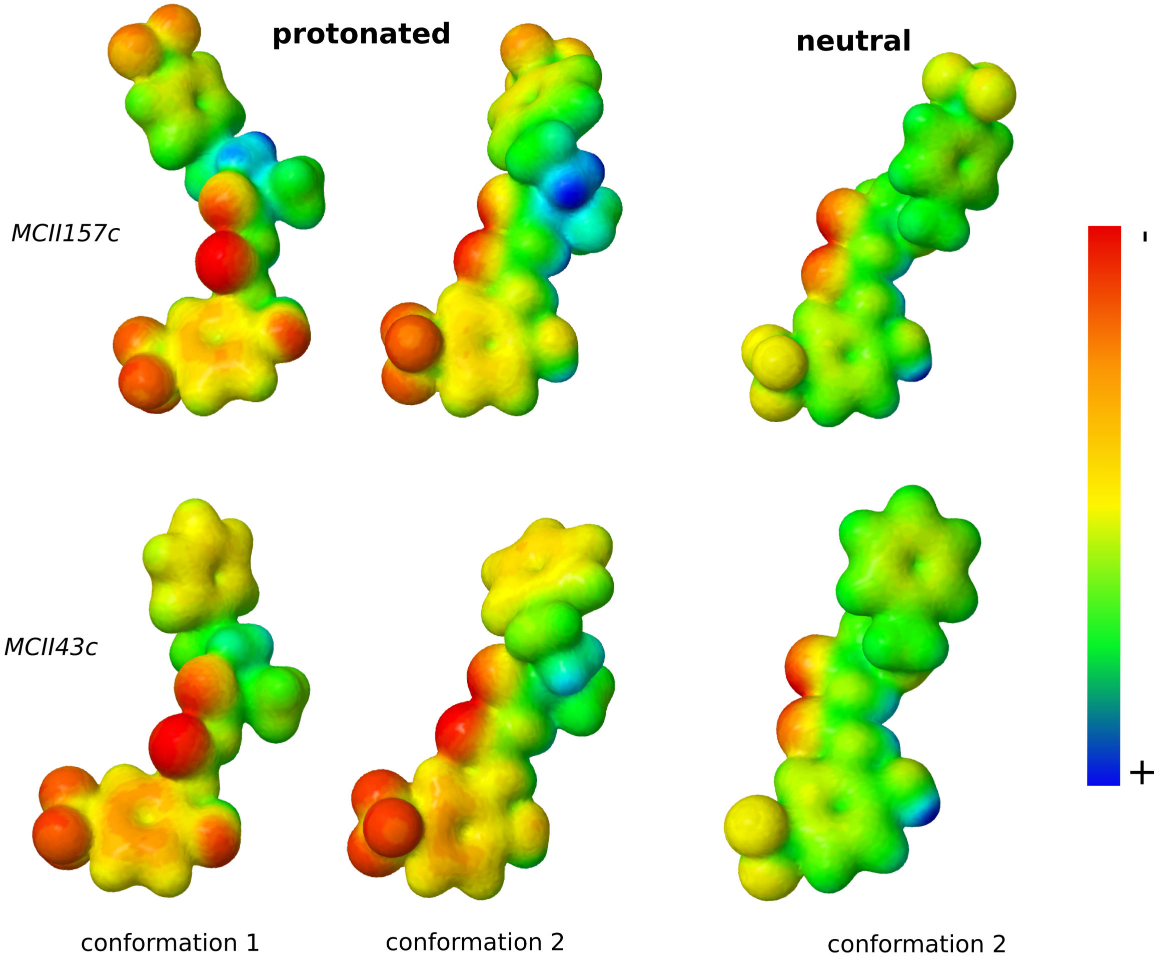

Figure S12. Comparison of electrostatic potential mapped to electron density (Isosurface contour 0.01) for the different conformers (1 and 2) and different protonation states for MC-II-157c and MC-II-43c drugs. In the case of neutral drug only the most favorable conformation is shown.

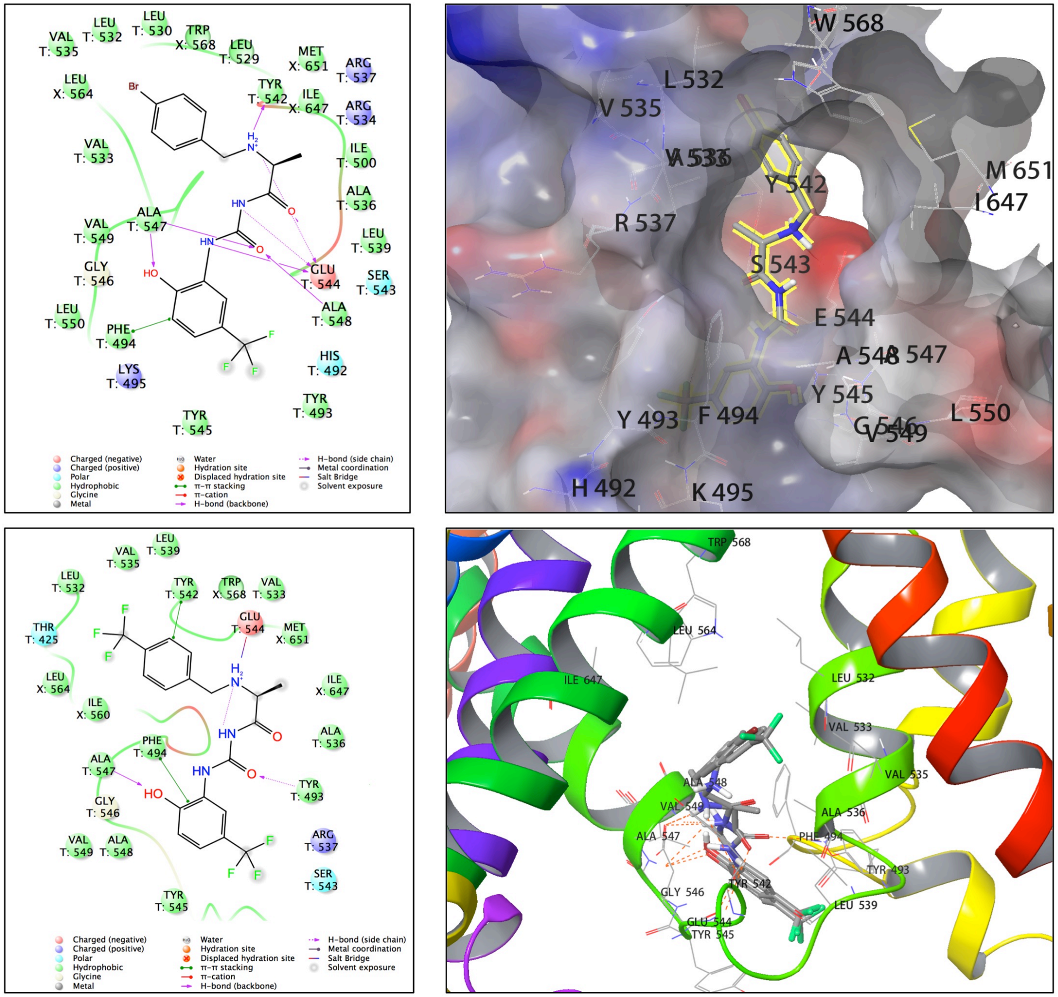

Figure S13. Top docking pose of MC-II-159c at the S4-S5 domain of the receptor. Its 2D ligand interactions diagram (left-top panel) is compared with MC-II-157c. (left-bottom panel). While surface representation of docked pose of MC-II-159c molecule is shown at the right-top panel, superimposition of docked conformations of high affinity two molecules MC-II-157c and MC-II-159c represented at right-bottom panel.

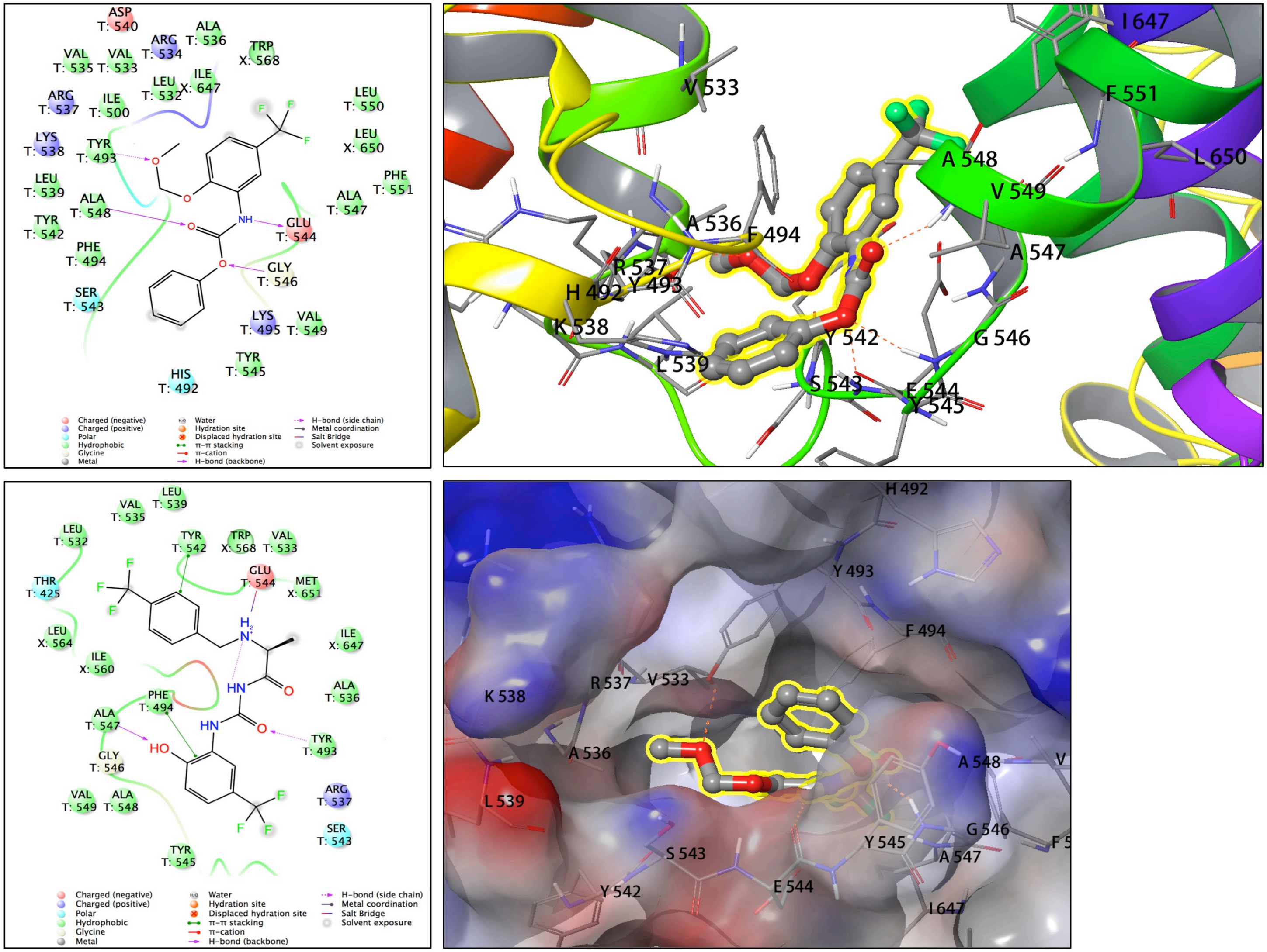

Figure S14. Top docking pose of one of the low affinity compounds (MC-I-167b) is shown at the S4-S5 domain of the receptor (top-right panel). 2D ligand interactions diagram (top-left panel) is compared with one of the high affinity compounds (MC-II-157c) (bottom-left panel). Surface representation of docked pose (bottom-right panel) is also shown at the figure.

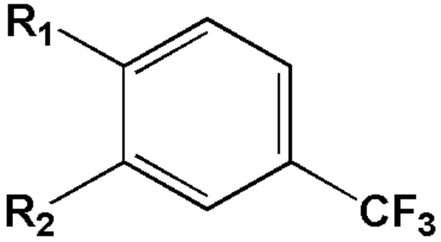

	R1	R2
MC-I-163b	-Cl	-CO-(NCH3)C6H6
MC-I-165b	-H	-CO-(NCH3)C6H5
MC-I-167b	-OCH2OCH3	-NH-CO2-C6H5

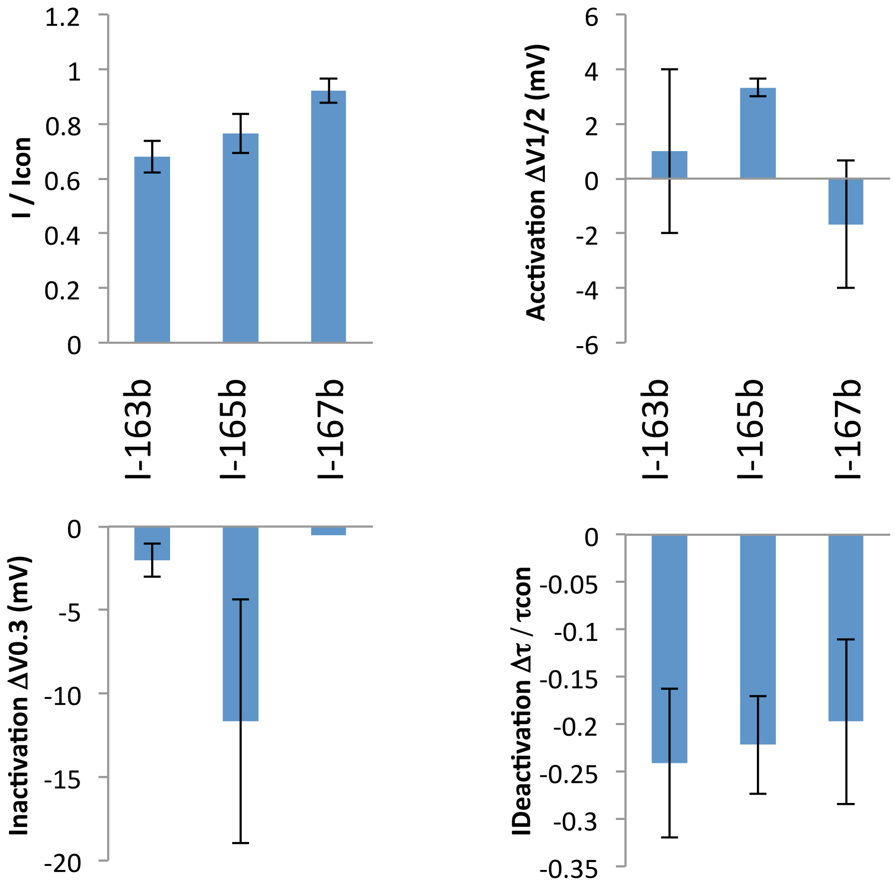

Figure S15. Electrophysiologic responses of MC-I-163b, MC-I-165b, MC-I-167b. Panel A shows structures of drugs, panel B (top) shows the magnitude of the tail current relative to baseline. Panel C (top) shows the shift in the voltage-dependence of activation and Panel B (bottom) shows the shift in the voltage-dependent of inactivation. Panel C (bottom) shows the deactivation time constants relative to base lines.

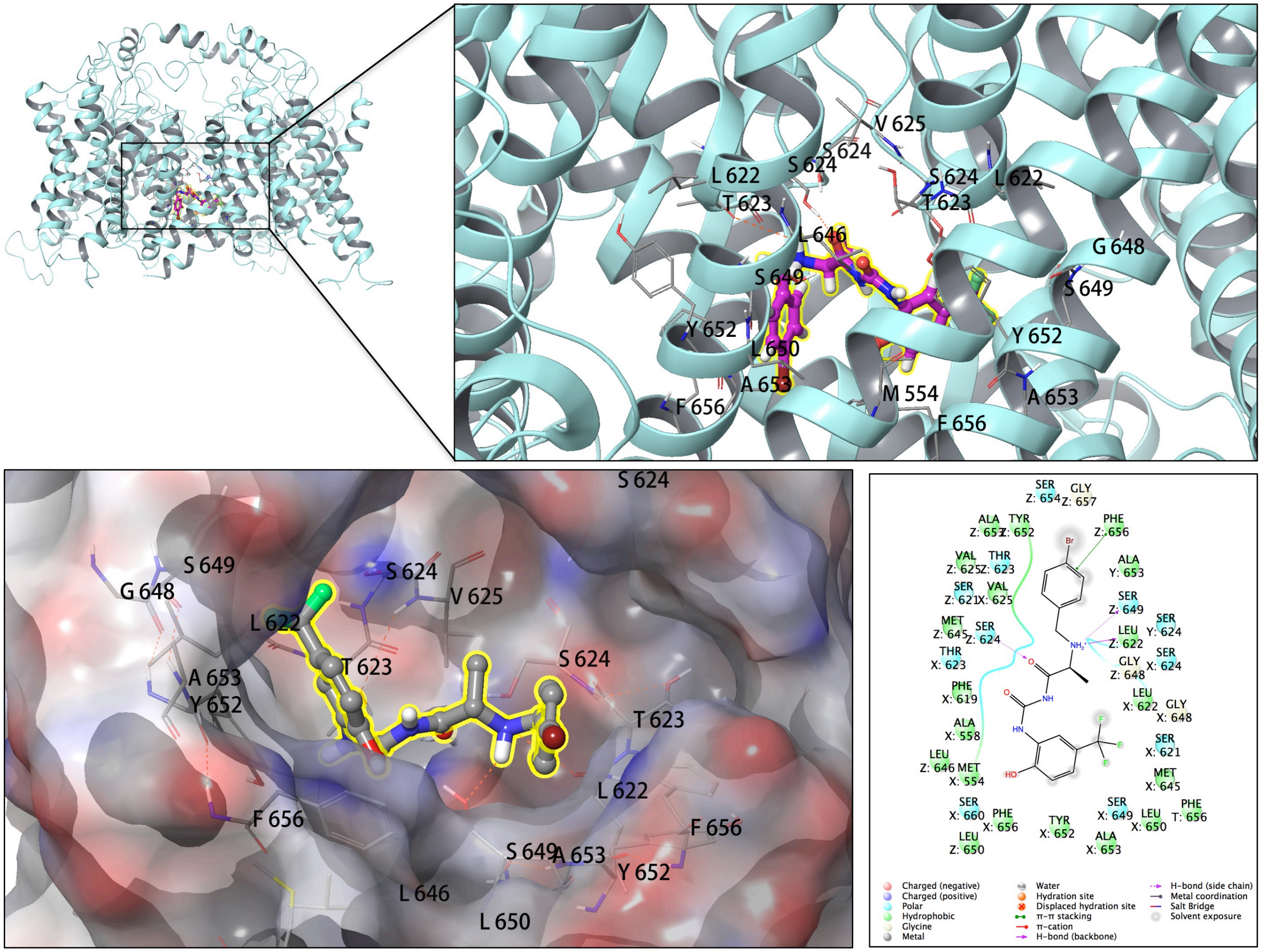

Figure S16. Top docking pose of one of the high affinity compounds (MC-II-159c) at the pore domain of the receptor. 2D ligand interactions diagram (right-bottom panel) and surface representation of docked pose (left-bottom panel) are also shown at the figure.
