# Supplementary material for: Structure Driven Design of Novel Human Ether-A-Go-Go-Related-Gene Channel (hERG1) Activators
Source: PLoS One. 2014 Sep 5;9(9):e105553. doi: 10.1371/journal.pone.0105553 (PMC4156305; doi:10.1371/journal.pone.0105553)
Supplement: File S2 — Tables S1 to S5 disclosing development of pharmacophore model, QM computations and details of ZINC database search. (PDF) [file pone.0105553.s002.pdf]

Table S1. Statistical parameter values for best 4 to 7 sites models using 24 compounds (NS1643 and its MC- derivatives).

4 sites: DDDR.164 (866 hypothesis)

PLS	SD	r^2	F	P	Stability	RMSE	q^2	Pearson-R	Ref. Ligand
1	0.31	0.86	72.8	$1.94*10^{-6}$	0.82	0.42	0.37	0.73	MC-II-157c
2	0.23	0.93	72.9	$4.49*10^{-7}$	0.74	0.43	0.37	0.72	MC-II-157c
3	0.17	0.96	90.3	$1.52*10^{-7}$	0.57	0.43	0.35	0.68	MC-II-157c

5 sites: AADHR.4 (554 hypothesis)

PLS	SD	r^2	F	P	Stability	RMSE	q^2	R-Pearson	Ref. Ligand
1	0.43	0.83	79.4	$1.33*10^{-7}$	0.97	0.40	0.44	0.89	MC-II-157c
2	0.24	0.95	142.1	$1.78*10^{-10}$	0.88	0.11	0.96	0.98	MC-II-157c
3	0.16	0.98	228.5	$4.01*10^{-12}$	0.80	0.18	0.89	0.94	MC-II-157c

6 sites: AADDDR.129 (489 hypothesis)

PLS	SD	r^2	F	P	Stability	RMSE	q^2	R-Pearson	Ref. Ligand
1	0.36	0.85	82.0	$3.14*10^{-7}$	0.87	0.34	0.60	0.90	MC-II-159c
2	0.31	0.91	62.2	$2.21*10^{-7}$	0.86	0.34	0.61	0.91	MC-II-159c
3	0.22	0.96	86.0	$2.22*10^{-8}$	0.68	0.32	0.64	0.84	MC-II-159c

7 sites: AADDDHR.3674 (283 hypothesis)

PLS	SD	r^2	F	P	Stability	RMSE	q^2	R-Pearson	Ref. Ligand
1	0.48	0.79	59.8	$8.60*10^{-7}$	0.92	0.51	0.22	0.78	MC-II-159c
2	0.45	0.82	34.7	$2.38*10^{-6}$	0.91	0.53	0.16	0.81	MC-II-159c
3	0.34	0.91	44.5	$2.10*10^{-7}$	0.73	0.39	0.56	0.86	MC-II-159c

Table S2. Electronic structure calculations for the different drugs at the B3LYP/6-31G** level in water (PCM)*.

Molecule	-R3	Protonation (N*)	Conformations	ΔE (kcal/mol)	Dipole Moment ($e*a_0$) Total	Dipole Moment ($e*a_0$) Component along **b**
**NS1643**		neutral	***a***<br>*b*	-3.09	3.92<br>2.56	0.08<br>1.62
**R1=-OH and R2=CH_2**						
MC-II-43c	-H	neutral	1, ***2***	+7.13	5.38	0.03
		protonated	***1***<br>2	-3.27	9.44<br>9.70	2.08<br>1.56
MC-II-159c	-Br	neutral	1, ***2***	+6.90	5.32	0.37
		protonated	***1***<br>2	-2.67	7.61<br>8.75	1.97<br>2.83
MC-II-157c	-CF_3	neutral	1, ***2***	+6.60	5.33	0.48
		protonated	***1***<br>2	-3.20	8.24<br>8.79	3.18<br>2.96
MC-II-163c	-F	neutral	1,***2***	+6.97	5.34	0.21
		protonated	***1***<br>2	-3.21	8.01<br>8.89	2.52<br>2.29
**R1=-OCH_2OCH_3 and R2=CH_2**						
MC-II-155b	-Br	neutral	1, ***2***	+4.95	5.18	0.35
		protonated	1<br>***2***	+3.19	6.44<br>6.75	1.98<br>1.81
MC-II-153b	-CF_3	neutral	1, ***2***	+7.46	5.18	0.17
		protonated	***1***<br>2	-2.93	6.91<br>8.59	2.45<br>3.06
MC-II-161b	-F	neutral	***2***	-	5.23	0.44
		protonated	***1***<br>2	-3.14	8.17<br>9.20	3.64<br>2.82
**R1=-OH and R2=-SO_2**						
MC-I-159b	-NH_2	neutral	1<br>***2***	+2.66	6.47<br>6.54	1.84<br>2.53
MC-I-169b	-NO_2	neutral	1<br>***2***	+2.68	3.58<br>3.06	0.23<br>0.41

* Dipole moments are shown in $e*a_0$ units (1 D = 0.393430307 ea_0 (atomic units)). **b** is the versor perpendicular to the plane defined by the peptide atoms (see Figure 6). The thermodynamically favored conformation is underlined in italic bold.

Table S3. Compounds from ZINC databank used in PHASE pharmacophore modeling. (Compounds **30** and **36** were used in test set)

Compound No	Name	2D structure	Docking Score (kcal/mol)	Predicted Activity pKi	Activity Calculated pKi	Number of Conformers
**25**	ZINC36744082	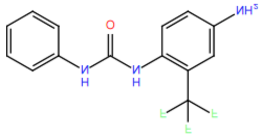	-11.86	8.64	8.65	5
**26**	ZINC03136944	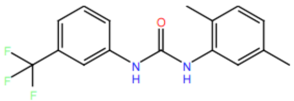	-11.56	8.42	8.03	11
**27**	ZINC16349697	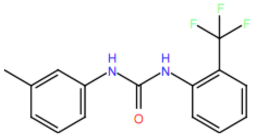	-9.96	7.26	7.40	9
**28**	ZINC02167791	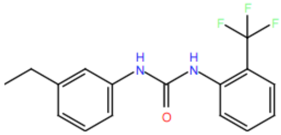	-9.83	7.16	7.29	9
**29**	ZINC51354173	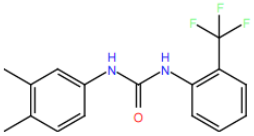	-9.73	7.09	7.36	7
**30**	ZINC51354202	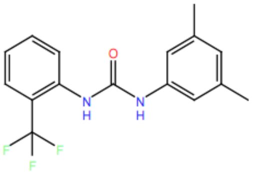	-9.67	7.04	7.42	5
**31**	ZINC01752675	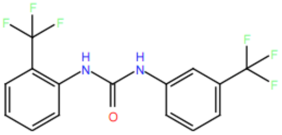	-9.55	6.96	6.83	14
**32**	ZINC01590170	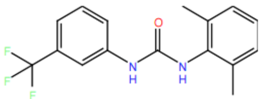	-9.50	6.92	6.20	9

**33**	ZINC51354212	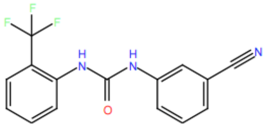	-9.43	6.87	6.83	8
**34**	ZINC03086031	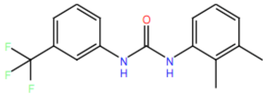	-9.43	6.87	6.52	10
**35**	ZINC02165490	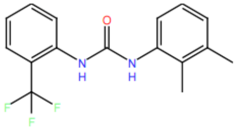	-9.14	6.66	6.60	11
**36**	ZINC16282573	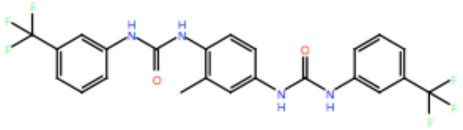	-8.73	6.36	6.52	34

Table S4. Statistical parameter values for best 5 sites models using 36 compounds (NS1643 and its synthesized MC- derivatives and 12 NS1643 derivatives from ZINC databank (extended data set)).

5 sites: DDDHR.2817 (814 hypothesis)

PLS	SD	r^2	F	P	Stability	RMSE	q^2	R-Pearson	Ref. Ligand
1	0.41	0.80	102.6	$2.44*10^{-10}$	0.51	0.33	0.60	0.84	MC-II-159c
2	0.30	0.90	105.3	$1.31*10^{-12}$	0.43	0.44	0.65	0.86	MC-II-159c
3	0.20	0.96	176.1	$5.28*10^{-16}$	0.31	0.31	0.50	0.80	MC-II-159c

Table S5. Experimental available pKa data in water for secondary amines, amides and N-methylbenzenesulfonamides.

	Substrate (BH^+)	pKa (BH^+= $B+H^+$)	[HB^+]/[B] (at pH=7)
Secondary amines[1]	$(CH_3)_2N^+H_2$	10.7	5.01 x10^3
	i-$Pr_2N^+H_2$	11.5	3.16x10^4
Amides[1]	$CH_3C(O)N^+H_2R$	-0.5	3.16x10^{-8}
N-methylbenzenesulfonamides[2]	a (prot. on O)[3]	-3.5	3.16x10^{-11}
	b (prot on N)[3]	-6.0	1.0x10^{-13}

[1]Advanced Organic Chem., 3rd Ed., J. March (1985), Unpublished results of W. P. Jencks.
[2]Moreira et al, *Belstein J. Org. Chem*. **2011**, 7, 1732-1738. Doi:10.3762/bjoc.7203.

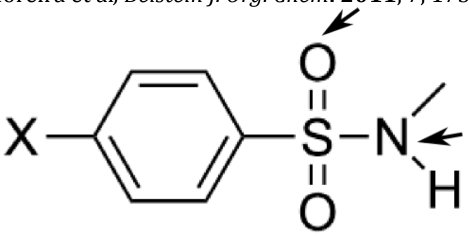

a: X=MeO
b: X=NO_2

[3]A possibility that should not be discarded is the existence of a tautomeric equilibrium between the N- and O- protonated structures, the latter having a greater relevance for a sulfonamides with X electron-donor groups.
