# Supplementary material for: Structure Driven Design of Novel Human Ether-A-Go-Go-Related-Gene Channel (hERG1) Activators
Source: PLoS One. 2014 Sep 5;9(9):e105553. doi: 10.1371/journal.pone.0105553 (PMC4156305; doi:10.1371/journal.pone.0105553)
Supplement: Methods S1 — Detailed description of computational and experimental methods used in the paper. (PDF) [file pone.0105553.s003.pdf]

SUPPLEMENTARY MATERIALS
Structure Driven Design of Novel Human Ether-a-Go-Go-Related-Gene Channel (hERG1) Activators

Jiqing Guo,[†] Serdar Durdagi,[‡,§] Mohamed Changalov,[#] Laura L. Perissinotti,[‡] Jason M. Hargreaves,[#] Thomas G. Back,[#,*] Sergei Y Noskov[‡,*] Henry J Duff[†,*]

[†]Libin Cardiovascular Institute of Alberta, University of Calgary, Calgary, Alberta, Canada; [‡]Institute for Biocomplexity and Informatics, Department of Biological Sciences, University of Calgary, Calgary, Alberta, Canada; [§]Department of Biophysics, Faculty of Medicine, Bahcesehir University, Istanbul, Turkey; [#]Department of Chemistry, University of Calgary, Calgary, Alberta, Canada.

I. Methods- Supplementary Material

QM Calculations

To provide better understanding of ionization states and stable conformers for synthesized molecules we performed conformational scan and geometry optimization with quantum chemical computations. All calculations were carried out with the Gaussian09 package[1] and full optimization of the geometries of all species at the B3LYP level, and with the 6-31G** basis set, without symmetry constraints. Solvent effects were modeled using the polarized continuum model scheme (PCM) implemented in Gaussian09, which performs a reaction field calculation using the integral equation formalism model (IEFPCM). Each stationary point was characterized by performing a normal mode analysis. Electron density 3D isosurface plots were generated using the Cubegen utility included in the Gaussian09 package; cubes were generated from the formatted checkpoint file. Electrostatic potentials were mapped to the electron density surface for the most feasible conformations for each protonation state. Visual Molecular Dynamics (VMD)[2] and open source PyMol[3] were used to visualize electron densities and electrostatic potentials. Dipole moments were extracted from the Gaussian09 output for each optimized structure and were reoriented according to a common set of axes found for all molecules, their directions and magnitudes were compared throughout the different series of compounds. Dipole moments were visualized using VMD Tcl script. In order to further compare the dipole moments for the different compounds, the component along the versor (b) perpendicular to the plane defined by the six atoms N-C-O-N-C-O, in the polyamide moiety (common structure element present in all drugs) was calculated. (Figure 3) POV-Ray3.6 and open Source Vector Graphics editor (Inkscape) were used for making the final figures.

Generation of 3D QSAR and Docking Models

All of the NS1643 derivatives were prepared and minimized (i.e., using determination of protonation states at pH 7 followed by energy minimization (with a 0.001 kcal/mol Å convergence threshold). Next compounds were docked at the central cavity of hERG, the structures that carried low-docking score together with low predicted pIC_{50} and low Fitness Score profiles were selected. Then, from the comparison between calculated and predicted pK_i values, statistical results were derived to describe how efficient each 3D-QSAR model is for predicting the pK_i values of NS1643 derivatives. While r^2, *standard deviation (SD)*, *F*, and *P* results were used to evaluate training set predictions; q^2, *RMSE*, and *Pearson-R* results were used to evaluate test set predictions. Finally these results were considered to rank the derived hundreds of pharmacophore hypotheses using highest r^2, q^2, and *Pearson-R* and lowest *SD*, *RMSE* and *P* values. Selected hypotheses from each different number of sites (4 to 7) are evaluated and represented in Table 2. From 4 to 7 points analyses, 5-points and 6-points universal pharmacophore models (AADHR.4 and AADDDR.129, respectively) showed very high r^2 and q^2 values and low *SD* and *RMSE* values, thus they were considered for further analyses. Calculated and predicted pK_i values of training and test set compounds are shown in Table 1. A very high correlation coefficient r^2 (i.e., 0.95 for 5-sites model; 0.91 for 6-sites model) observed between calculated and predicted pK_i values validate the reliability of models. The number of compounds used in PHASE pharmacophore modeling has been extended

from 24 to 36 by adding another 12 NS1643 homologs available from the ZINC database.[17] (NS1643 derivatives are selected with >80% structural similarity to NS1643, see Supplementary Material, Table S1) The protocol of pharmacophore modeling that applied for 24 compounds is repeated for extended data set (36 compounds). The optimal scores are derived with 5-sites pharmacophore model namely DDDHR.2817 using 817 hypotheses. Derived r^2 (0.90) and q^2 (0.65) statistical values for the model DDDHR.2817 are slightly smaller than corresponding 5-site model (AADHR.4) generated without ZINC database compounds. (Supplementary Material, Tables S1 and S2) Alignment of pharmacophore sites in the 5-sites model for active compounds and distances between each site are shown in the Supplementary Material, Figure S5. In Figure 2, results of contour map analysis of five-point pharmacophore model AADHR.4 on the functional chemical groups is represented with high-affinity (i.e., MC-II-157c) and low-affinity (i.e., MC-I-167b) compounds. While hydrophobic features of the ideal pharmacophore overlap nicely with the matching functionality in the high-affinity activator (i.e., green-colored hydrophobic feature favorable contours match perfectly with corresponding alkyl groups and aromatic rings), in the low-affinity-activator MC-I-167b purple colored hydrophobic features of unfavorable regions fit with the nonpolar parts of the compound.

These ligands were then docked to the S4S5-linker binding site identified to be important for hERG activation by small molecules. Developed compounds with high binding scores were used for further analysis and construction of 3D QSAR model for prospective development of hERG openers. (See supplementary materials, Figure S1-S3). Thei predicted pIC_{50} values for intra-cavitary blockade of hERG were evaluated with (i) the previously constructed pharmacophore model by our laboratory[4]; and (ii) Glide/XP docking scores. Compounds that have low predicted pIC_{50} values (i.e., less hERG blocking activity) and low Fitness Scores were selected for the docking simulations (~900 compounds). The details on the construction of 3D-QSAR Models are found in the Supplementary Materials-Methods section. For better interpretation of the results of electrophysiology studies on synthesized compounds, a 3D-QSAR study is also performed. Since the dataset for the tested compounds are not large enough yet (as well as not so diverse), we used calculated binding scores (from induced fit docking (IFD)/Glide XP scores at S4S5 linker site) of each compound and their 3D structures at the 3D-QSAR applications. Three approaches are considered: (a) QSAR is constructed only for synthesized and electrophysiology experiments applied for 24 compounds (b) in order to improve and cover more fragments, dataset extended to 36 compounds with available molecular structure databases (i.e., ZINC) with NS-like compounds. (iii) conformers of compounds used in (a) and (b) is considered with and without constrained to the top-docking poses for each compound used in the QSAR and their effects to the statistical results are studied. In order to see the effect of each fragment at the dataset and cross-validate the QSAR results, electrophysiology experiments applied compounds are subtracted from the training set one by one and their effect to the statistical results are also investigated. The 3D-QSAR study was carried out with the PHASE (v.3.0) program of the Schrodinger molecular modeling package. Prior to the QSAR application, all ligands were built and prepared and minimized with Maestro (v.9.2) Macromodel module. The PRCG method with a 0.001 kcal/mol Å convergence threshold is used for the geometry optimization studies. These structures were then incorporated into the

ligand preparation (LigPrep, v.2.2) module of the Schrodinger molecular modeling package. The protonation state for all ionizable groups is tested at neutral pH. A conformational search algorithm is performed with the Monte Carlo Multiple Minimum (MCMM) method using enhanced torsion sampling (2000 steps with 100 steps per every rotatable bond) for the derivation of conformers used in the training and test sets (see Table 1 and Figure 1). The energy window for saving the structures was set to 21.0 kJ/mol and 0.5 Å RMSD is used to eliminate redundant conformers. The number of conformers for each ligand used is shown at the supporting material Table S1, and Table 1 at the main text.

Docking scores (Glide/XP/see below) from Induced Fit Docking (kcal/mol) are converted to pKi values for the training and test set compounds (calculated pKi). The predicted (from QSAR) and the calculated pKi values are given at the Table 1. The specific interactions between a target and a ligand depend on a structural complementarity between functional groups presented in the ligand and coordinating residues from the binding pocket. PHASE classifies these chemical features as hydrogen bond acceptors (labeled as A), hydrogen bond donors (labeled as D), hydrophobic groups (labeled as H), negatively charged groups (labeled as N), positively charged groups (labeled as P) and aromatic rings (labeled as R). PHASE uses the most active compounds (high affinity) in the training set to build the pharmacophores. In order to define a common pharmacophore or hypothesis, PHASE employs an analysis of *k*-point pharmacophores derived from the conformational sets of active compounds and then identifies all spatial arrangements with pharmacophore features that are shared by those compounds. In this study, we set following pKi thresholds for the selection of active and inactive compounds: $pKi < 4.5$, and $pKi > 7$ is considered as inactive and active ligands, respectively. Thus, a common pharmacophore must match a minimum required number of active ligands, which was set as 5 in this study. All parameters used during the 3D QSAR model construction had default values except for the maximum number of partial least square (PLS) factors (i.e., 3), the 3D QSAR model type (i.e., atom-based) and the percent of ligand assigned to the training and test sets (i.e., 70% and 30%, respectively). PLS factor 2 was used for the construction of contour plots. While statistical results together with number of hypothesis with different combinations of site numbers are detailed in Table 2, contour plots for the selected predicted high-affinity and low-affinity compounds have been shown at the Figure 2.

Selection Criteria for Generated Ligand Databases and available Ligand Libraries

Structures from ligand databases and libraries (>500,000) were downloaded. LigPrep module of Schrodinger was used for the ligand preparation at neutral pH 7 and geometry optimization is performed. The structures were then docked at the S4S5-linker site (Grid map is prepared with 14 Å cubic box in this region. This site was proposed to be functionally important by several independent studies. All ligands are docked by Glide high throughput virtual screening (HTVS) method using E544 ligand as interaction constrain. Ligands unable to form interaction with E544 were ignored and ligands that have higher (absolute values) binding scores than -7.00 kcal/mole were collected. The selected structures are then re-docked using Glide extra precision (XP) method at the same binding pocket with same binding constraints. The collected structures from Glide/XP method is then docked to the central cavity of hERG1 and compounds that have

binding score more than -7 kcal/mole at the central cavity are ignored and rest are collected for further studies. The beneficial modifications suggested by QSAR analysis of NS-based derivatives were used to construct a library of compounds aimed at highlighting different binding pockets in hERG1 channel.

Approach to Molecular Docking

(i) Glide/IFD. Docking studies were performed using two different approaches for the definition of binding sites. First, a blind docking was performed and common binding sites were focused with high precision grid mapping. Second, Sitemap module of the Schrodinger molecular modeling package (was set minimum site points per reported site as 15, using fine-grid option and crop site maps at 10 Å from nearest site point) was used to define and score the site maps at the target and top-scored binding sites were used at the definition of binding pockets. Glide/XP v.5.0 combined with Prime module of Schrodinger was used for the IFD calculations. The scoring was first carried out using Schrödinger's discretized version of the ChemScore empirical scoring function. Only a small number of the best-refined poses was passed and both docked poses and surrounding amino acids with radial cavity of 5.0 Å minimized (molecular mechanics, OPLS-AA). Finally, the minimized poses were re-scored using Schrödinger' s proprietary GlideScore scoring function. GlideScore is based on ChemScore, but includes a steric-clash term and adds other rewards and penalties. The choice of best-docked structure for each ligand is made using a model energy score (Emodel) that combines the energy grid score, the binding affinity predicted by GlideScore, and (for flexible docking) the internal strain energy for the model potential used to direct the conformational-search algorithm. Default charges were used for all ligands (OPLS-2005), the funnel width was increased by adjusting the energy window to 5.0, the CvdW cutoff was 10.0, and the clustering criteria was set to 0.75. XP was used for all dockings. The Prime algorithm implemented in Glide was used to account for the flexibility of the receptor. A complete description of the IFD protocol can be found in the IFD users' s manual. *(ii) GOLD.* GOLD program was used to dock same ligands to cross-validate performance of GLIDE/IFD. The GOLD program (version 5.1) was used with two default docking scores (the GOLD Fitness and ChemScore). The dockings produced using the GoldScore function were re-scored and re-ranked with the optimized ChemScore function. Partial flexibility of the receptor was gained with selected amino acids residues at the selected binding site. The side chains of these amino acids were selected as flexible rotamers. The default genetic algorithm parameters were used. Default GOLD charges were used for each ligand (based on Sybyl atom types). The maximum number of runs was set to 100 for each docking simulation. *(iii) Clustering of docking results.* Clustering of docking poses was performed using the software Contactos. This program calculates the similarities between docked poses of protein ligands. The similarity matrices produced by Contactos is then used to cluster the docking results using an external clustering tool, MCL. Contactos has successfully been used in combination with MCL to cluster docking results from IFD and GOLD runs. Contactos generates a descriptor of the protein-ligand contacts for each docked pose. The program goes through each ligand atom – protein atom pair. If the atoms are within the cut-off distance of each other, they are defined as a 'contact' (The cutoff used was 3.5 A). Once the descriptors are generated for each docked pose in the input, Contactos calculates an all-against-all matrix of similarities which is then used as input to do the

clustering. Visualization and analysis of docking results were done using Maestro Molecular Modeling Interface software, open source Pymol and Hermes interface program. POV-Ray3.6 and open Source Vector Graphics editor (Inkscape) were used for making the final figures.

Short Summary of QM Computations

The structure, dipole moment and electron density was analyzed for the following groups of drugs and then compared.

(i) **R1**=OH, **R2**=CH_2, **R3**=H, CF_3, Br, and F

When drug is fully protonated two stable conformers can co-exist at room temperature. The structural difference between two conformers is mainly due to the position of the aromatic rings which rotates around the bonds shown in the Figure 6 (black arrows). The relatively low energy difference between these conformers suggests a low barrier for this rotation and indicates that either orientation may occur at room temperature. These two conformers have similar dipole moment, in module and direction. In the case of neutral drug, the energy difference between conformers is higher and only one conformer (2) was considered in the neutral state computations. The dipole moment changes considerably for this structure in module (lower) and direction, the component along the vector b is almost zero (see Figures 6 and Supplementary Material, Figure S7).

(ii) **R1**=OCH2OCH3, **R2**=CH2, **R3**=CF3, Br, and F

In this group of drugs, the hydroxyl group is changed by an ether moiety which is bulkier and more hydrophobic than hydroxyl group. The structural changes and energetics between the two conformers differ considerably with **R3** subtitutions, as in the previous case. For the protonated drugs, the trifluoromethyl benzene ring is more twisted when bearing the ether than in the hydroxyl case, while the rotation of the other bond seems to be more restricted, in fact it doesn't occur for the -Br and -F. Importantly, the dipole moment differs significantly for conformations **1** and **2**, in terms of module and direction (see Figure S7 to S11 at the supplementary material). In the case of neutral drugs, here again only one conformation (**2**) was considered; dipole moment is less sensitive to the **R3** substituent as in the previous case and very similar to the hydroxyl series. In general, as in the previous case, the distribution of species will be determined by pKa, but what is clear in this group is that structural changes between conformers when the drug is protonated affects the dipole moment magnitude and direction in a different way than the OH series, and that might affect the interaction in the binding site modifying the activity of these drugs (see supplementary material, Figure S8 and Table 3).

(iii) **1**=OH, **R2**=SO_2, **R3**=NH_2 and NO_2

The presence of the sulfonamide group makes these drugs more rigid in terms of rotation around the C-C bond (see Figure 6). Here one of the sulfonamide's oxygen is establishing an intra molecular hydrogen bond with one of the amide atoms, hindering that rotation. The movement of the trifluoromethyl benzene ring is similar to the first group of drugs in (i). Both two conformers, are feasible, the dipole moment for both drugs changes it direction and magnitude (see Figure 6 and supplementary material Figure S9). The direction is within the limits found for the hydroxyl's series but the magnitude is much lower, especially in the case of R3=NO_2.

Electrophysiology Protocol:
The pipette solution contained the following (at mM concentration): KCl 10, K-aspartate 110, $MgCl_2$ 5, Na_2ATP 5, EGTA 10, HEPES 5, and $CaCl_2$ 1. The solution was adjusted to pH 7.2 with KOH. The extracellular solution contained the following (at mM concentration): NaCl 140, KCl 5.4, $CaCl_2$ 1, $MgCl_2$ 1, HEPES 5, and glucose 5.5. The solution was adjusted to pH 7.4 with NaOH. For I_{Kr} measurements, the whole cell configuration of the patch clamp method was used. The series resistance was less than 7 MΩ. The data were sampled at 1 kHz. The holding potential was -80 mV. The activation of hERG tail currents was induced by depolarization to a range of potentials from +50 to -100 mV in 10 mV steps for 1 second. This was followed by repolarization to -100 mV for 1 second to record the tail currents. If the tail current did not completely deactivate by -100 mV, a -120 mV 1 second pre-pulse was applied to completely close the hERG channels before subsequent depolarization. All of the tail currents reported in this manuscript represent dofetilide-sensitive currents. The inactivation of hERG was measured by a previously reported triple-pulse protocol[5, 6]. The holding potential was -80 mV. The hERG channel was first depolarized to +50 mV for 1 second. Recovery from inactivation was achieved using a short pulse (a 10 ms hyperpolarization) to -120 mV, followed by the application of test pulses from -100 to 0 mV for 1 second in 10 mV steps. A voltage of -100 mV was selected because it was on the flat part of the inactivation-voltage relationship. The methods employed are identical to ones previously reported.

II. Supplementary Tables Labels

Table S1. Statistical parameter values for best 4 to 7 sites models using 24 compounds (NS1643 and its MC- derivatives).

Table S2. Electronic structure calculations for the different drugs at the B3LYP/6-31G** level in water (PCM). The thermodynamically favored conformation is underlined in italic bold. Dipole moments are shown in $e*a_0$ units (1 debye = 0.393430307 ea_0 (atomic units)). **b** is the versor perpendicular to the plane defined by the peptide atoms (see Figure 6).

Table S3. Compounds from ZINC databank used in PHASE pharmacophore modeling.

Table S4. Statistical parameter values for best 5 sites models using 36 compounds (NS1643 and its synthesized MC- derivatives and 12 NS1643 derivatives from ZINC databank).

Table S5. Experimental available pKa data in water for secondary amines, amides and N-methylbenzenesulfonamides.

III. Supplementary Figures

Figure S1. Construction of NS- derivatives. (top) Around 4000 small-compound database is used for the production of new NS derivatives. (bottom) 10 enumeration sites are used to generate new derivatives. Together with different binding combinations of the fragments, total number of derivatives reached to ~40000.

Figure S2. Derived ligands are screened at our previously reported hERG pharmacophore model (Reference 6 at the main text) for predicting their hERG blocking profiles. Compounds that have low predicted pIC_{50} values as well as low Fitness scores (low hERG blocking activities) were selected (highlighted region) for next step (Docking).

Figure S3. Combination of Ligand- and Receptor-based scoring. Selected compounds from pharmacophore modeling (see Figure S2) are docked at the central cavity of hERG1, the structures that carry low-docking score together with low pIC_{50} and low Fitness score profile are selected for ligand docking at the S4S5-linker binding site.

Figure S4. (top) Selected ligand from combination of Ligand- and Receptor-based screening. Figure shows it's predicted pIC_{50} value and Fitness score at hERG pore domain by pharmacophore model; and it's central cavity as well as S4S5 linker site (i.e., E544 site) docking by Glide/XP. (bottom) Ligand interaction diagram of selected ligand at the S4S5 site.

Figure S5. Superimposition of the pharmacophore sites at active compounds. Distances between each site have been shown at right part of Figure.

Figure S6. Alignment of 5-pharmacophore sites with active ligands (left) and selected ligands from hit-screening (right).

Figure S7. Optimized structures and dipole moment for drugs bearing R1=OH, R2=CH2 and R3=H, Br, CF3 and F; in both protonation states. The two conformers found for the protonated state, 1 and 2 are superimposed and shown in cyan and green respectively. Dipole moment together with the corresponding optimized drug structure are shown in different colors for all superimposed drugs for protonated (a) and neutral (b) states.

Figure S8. Optimized structures and dipole moment for drugs bearing R1=OCH2OCH3, R2=CH2 and R3=Br, CF3 and F; in both protonation states. The two conformers found for the protonated state, 1 and 2 are superimposed and shown in cyan and green respectively. Dipole moment together with the corresponding optimized drug structure are shown in different colors for all superimposed drugs for protonated (a) and neutral (b) states. Comparison of dipole moments for all conformations for the hydroxyl and ether series of compounds (c).

Figure S9. Optimized structures and dipole moment for drugs bearing R1=OH, R2=SO2 and R3=NH2 and NO2. The two conformers found, 1 and 2 are superimposed and shown

in cyan and green respectively. Dipole moment together with the corresponding optimized structure are shown in different colors (a). Comparison of dipole moments for all conformations for the hydroxyl and sulfonamide series of compounds (b).

Figure S10. Structure, electron density (Isosurface contour is 0.05) and dipole moment for NS and one drug selected from each group (**a**, **b**, **c**). Both thermodynamically feasible conformers found for the drugs are superimposed and shown in cyan and green respectively. For drugs MC-I-155b and MC-I-159c only the protonated state is shown. In the cases of MC-II-155b and MC-I-159b dipole moment and structures are superimposed and compare to MC-II-159c.

Figure S11. Comparison of electrostatic potential mapped to electron density (Isosurface contour 0.01) for NS1643 and its derivatives. The building blocks added to have the NS1643-derivatives are colored in red in accordance to the nomenclature defined in the text. The peptide-like linker is shown inside the red oval while the original moiety in the black one. Only thermodynamically favored conformers are shown and in the case of group **a** and **b** drugs, protonated drugs where considered.

Figure S12. Comparison of electrostatic potential mapped to electron density (Isosurface contour 0.01) for the different conformers (1 and 2) and different protonation states for MC-II-157c and MC-II-43c drugs. In the case of neutral drug only the most favorable conformation is shown.

Figure S13. Top docking pose of MC-II-159c at the S4-S5 domain of the receptor. Its 2D ligand interactions diagram (left-top panel) is compared with MC-II-157c. (left-bottom panel). While surface representation of docked pose of MC-II-159c molecule is shown at the right-top panel, superimposition of docked conformations of high affinity two molecules MC-II-157c and MC-II-159c represented at right-bottom panel.

Figure S14. Top docking pose of one of the low affinity compounds (MC-I-167b) is shown at the S4-S5 domain of the receptor (top-right panel). 2D ligand interactions diagram (top-left panel) is compared with one of the high affinity compounds (MC-II-157c) (bottom-left panel). Surface representation of docked pose (bottom-right panel) is also shown at the figure.

Figure S15. Electrophysiologic responses of MC-I-163b, MC-I-165b, MC-I-167b. Panel A shows structures of drugs, panel B (top) shows the magnitude of the tail current relative to baseline. Panel C (top) shows the shift in the voltage-dependence of activation and Panel B (bottom) shows the shift in the voltage-dependent of inactivation. Panel C (bottom) shows the deactivation time constants relative to base lines.

Figure S16. Top docking pose of one of the high affinity compounds (MC-II-159c) at the pore domain of the receptor. 2D ligand interactions diagram (right-bottom panel) and surface representation of docked pose (left-bottom panel) are also shown at the figure.

References:

1. Gaussian, I. Wallingford CT, 2009.
2. Humphrey, W.; Dalke, A.; Schulten, K., VMD: Visual molecular dynamics. *J. Mol. Graph. Model.* **1996**, 14, 33-38.
3. Shah, R. R., Drug-induced prolongation of the QT interval: why the regulatory concern? *Fundamental & Clinical Pharmacology* **2002**, 16, 119-124.
4. Durdagi, S.; Duff, H. J.; Noskov, S. Y., Combined Receptor and Ligand-Based Approach to the Universal Pharmacophore Model Development for Studies of Drug Blockade to the hERG1 Pore Domain. *J. Chem. Inf. Model.* **2011**, 51, 463-474.
5. Smith, P. L.; Baukrowitz, T.; Yellen, G., The inward rectification mechanism of the HERG cardiac potassium channel. *Nature* **1996**, 379, 833-836.
6. Spector, P. S.; Curran, M. E.; Zou, A. R.; Sanguinetti, M. C., Fast inactivation causes rectification of the I-Kr channel. *J. Gen. Physiol.* **1996**, 107, 611-619.
