# Supplementary material for: Structure Driven Design of Novel Human Ether-A-Go-Go-Related-Gene Channel (hERG1) Activators
Source: PLoS One. 2014 Sep 5;9(9):e105553. doi: 10.1371/journal.pone.0105553 (PMC4156305; doi:10.1371/journal.pone.0105553)

IV. Supplementary Files for Synthesis of NS1643 Analogues

N-(*N,N*-Dibenzyl-*L*-alanyl)-*N*'-[(2-(methoxymethoxy)-5-(trifluoromethyl)phenyl] urea (17, MC-II-67-b)

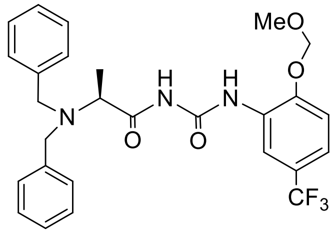

An oven-dried flask was charged with powdered 4Å molecular sieves (93 mg), anhydrous DMF (1 mL) and anhydrous LiOH (9.8 mg, 0.41 mmol) and the mixture was stirred under argon. After 20 min, amine **16** (55 mg, 0.16 mmol) was added and the reaction mixture was stirred for another 45 min, followed by the addition of benzyl bromide (56 mg, 0.33 mmol) and stirring for another 8 h. The reaction mixture was then diluted with dichloromethane and the solution was filtered and evaporated under reduced pressure. The residue was purified by flash chromatography on silica gel (2% methanol-dichloromethane) to afford 35 mg (42%) of the dibenzyl derivative **17** as an oil; IR (film) 3313, 3226, 1712, 1606 cm^{-1}; ^{1}H NMR (400 MHz, $CDCl_3$) δ 10.80 (s, 1 H), 9.61 (s, 1 H), 8.67 (s, 1 H), 7.43-7.25 (m, 11 H), 7.20 (d, *J* = 8.6 Hz, 1 H), 5.33 (s, 2 H), 3.82 (d, *J* = 13.3 Hz, 2 H), 3.54-3.47 (m, 1 H), 3.52 (s, 3 H), 3.44 (d, *J* = 13.1 Hz, 2 H), 1.39 (d, *J* = 6.7 Hz, 3 H); ^{13}C NMR (101 MHz, $CDCl_3$) δ 175.7, 150.1, 148.4, 137.8, 129.2, 129.1, 128.4, 128.1, 124.5 (q, *J* = 32.7 Hz), 124.4 (q, *J* = 271.7 Hz), 121.0 (q, *J* = 4.0 Hz), 117.3 (q, *J* = 3.9 Hz), 113.6, 95.1, 58.1, 56.7, 54.8, 6.7; HRMS (ESI) calcd for $C_{27}H_{29}F_3N_3O_4$ $(M+H)^+$: 516.2105; found: 516.2097.

***N*-(*N*-4-Fluorobenzyl-*L*-alanyl)-*N*'-[2-(methoxymethoxy)-5-(trifluoromethyl)phenyl] urea (8, MC-II-161-b)**

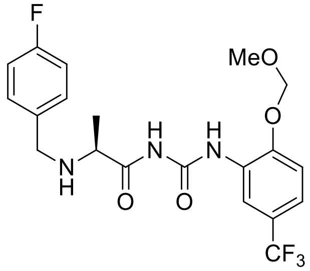

Amine **16** (157 mg, 0.468 mmol) and ethyldiisopropylamine (242 mg, 1.87 mmol) were stirred for 10 min in dry acetonitrile (2.7 mL) at room temperature. 4-Fluorobenzyl bromide (88.5 mg, 0.468 mmol) was added, the reaction mixture was stirred at room temperature for 1 h and then was heated at 55 °C overnight. The solvent was evaporated and the residue was purified by flash chromatography on silica gel (5% methanol-dichloromethane) to afford 104 mg (50%) of urea **8** as a white solid; mp 87-88 °C; IR (film) 3236, 3137, 1695 cm^{-1}; ^1H NMR (400 MHz, $CDCl_3$) δ 10.96 (s, 1 H), 9.67 (br s, 1 H), 8.65 (d, *J* = 2.1 Hz, 1 H), 7.34-7.27 (m, 3 H), 7.23 (d, *J* = 8.5 Hz. 1 H), 7.04 (crude t, *J* = 8.7 Hz, 2 H), 5.36 (s, 2 H), 3.82 (d, *J* = 13.2 Hz, 1 H), 3.77 (d, *J* = 13.1 Hz, 1 H), 3.56 (s, 3 H), 3.41 (q, *J* = 7.1 Hz, 1 H), 1.79 (br s, 1 H), 1.44 (d, *J* = 7.0 Hz, 3 H); ^{13}C NMR (101 MHz, $CDCl_3$) δ 176.7, 162.3 (d, *J* = 246.1 Hz), 150.2, 148.3, 134.2, 129.8 (d, *J* = 8.1 Hz), 128.3, 124.2 (q, *J* = 271.7 Hz), 124.2 (q, *J* = 32.7 Hz), 120.9 (q, *J* = 4.0 Hz), 117.1 (q, *J* = 3.9 Hz), 115.6 (d, *J* = 21.5 Hz), 113.4, 95.0, 57.9, 56.5, 52.0, 19.2; HRMS (ESI) calcd for $C_{20}H_{22}F_4N_3O_4$ $(M+H)^+$: 444.1541; found: 444.1547. Anal. calcd for $C_{20}H_{21}F_4N_3O_4$: C, 54.18; H, 4.77; N, 9.48; found: C, 54.29; H, 4.60; N, 9.33.

N-(N-4-Fluorobenzyl-L-alanyl)-N'-[2-hydroxy-5-(trifluoromethyl)phenyl] urea, trifluoro-acetate salt (3, MC-II-163-c)

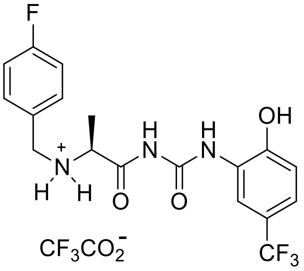

Trifluoroacetic acid (3.08 mL) was added dropwise to urea **8** (45 mg, 0.10 mmol) in dry dichloromethane (1.8 mL). The reaction mixture was stirred at room temperature overnight, the solvent was evaporated to dryness and the residue was triturated with hexanes-ethyl acetate (8:2:) to afford 30 mg (58%) of phenol **3** as the trifluoroacetate salt, obtained as a white solid; mp 170-171 °C; IR (film) 3402, 3246, 3117, 3004, 1702, 1672 cm^{-1}; ^{1}H NMR (600 MHz, CD_3OD) δ 8.48 (d, *J* = 2.2 Hz), 7.57-7.55 (m, 2 H), 7.26-7.21 (m, 3 H), 6.98 (d, *J* = 8.4 Hz), 4.27 (s, 2 H), 4.16-4.05 (m 1 H), 1.66 (d, J = 7.1 Hz); ^{13}C NMR (151 MHz, CD_3OD) δ 172.2, 165.1 (d, *J* = 248.5), 163.1 (q, *J* = 34.1 Hz), 151.6, 151.0, 133.7 (d, *J* = 8.7 Hz), 126.1 (q, *J* = 269.0 Hz), 128.4 (d, *J* = 3.3 Hz), 127.8, 122.8 (q, *J* = 31.9 Hz), 122.5 (q, *J* = 4.0 Hz), 118.1 (q, *J* = 3.9 Hz), 117.3 (d, *J* = 22.0 Hz), 115.4, 57.4, 50.4, 16.6; ^{19}F NMR (376 MHz, CD_3OD) δ -62.7, -76.6, -113.0; HRMS (ESI) calcd for $C_{18}H_{18}F_4N_3O_3$ $(M+H)^+$: 400.1279; found: 400.1283.

N-(N-4-Bromobenzyl-L-alanyl)-N'-[2-(methoxymethoxy)-5-(trifluoromethyl)phenyl] urea (9, MC-II-155-b)

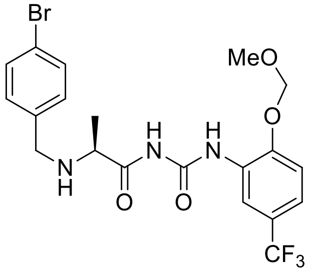

Amine **16** (200 mg, 0.596 mmol) and ethyldiisopropylamine (270 mg, 2.09 mmol) were stirred in dry acetonitrile (3.5 mL) for 10 min at room temperature. 4-Bromobenzyl bromide (149 mg, 0.596 mmol) was then added and the reaction mixture was stirred at room temperature for 1 h. The mixture was then stirred overnight at 55 °C. The solvent was evaporated and the residue was purified by flash chromatography on silica gel (5% methanol-dichloromethane) to afford 130 mg (43%) of urea **9** as a white solid; mp 101-102 °C; IR (film) 3323, 3236. 3127, 1689 cm^{-1}; ^{1}H NMR (400 MHz, $CDCl_3$) δ 10.95 (s, 1 H), 9.61 (br s, 1 H), 8.65 (d, *J* = 2.2 Hz, 1 H), 7.49 (dt, *J* = 8.3, 2.2 Hz, 2 H), 7.32 (dd, *J* = 8.4, 2.1 Hz, 1 H), 7.25-7.20 (m, 3 H), 5.36 (s, 2 H), 3.81 (d, *J* = 13.4, Hz, 1 H), 3.75 (d, *J* = 13.4, Hz, 1 H), 3.56 (s, 3 H), 3.45-3.77 (m, 1 H), 1.74 (br s, 1 H), 1.44 (d, *J* = 7.0 Hz, 3 H); ^{13}C NMR (101 MHz, $CDCl_3$) δ 176.7, 150.4, 148.3, 137.3, 131.9, 130.0, 128.2, 124.3 (q, *J* = 32.7 Hz), 124.2 (q, *J* = 271.6 Hz), 120.9 (q, *J* = 4.0 Hz), 117.1 (q, *J* = 3.9 Hz), 113.4, 95.0, 57.9, 56.5, 52.0, 19.1; HRMS (ESI) calcd for $C_{20}H_{22}BrF_3N_3O_4$ $(M+H)^+$:

504.0740; found: 504.0730. Anal. calcd for $C_{20}H_{21}BrF_3N_3O_4$: C, 47.63; H, 4.20; N, 8.33; found: C, 48.05; H, 3.83; N, 8.13.

_N_-(_N_-4-Bromobenzyl-_L_-alanyl)-N'-[2-hydroxy-5-(trifluoromethyl)phenyl] urea, trifluoroacetate salt (4, MC-II-159-c)

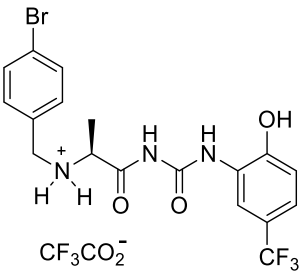

Trifluoroacetic acid (3.46 mL) was added dropwise to urea **9** (75 mg, 0.15 mmol) in dry dichloromethane (2.7 mL). The reaction mixture was stirred at room temperature for two days, the solvent was evaporated to dryness and the residue was triturated with a mixture of hexanes-ethyl acetate-dichloromethane (8:2:1) to afford 50 mg (59%) of phenol **4** as the trifluoroacetate salt, obtained as a white solid; mp 128-129 °C; IR (film) 3412, 3243, 3120, 1699, 1675 cm^{-1}; ^{1}H NMR (600 MHz, CD_3OD) δ 8.48 (s 1 H), 7.63 (crude d, J = 8.1 Hz, 2 H), 7.44 (crude d, J = 8.1 Hz, 2 H), 7.25 (d, J = 8.5 Hz, 1 H), 6.98 (d, J = 8.4 Hz, 1 H), 4.23 (s, 2 H), 4.16-4.02 (m, 1 H), 1.65 (d, J = 7.0 Hz, 3 H); ^{13}C NMR (151 MHz, CD_3OD) δ 170.8, 161.7 (q, J = 33.4 Hz), 150.0, 149.4, 132.0, 131.8, 130.2, 126.2, 124.5 (q, J = 270.3 Hz), 123.6, 121.2 (q, J = 32.4 Hz), 120.9 (q, J = 4.1 Hz), 116.5 (q, J = 4.5 Hz), 116.8 (q, J = 292.8 Hz), 113.8, 56.0, 49.0, 15.1; ^{19}F NMR (376 MHz, CD_3OD) δ -62.6, -76.6; HRMS (ESI) calcd for $C_{18}H_{18}BrF_3N_3O_3$ $(M+H)^+$: 460.0478; found:460.0477. Anal. calcd for $C_{20}H_{18}BrF_6N_3O_5$: C, 41.83; H, 3.16; N, 7.32; found: C, 42.01; H, 3.14; N, 6.90.

_N_-[_N_-4-(Trifluoromethylbenzyl)-_L_-alanyl]-_N_'-[2-(methoxymethoxy)-5-(trifluoromethyl)-phenyl] urea (10, MC-II-153-b)

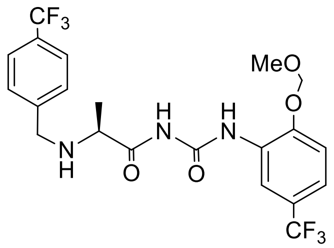

Amine **16** (200 mg, 0.596 mmol) and ethyldiisopropylamine (270 mg, 2.09 mmol) were dissolved in dry acetonitrile (3.5 mL). After 10 min, 4-(trifluoromethyl)benzyl bromide (143 mg, 0.598 mmol) was added dropwise. The reaction mixture was stirred at room temperature for 1 h and then at 55°C in an oil bath overnight. The solvent was evaporated and the residue was purified by flash chromatography on silica gel (5% methanol-dichloromethane) to afford 150 mg (51%) of urea **10** as a white solid; mp 95-96°C; IR (film) 3233, 3130, 1699 cm^{-1}; ^{1}H NMR (600

MHz, $CDCl_3$) δ 11.01 (s, 1 H), 9.84, (s, 1 H), 8.63 (d, *J* = 2.2 Hz, 1 H), 7.57 (crude d, *J* = 8.0 Hz, 2 H), 7.45 (crude d, *J* = 7.7 Hz, 2 H), 7.31 (dd, *J* = 8.6, 0.9 Hz, 1 H), 7.22 (d, *J* = 8.6 Hz, 1 H), 5.36 (d, *J* = 6.8 Hz, 1 H), 5.34 (d, *J* = 6.7 Hz, 1 H), 3.91 (d, *J* = 13.7 Hz, 1 H), 3.84 (d, *J* = 13.7 Hz, 1 H), 3.55 (s, 3 H), 3.43 (q, *J* = 7.0 Hz, 1 H), 1.80 (br s, 1 H), 1.46 (d, *J* = 7.1 Hz, 3 H); ^{13}C NMR (151 MHz, $CDCl_3$) δ 177.0, 150.8, 148.5, 142.8, 130.0 (q, *J* = 32.4 Hz), 128.6, 128.3, 125.8 (q, *J* = 3.8 Hz), 124.4 (q, *J* = 271.6 Hz), 124.3 (q, *J* = 32.7 Hz), 124.2 (q, *J* = 272.0 Hz), 121.1 (q, *J* = 4.0 Hz), 117.2 (q, *J* = 3.9 Hz), 113.6, 95.2, 58.3, 56.7, 52.2, 19.3; HRMS (ESI) calcd for $C_{21}H_{22}F_6N_3O_4$ $(M+H)^+$: 494.1509; found: 494.1506.

_N_-[_N_-(4-Trifluoromethylbenzyl)-_L_-alanyl]-N'-[2-hydroxy-5-(trifluoromethyl)phenyl] urea, trifluoroacetate salt (5, MC-II-157-c)

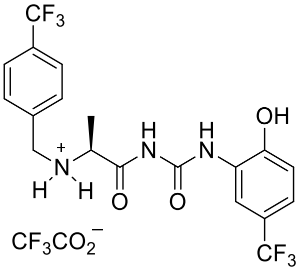

Trifluoroacetic acid (2.36 mL) was added dropwise to urea **10** (50 mg, 0.10 mmol) in dry dichloromethane (1.84 mL). The reaction mixture was stirred at room temperature overnight, the solvent was evaporated and the product was triturated with hexanes-ethyl acetate (8:2) to provide 31 mg (54%) of urea **5** as the trifluoroacetate salt, obtained as white solid; mp 112-113 °C; IR (film) 3399, 3249, 3117, 1702, 1672 cm^{-1}; ^{1}H NMR (400 MHz, CD_3OD) δ 8.48 (d, *J* = 2.2 Hz, 1 H), 7.79 (crude d, *J* = 8.1 Hz, 2 H), 7.73 (crude d, *J* = 8.1 Hz, 2 H), 7.25 (dd, *J* = 8.4, 2.2 Hz, 1 H), 6.99 (d, *J* = 8.4 Hz, 1 H), 4.35 (s, 2 H), 4.20-4.07 (m, 1 H), 1.66 (d, *J* = 7.1 Hz, 3 H); ^{13}C NMR (151 MHz, CD_3OD) δ 172.5, 163.2 (q, *J* = 34.2 Hz), 151.6, 151.0, 137.1, 132.9 (q, *J* = 32.6 Hz), 132.2, 132.1, 127.8, 127.3 (q, *J* = 3.8 Hz), 126.1 (q, *J* = 270.1 Hz), 125.5 (q, *J* = 271.43 Hz), 122.8 (q, *J* = 32.6 Hz), 122.5 (q, *J* = 4.0 Hz), 118.1 (q, *J* = 3.9 Hz), 115.4, 57.8, 50.6, 16.7; ^{19}F NMR (376 MHz, CD_3OD) δ -61.7, -63.1, -75.6; HRMS (ESI) calcd for $C_{19}H_{18}F_6N_3O_3$ $(M+H)^+$: 450.1247; found: 450.1245. Anal. calcd for: $C_{21}H_{18}F_9N_3O_5$: C, 44.77; H, 3.22; N, 7.46; found: C, 44.50; H, 3.11; N, 7.29.

_N_-(_N_-Benzoyl-_L_-alanyl)-_N_'-[2-(methoxymethoxy)-5-(trifluoromethyl)phenyl] urea (11, MC-II-57-c)

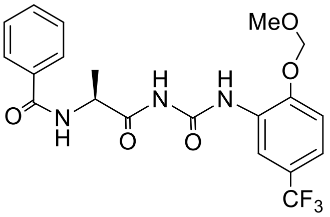

An oven-dried flask containing amine **16** (120 mg, 0.358 mmol) and pyridine (0.25 mL) was immersed in an ice bath and benzoyl chloride (56 mg, 0.40 mmol) was added. The reaction mixture was warmed to room temperature and stirred under argon overnight. The solvent was then evaporated and the residue was purified by flash chromatography on silica gel (5% methanol-dichloromethane). The product was recrystallized from ethyl acetate-hexanes to afford 117 mg (73%) of urea **11** as a white solid; mp 180-182 °C; IR (film) 3263, 3143, 1702, 1642 cm^{-1}; ^{1}H NMR (400 MHz, $CDCl_3$) δ 10.99 (s, 1 H), 10.09 (s, 1 H), 8.59 (s, 1 H), 7.81 (d, *J* = 7.7 Hz, 2 H), 7.49 (t, *J* = 7.5 Hz, 1 H), 7.40-7.22 (m, 4 H), 6.86 (d, *J* = 6.9 Hz, 1 H), 5.35 (s, 2 H), 4.94-4.83 (m, 1 H), 3.54 (s, 3 H), 1.66 (d, *J* = 7.1 Hz, 3 H); ^{13}C NMR (101 MHz, $CDCl_3$) δ 174.8, 167.7, 151.7, 148.8, 133.1, 132.3, 128.8, 128.0, 127.4, 124.4 (q, *J* = 271.5 Hz), 124.3 (q, *J* = 32.7 Hz), 121.5 (q, *J* = 4.3 Hz), 117.5 (q, *J* = 3.9 Hz), 113.8, 95.2, 56.8, 51.0, 17.8; HRMS (ESI) calcd for $C_{20}H_{21}F_3N_3O_5$ $(M+H)^+$: 440.1428; found: 440.1419. Anal. calcd for $C_{20}H_{20}F_3N_3O_5$: C, 54.67; H, 4.59; N, 9.56; found: C, 54.61; H, 4.33; N, 9.39.

_N_-(_N_-Benzoyl-_L_-alanyl)-_N_'-[2-hydroxy-5-(trifluoromethyl)phenyl] urea (6, MC-II-61-c)

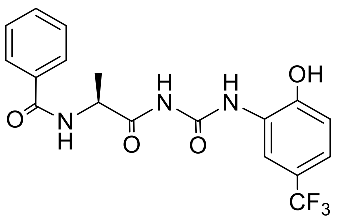

Urea **11** (45 mg, 0.10 mmol) was dissolved in dichloromethane (1.8 mL). Trifluoroacetic acid (1.15 mL) was added dropwise and the reaction was stirred at room temperature overnight. The solvent was then evaporated and the residue was purified by flash chromatography on silica gel (5% methanol-dichloromethane). The product was recrystallized from ethyl acetate-hexanes, producing a white crystalline solid. The solid was washed with hexanes and dried in vacuo to afford 36.5 mg (90%) of phenol **6**; mp 192-194 °C; IR (film) 3452, 3246, 3133, 1702, 1642 cm^{-1}; ^{1}H NMR (400 MHz, CD_3OD) δ 8.72 (d, *J* = 6.3 Hz, 1 H), 8.48 (s, 1 H), 7.91 (crude d, *J* = 7.7 Hz, 2 H), 7.58-7.44 (m, 3 H), 7.21 (d, *J* = 8.6 Hz, 1 H), 6.95 (d, *J* = 8.3 Hz, 1 H), 4.68-4.59 (m, 1 H), 1.55 (d, *J* = 7.2 Hz, 3 H); ^{13}C NMR (101 MHz, CD_3OD) δ 176.7, 170.6, 152.7, 151.0, 135.0, 133.1, 129.7, 128.8, 128.1, 126.2 (q, *J* = 270.1 Hz), 122.7 (q, *J* = 32.4 Hz), 122.1 (q, *J* = 3.8 Hz), 118.0 (q, *J* = 4.1 Hz), 115.3, 52.0, 17.5; HRMS (ESI) calcd for $C_{18}H_{17}F_3N_3O_4$ $(M+H)^+$: 396.1166; found: 396.1161.

N-(N-4-Nitrobenzoyl)-L-alanyl)-N'-[2-(methoxymethoxy)-5-(trifluoromethyl)phenyl] urea (12, MC-II-59-c)

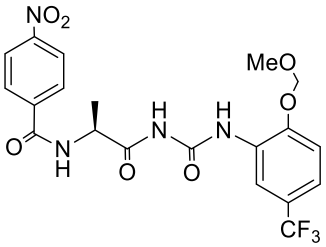

4-Nitrobenzoyl chloride (85 mg, 0.46 mmol) was added to amine **16** (139 mg, 0.415 mmol) and pyridine (0.25 mL) while cooling at 0 °C. The reaction mixture was warmed to room temperature and stirred under argon overnight. Volatile material was then evaporated and the residue was purified by flash chromatography on silica gel (5% methanol-dichloromethane). Recrystallization from ethyl acetate-hexanes afforded 151 mg (75%) of urea **12** as a yellow crystalline solid; mp 190-193 °C; IR (film) 3412, 3273, 3123, 1705, 1649 cm^{-1}; ^{1}H NMR (400 MHz, $CDCl_3$) δ 10.98 (s, 1 H), 10.32 (s, 1 H), 8.49 (s, 1 H), 8.13 (d, *J* = 8.3 Hz, 2 H), 7.91 (d, *J* = 8.3 Hz, 2 H), 7.37 (d, *J* = 8.8 Hz, 1 H), 7.28 (s, 1 H), 6.88 (d, *J* = 7.0 Hz, 1 H), 5.37 (d, *J* = 6.7 Hz, 1 H), 5.33 (d, *J* = 6.8 Hz, 1 H), 4.87-4.78 (m, 1 H), 3.55 (s, 3 H), 1.70 (d, *J* = 7.2 Hz, 3 H); ^{13}C NMR (101 MHz, $CDCl_3$) δ 174.2, 165.6, 152.0, 149.7, 148.7, 138.6, 128.3, 127.3, 124.1 (q, *J* = 271.7 Hz), 123.9 (q, *J* = 33.8 Hz), 123.7, 121.7 (q, *J* = 4.0 Hz), 117.21 (q, *J* = 4.4 Hz), 113.8, 95.1, 56.7, 51.3, 17.7; HRMS (ESI) calcd for $C_{20}H_{20}F_3N_4O_7$ $(M+H)^+$: 485.1279; found: 485.1277.

N-(N-4-Nitrobenzoyl-L-alanyl)-N'-[2-hydroxy-5-(trifluoromethyl)phenyl] urea (7, MC-II-63-c)

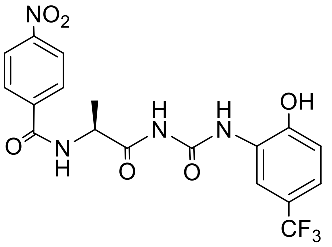

Urea **12** (45 mg, 0.093 mmol) was dissolved in a mixture of dichloromethane (1.7 mL) and trifluoroacetic acid (1.04 mL), and the reaction was stirred at room temperature overnight. The solvent was evaporated and the residue was purified by flash chromatography on silica gel (5% methanol-dichloromethane). The product was recrystallized from ethyl acetate-hexanes and the solid was triturated with hexanes dried in vacuo to afford 37 mg (91%) of phenol **7** as a yellow crystalline solid; mp 209-211 °C; IR (film) 3422, 3253, 3146, 1699, 1642 cm^{-1}; ^{1}H NMR (400 MHz, CD_3OD) δ 8.48 (s, 1 H), 8.33 (crude d, *J* = 8.8 Hz, 2 H), 8.10 (crude d, *J* = 8.8 Hz, 2 H), 7.21 (dd, *J* = 8.6, 2.3 Hz, 1 H), 6.94 (d, *J* = 8.5 Hz, 1 H), 4.65 (q, *J* = 7.2 Hz, 1 H), 1.57 (d, *J* = 7.2 Hz, 3 H); ^{13}C NMR (101 MHz, CD_3OD) δ 174.8, 167.0, 151.1, 149.8, 149.4, 139.2, 128.7, 126.5, 124.6 (q, *J* = 270.4 Hz), 123.2, 121.1 (q, *J* = 32.4 Hz), 120.6 (q, *J* = 4.2 Hz), 116.4 (q, *J* =

4.0 Hz), 113.7, 50.5, 15.9; HRMS (ESI) calcd for $C_{18}H_{16}F_3N_4O_6$ $(M+H)^+$: 441.1017; found: 441.1025.

_N_-(_N_-_p_-Nitrobenzenesulfonyl-_L_-alanyl)-_N_'-[2-(methoxymethoxy)-5(trifluoromethyl)phenyl] urea (15, MC-I-155-b)

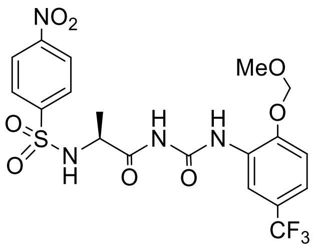

Amine **16** (545 mg, 1.62 mmol) was dissolved in dry 1,2-dichloroethane (9 mL) and dry pyridine (0.17 mL). 4-Nitrobenzenesulfonyl chloride (360 mg, 1.62 mmol) was added and the mixture was heated in an oil bath at 80 °C for 2 h. The solvent was evaporated and the residue was purified by flash chromatography on silica gel (5% methanol-dichloromethane) or by precipitation with diethyl ether, to obtain 420 mg (50%) of urea **15** as light yellow solid; mp 183-184 °C; IR 3229, 1686 cm^{-1}; ^1H NMR (400 MHz, DMSO-d_6) δ 11.01 (s, 1 H), 10.63 (s, 1 H), 8.79 (br s, 1 H), 8.49 (s, 1 H), 8.36 (crude d, *J* = 8.6 Hz, 2 H), 8.02 (crude d, *J* = 8.6 Hz, 2 H), 7.41 (d, *J* = 8.8 Hz, 1 H), 7.30 (d, *J* = 8.7 Hz, 1 H), 5.34 (d, *J* = 6.6 Hz, 1 H), 5.30 (d, *J* = 6.7 Hz, 1 H), 4.21-4.09 (m, 1 H), 3.39 (s, 3 H), 1.23 (d, *J* = 6.8 Hz, 3 H); ^{13}C NMR (101 MHz, DMSO-d_6) δ 174.6, 150.7, 149.9, 148.3, 146.7, 128.7, 128.5, 124.8, 124.7 (q, *J* = 271.5 Hz), 122.5 (q, *J* = 32.2 Hz), 121.1 (q, *J* = 4.0 Hz), 115.8 (q, *J* = 4.3 Hz), 114.6, 95.2, 56.6, 52.6, 18.8; HRMS (ESI) calcd for $C_{19}H_{20}F_3N_4O_8S$ $(M+H)^+$: 521.09485; found: 521.09484 .

_N_-(_N_-_p_-Nitrobenzenesulfonyl-_L_-alanyl)-_N_'-[2-hydroxy-5-(trifluoromethyl)phenyl] urea (14, MC-I-169-b)

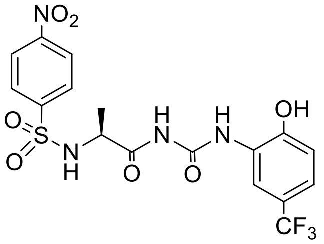

Urea **15** (45 mg, 0.086 mmol) was suspended in dry dichloromethane (1 mL). Trifluoroacetic acid (1.0 mL) was added dropwise and the mixture was stirred at room temperature overnight. The solvent was then evaporated and the residue was purified by flash chromatography on silica gel (5% methanol-dichloromethane) to afford 33 mg (80%) of phenol **14** as a white solid; mp 200-201 °C; IR 3226, 3107, 1705 cm^{-1}; ^1H NMR (400 MHz, DMSO-d_6) δ 11.01, 10.90, 10.49, 8.76 (d, *J* = 8.3 Hz, 1 H), 8.38 (s, 1 H), 8.35 (crude d, *J* = 8.6 Hz, 2 H), 8.02 (crude d, *J* = 8.6 Hz, 2 H), 7.26 (d, *J* = 8.4 Hz, 1 H), 7.00 (d, *J* = 8.4 Hz, 1 H), 4.16-4.06 (m, 1 H), 1.22 (d, *J* = 6.8 Hz, 3 H); ^{13}C NMR (101 MHz, DMSO-d_6) δ 173.8, 150.2, 149.5. 149.2, 146.1, 128.2, 126.4,

124.6 (q, *J* = 271.0 Hz), 124.4, 120.7 (q, *J* = 4.0 Hz), 119.5 (q, *J* = 31.9 Hz), 116.1 (q, *J* = 4.3 Hz), 114.4, 52.1, 18.3; HRMS (ESI) calcd for $C_{17}H_{16}F_3N_4O_7S$ $(M+H)^+$: 477.0686; found: 477.0684.

_N_-(_N_-_p_-Aminobenzenesulfonyl-_L_-alanyl)-_N_'-[2-hydroxy-5-(trifluoromethyl)phenyl] urea (13, MC-I-159-b)

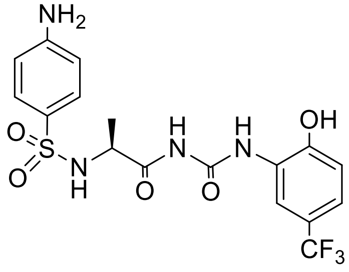

Phenol **14** (280 mg, 0.588 mmol) was dissolved in ethanol (8 mL) and 10% palladium on carbon (150 mg) was added. The mixture was hydrogenated overnight at room temperature under a slight positive pressure of hydrogen maintained by a balloon. The catalyst was filtered, the solvent was evaporated and the residue was purified by flash chromatography on silica gel (5% methanol-dichloromethane) to afford 175 mg (67%) of phenol **13** as white solid; mp 122-123 °C; IR 3481, 3386, 3249, 1708 cm^{-1}; ^1H NMR (300 MHz, DMSO-d_6) δ 11.07 (s, 1 H), 10.79 (s, 1 H), 10.67 (s, 1 H), 8.44 (d, *J* = 2.3 Hz, 1 H), 7.74 (d, *J* = 7.8 Hz, 1 H), 7.43 (crude d, *J* = 8.6 Hz, 2 H), 7.27 (dd, *J* = 8.5, 2.2 Hz, 1 H), 7.04 (d, *J* = 8.5 Hz, 1 H), 6.58 (crude d, *J* = 8.7 Hz, 2 H), 5.91 (s, 2 H), 3.96-3.84 (m, 1 H), 1.13 (d, *J* = 7.1 Hz, 3 H);); ^{13}C NMR (101 MHz, DMSO-d_6) δ 175.4, 153.1, 151.0, 149.8, 129.0, 127.1, 125.8, 125.1 (q, *J* = 270.9 Hz), 121.1 (q, *J* = 4.1 Hz), 119.9 (q, *J* = 32.0 Hz), 116.0 (q, *J* = 4.2 Hz), 114.9, 113.0, 52.3, 18.8; ^{19}F NMR (376 MHz, DMSO-d_6) δ -61.0; HRMS (ESI) calcd for $C_{17}H_{18}F_3N_4O_5S$ $(M+H)^+$: 447.0945; found: 447.0944.

^{1}H and ^{13}C NMR Spectra (CD_3OD) of 2 (MC-II-43-c)

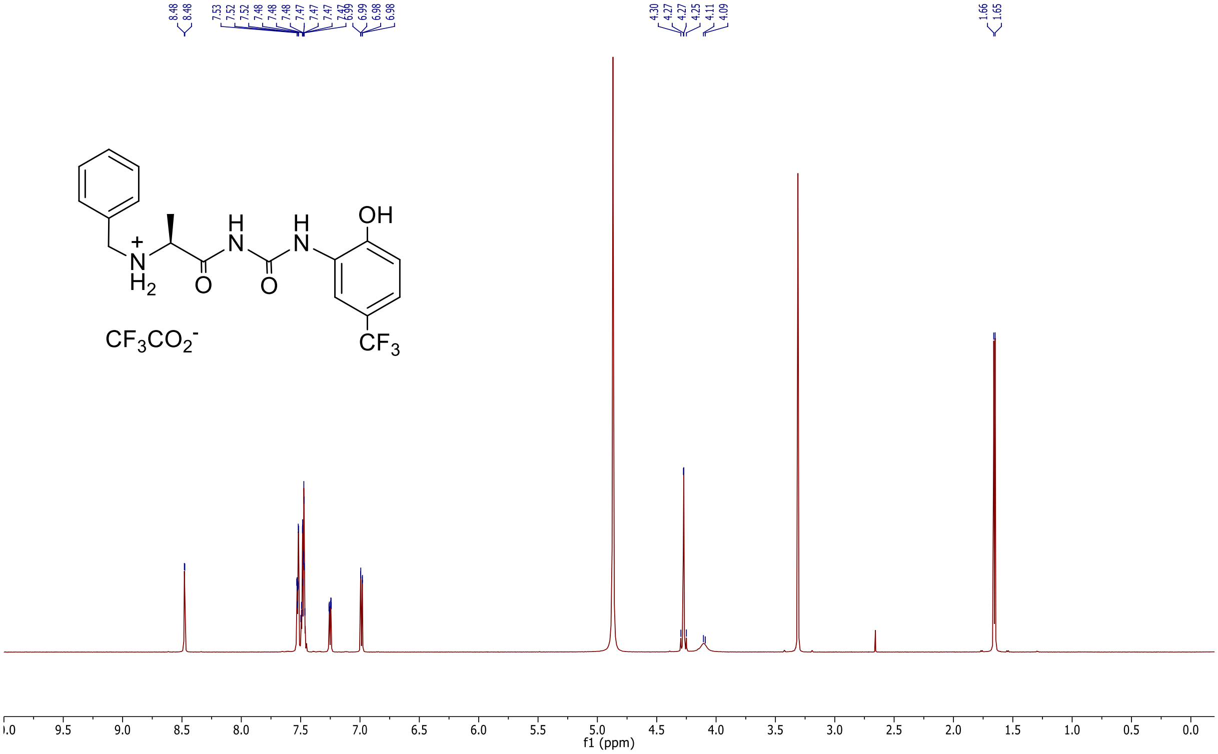

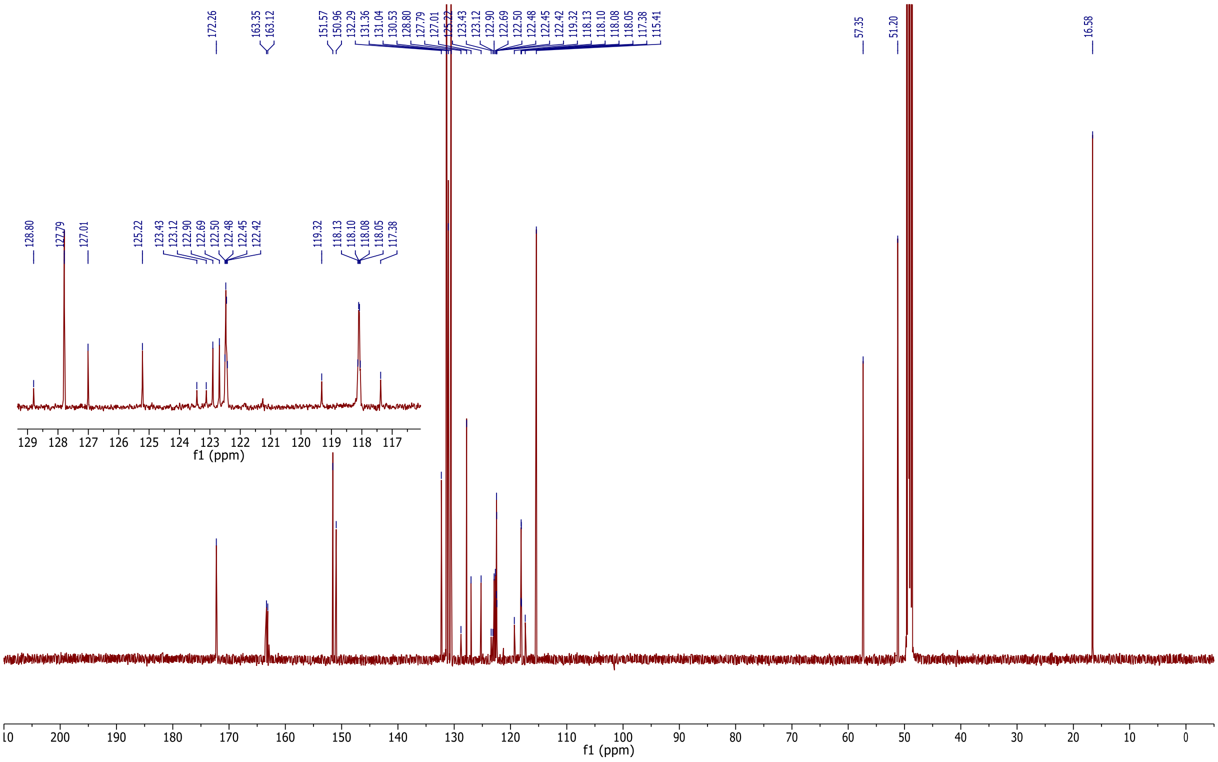

^{1}H and ^{13}C NMR Spectra ($CDCl_3$) of 10 (MC-II-153-b)

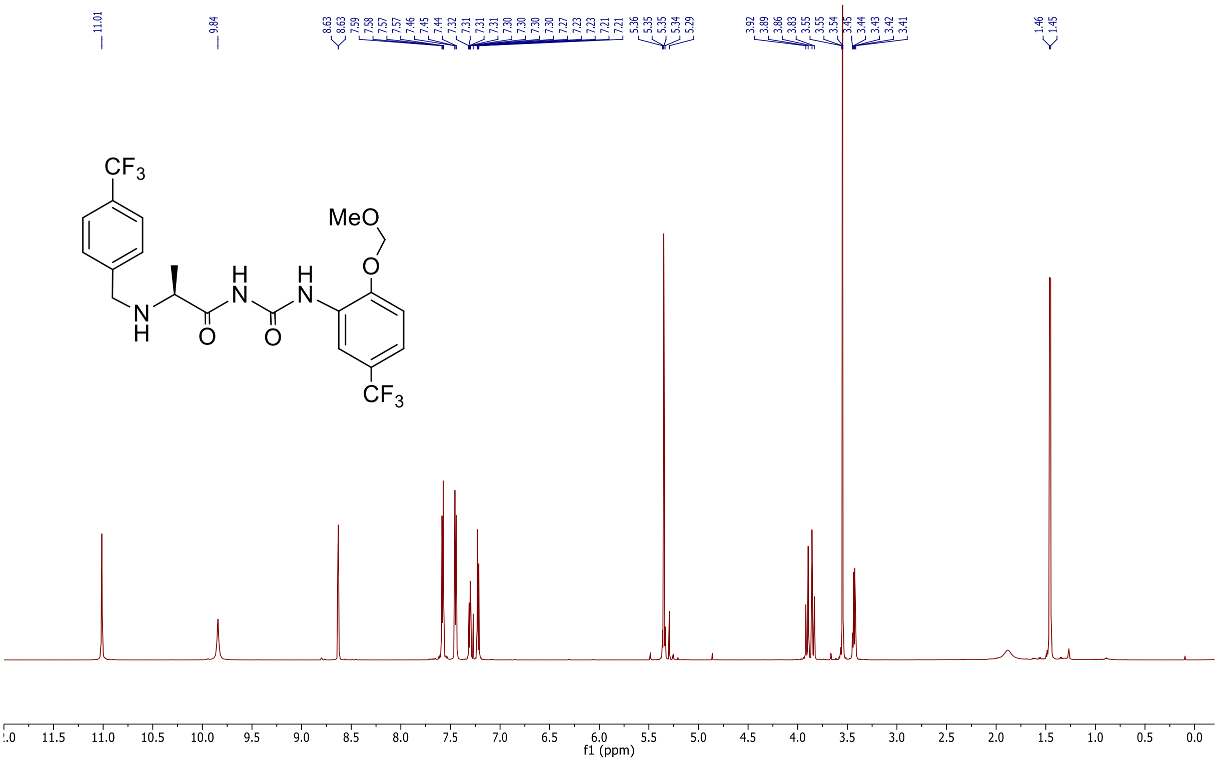

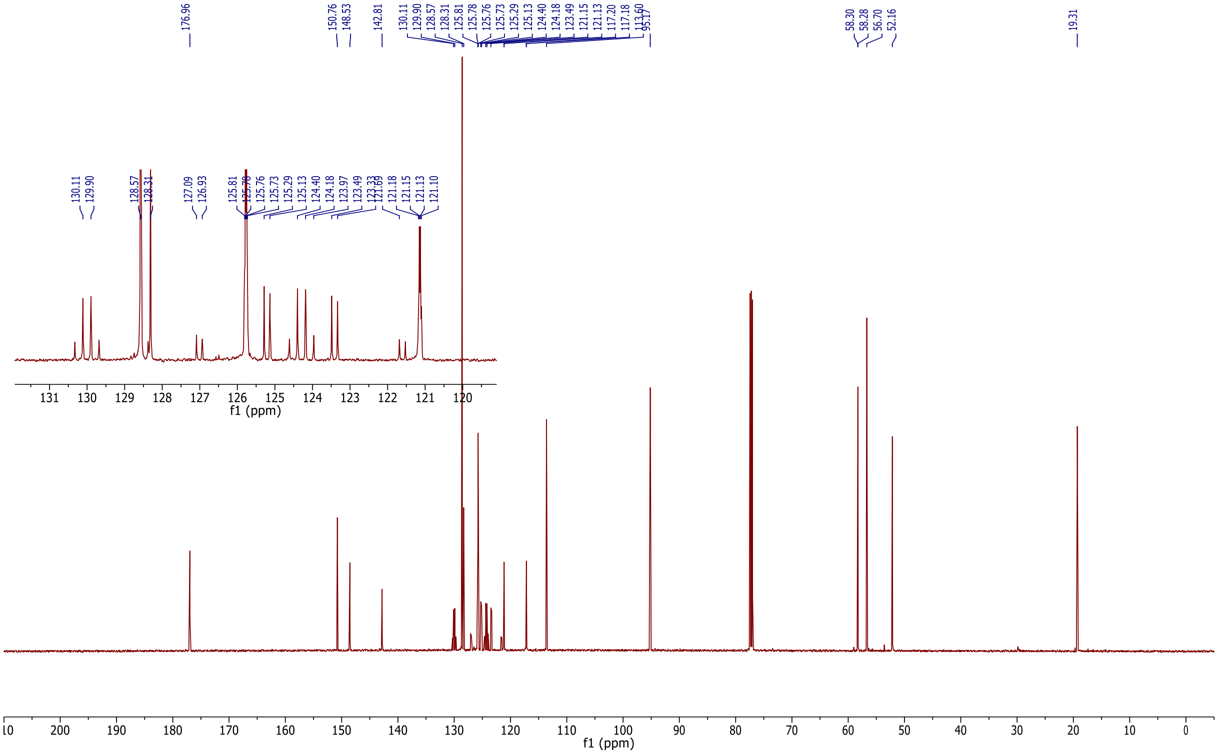

^{1}H and ^{13}C NMR Spectra (DMSO-d_6) of 14 (MC-I-169-b)

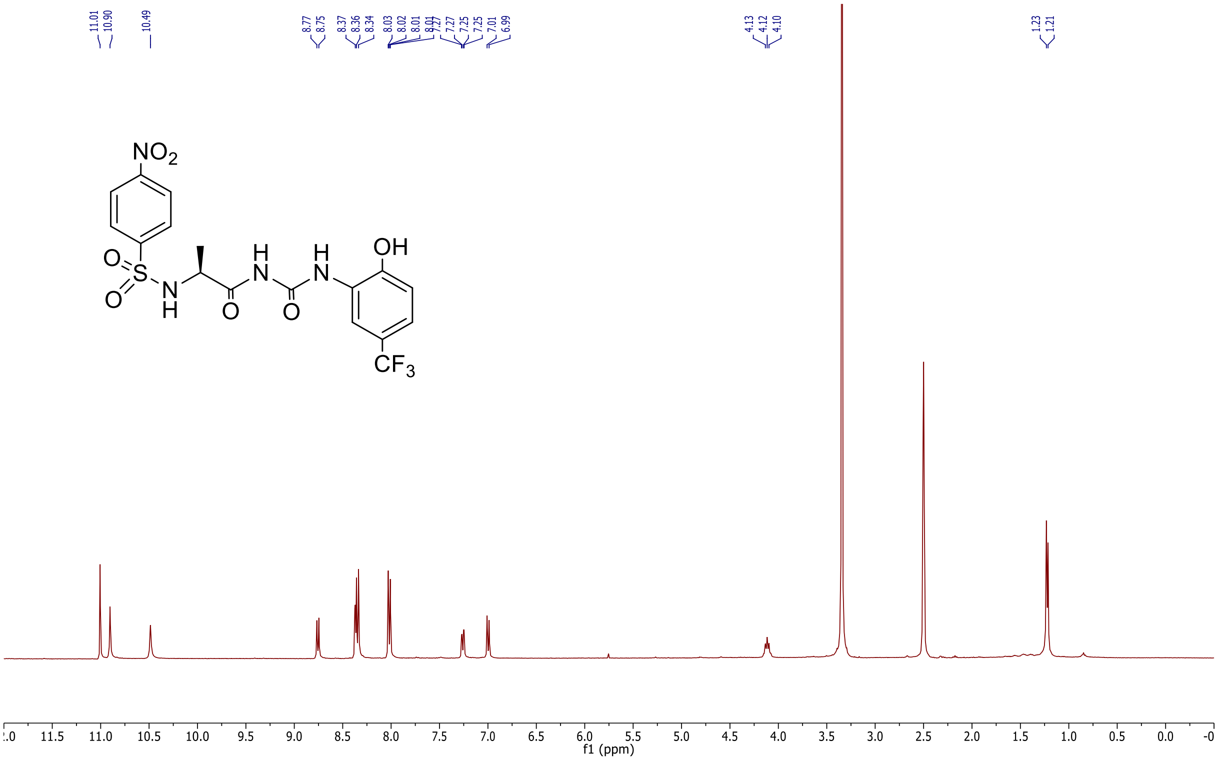

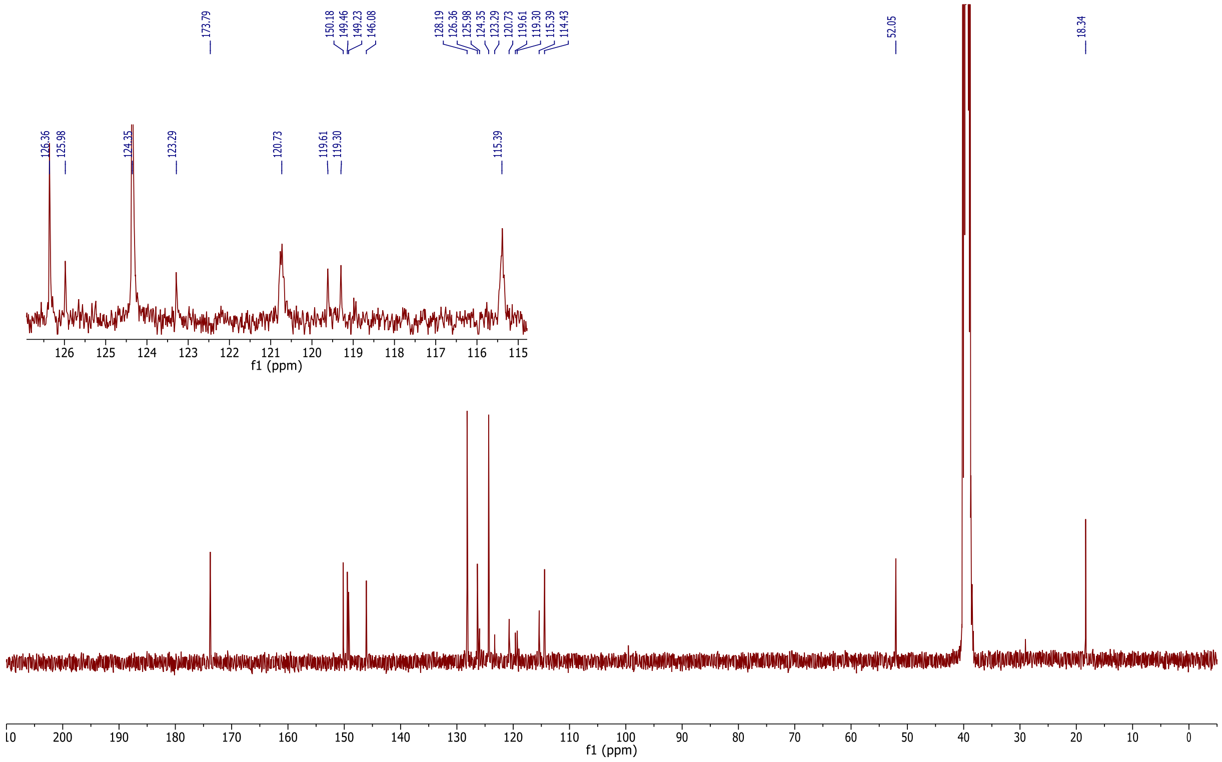

^{1}H and ^{13}C NMR Spectra ($CDCl_3$) of 17 (MC-II-67-b)

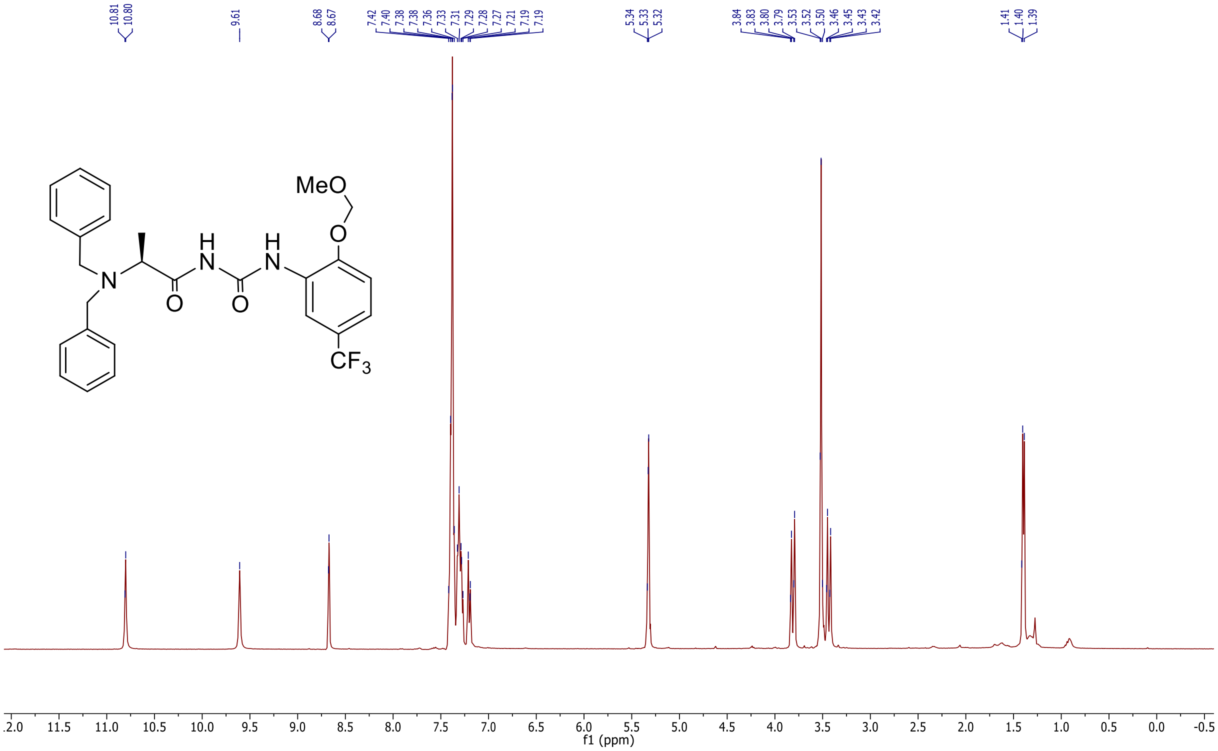

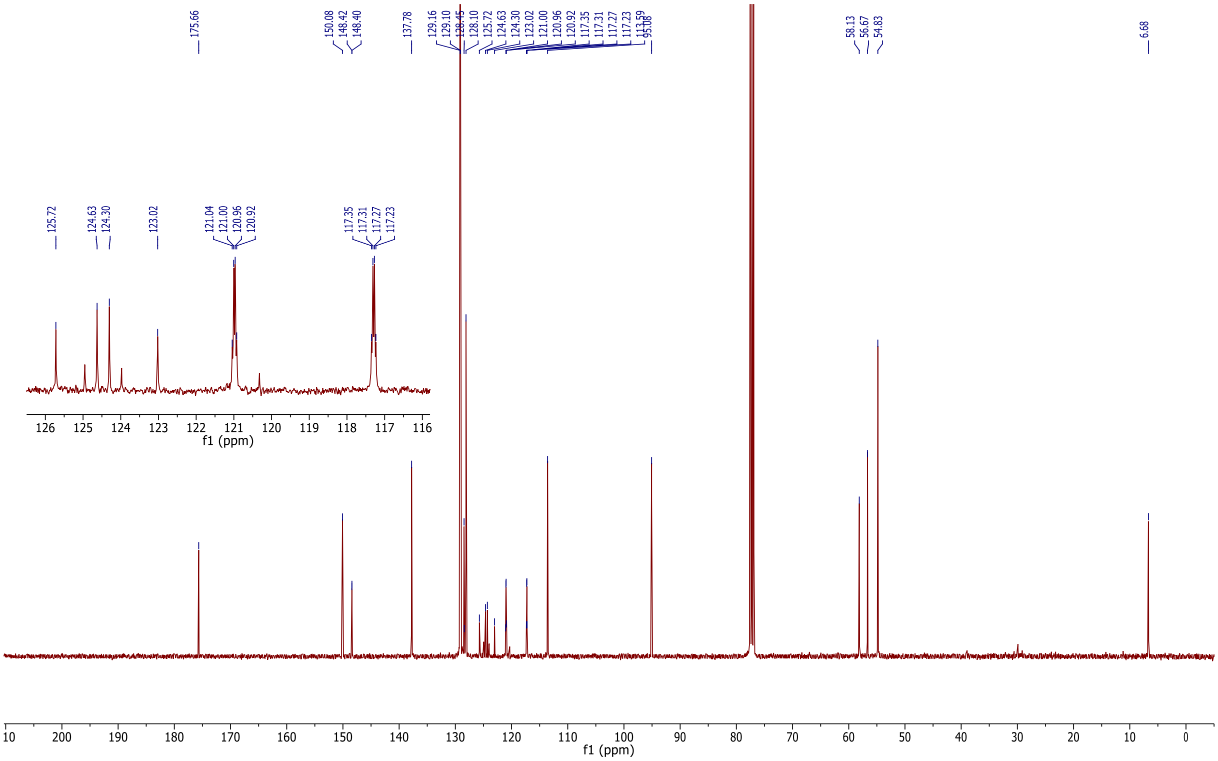

^{1}H and ^{13}C NMR Spectra ($CDCl_3$) of 18 (MC-I-165-b)

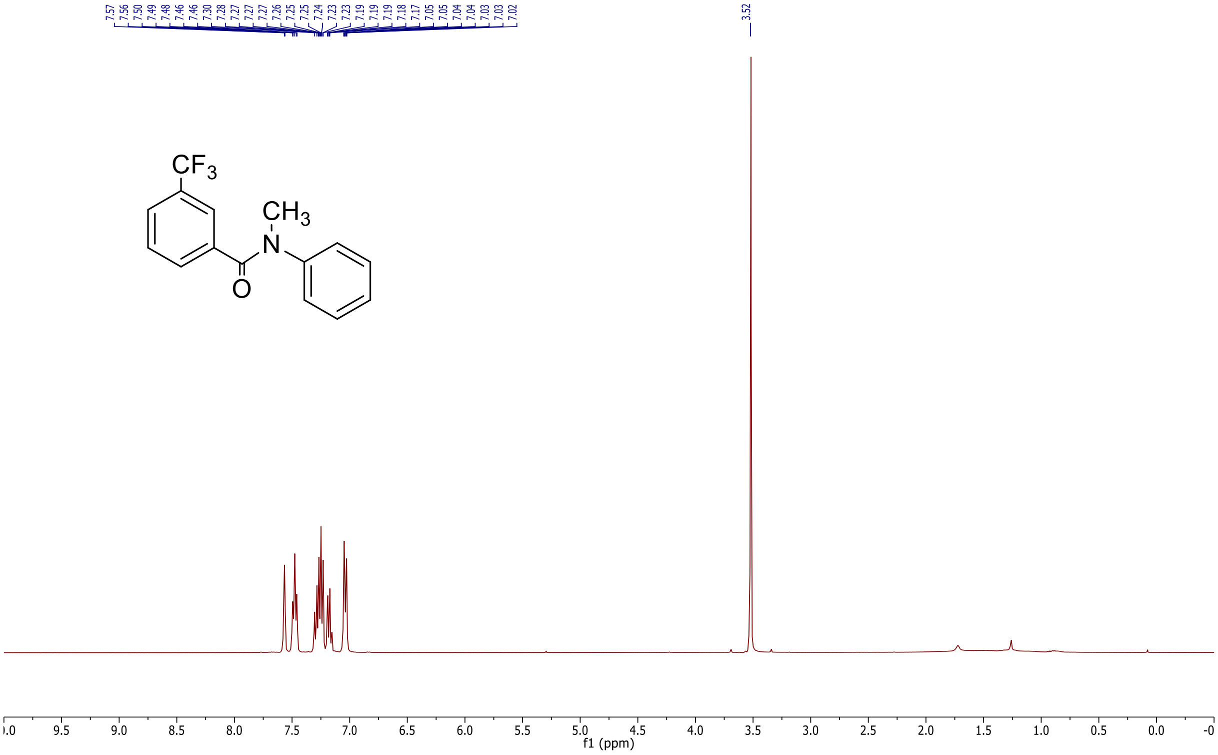

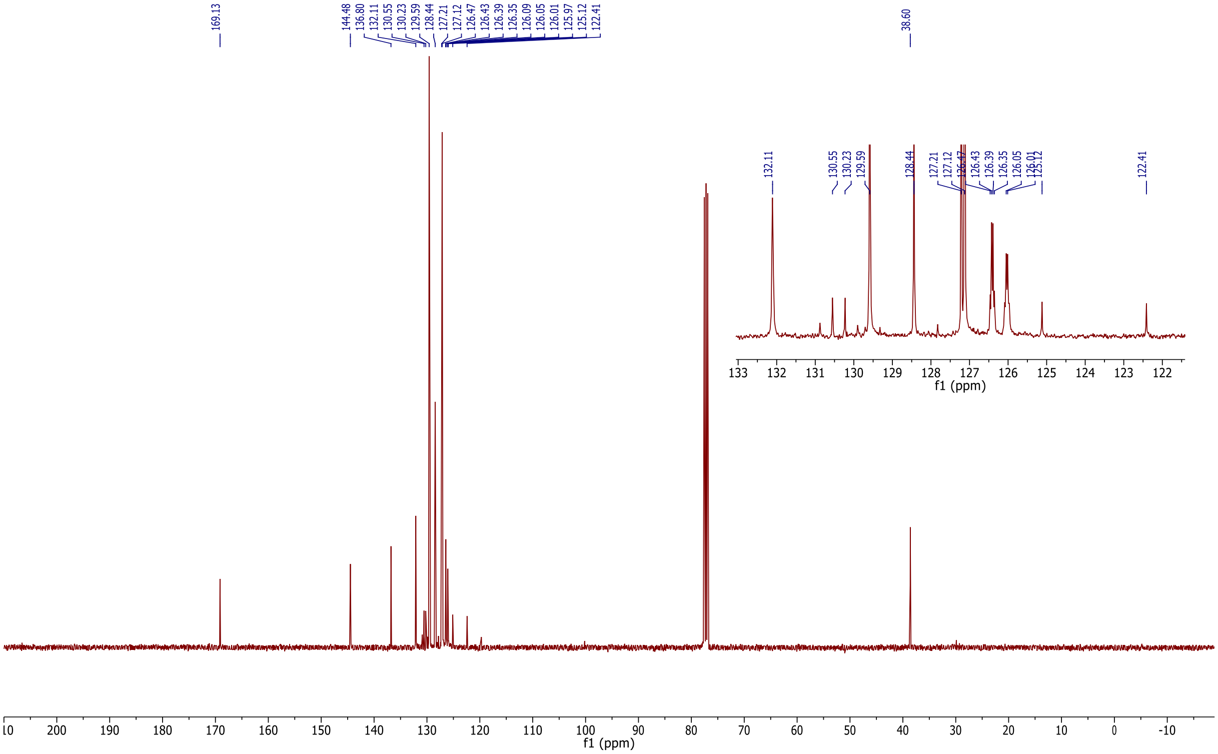

^{1}H (DMSO-d$_6$) and ^{13}C NMR (CDCl$_3$) Spectra of 23 (MC-I-161-b)

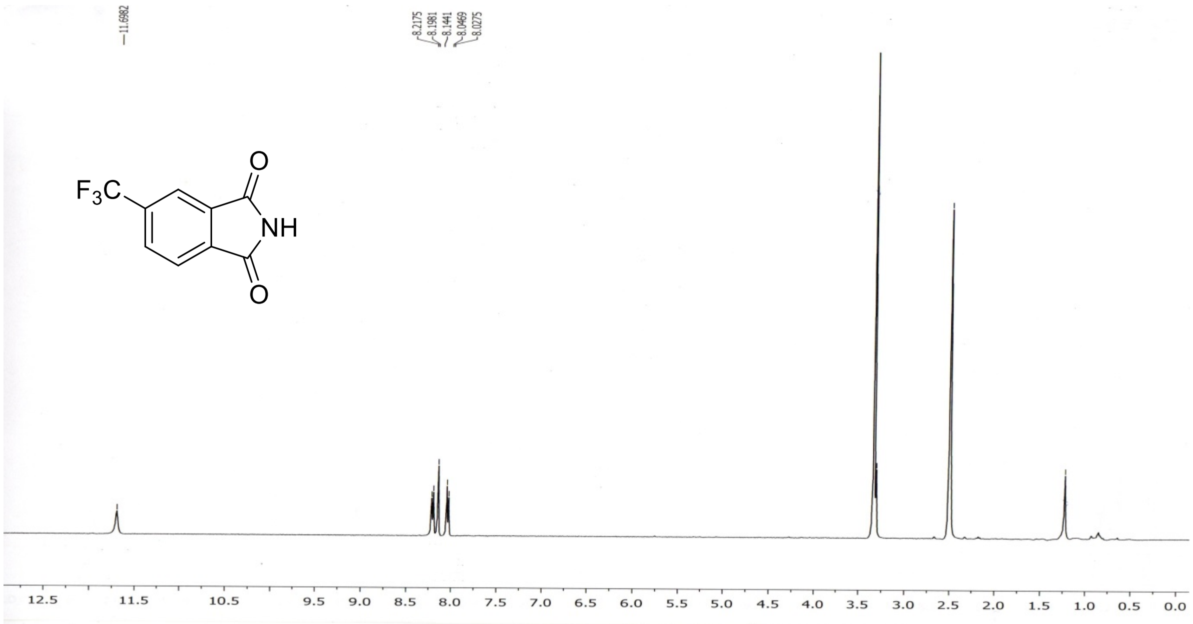

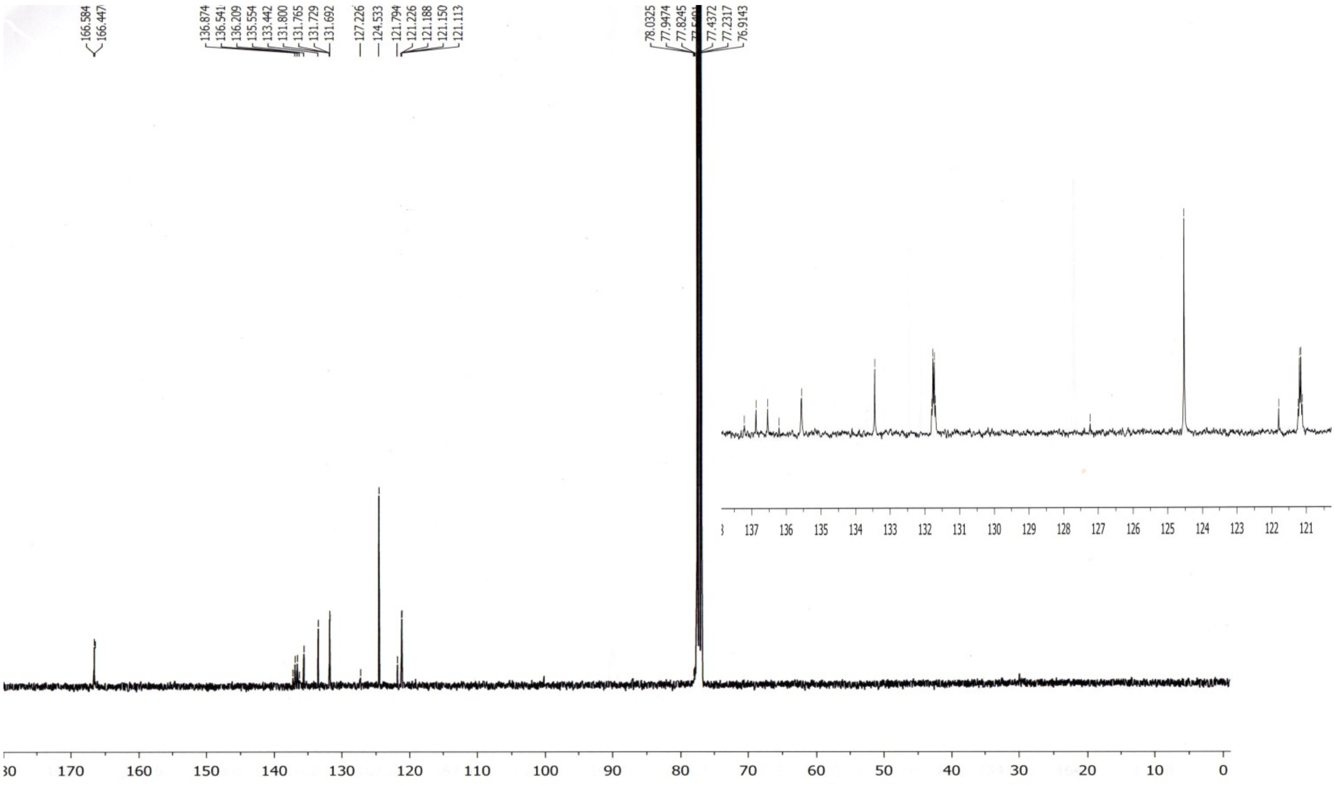

^{1}H and ^{13}C NMR (DMSO-d_6) Spectra of 24 (MC-II-17-c)

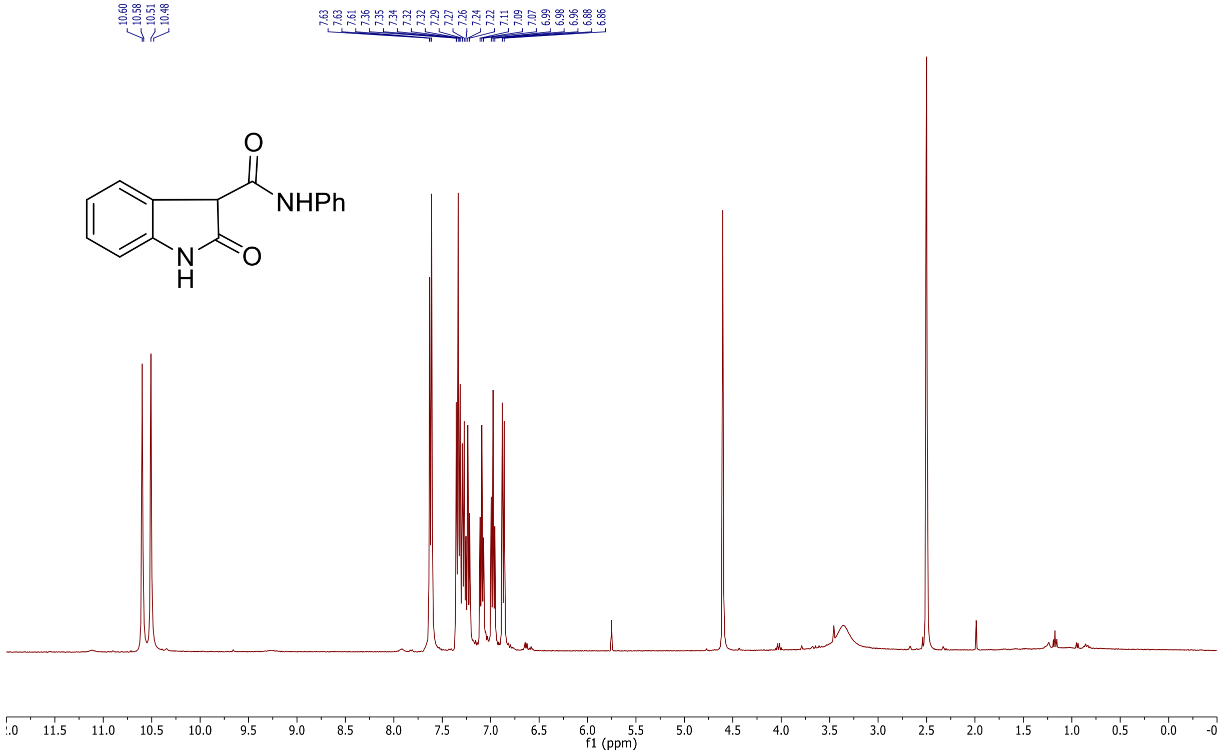

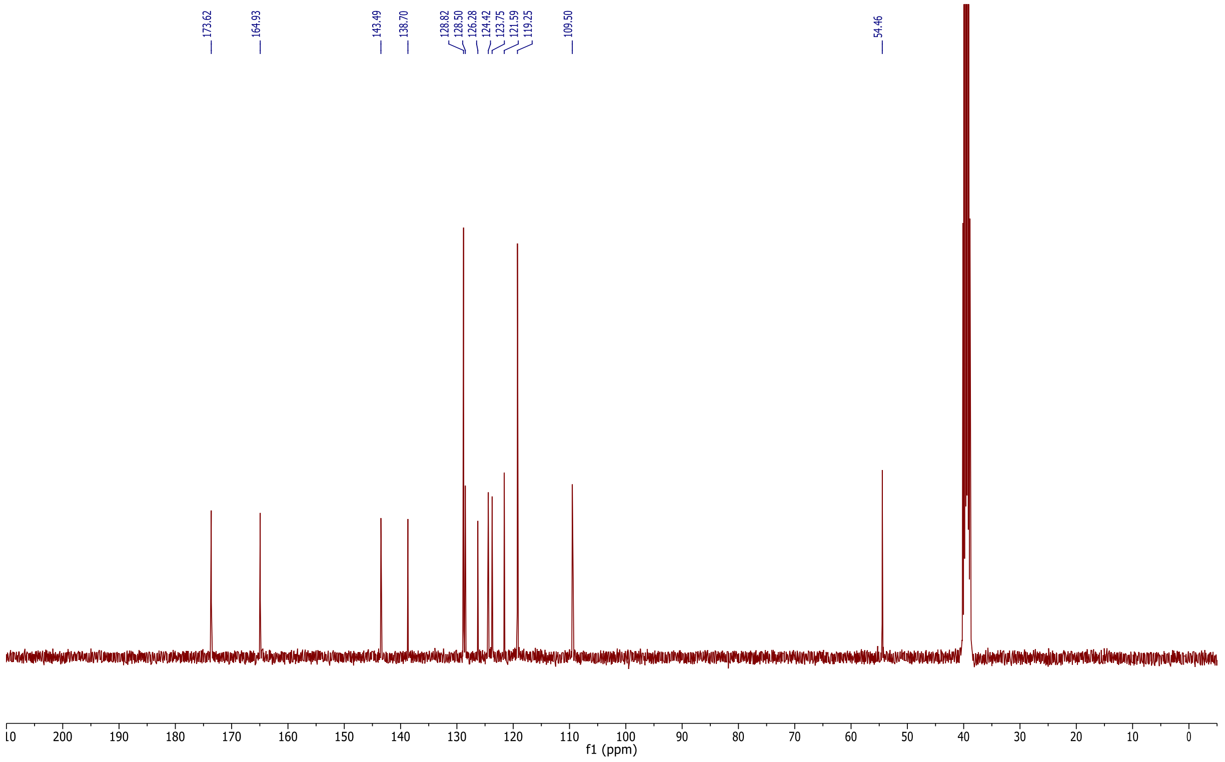

Supplement: Synthesis S1 — Chemical synthesis schemes for each compounds together with quality control data (NMR, HPLC data). (PDF) [file pone.0105553.s004.pdf]
